# Environmental sustainability of food production and consumption in the Nordic and Baltic region – a scoping review for Nordic Nutrition Recommendations 2023

**DOI:** 10.29219/fnr.v68.10539

**Published:** 2024-10-30

**Authors:** Helen Harwatt, Tim G. Benton, Jan Bengtsson, Bryndís Eva Birgisdóttir, Kerry Ann Brown, Corné van Dooren, Maijaliisa Erkkola, Morten Graversgaard, Thorhallur Halldorsson, Michael Hauschild, Anne Høyer-Lund, Jelena Meinilä, Bob van Oort, Merja Saarinen, Hanna L. Tuomisto, Ellen Trolle, Olafur Ögmundarson, Rune Blomhoff

**Affiliations:** 1 Royal Institute of International Affairs, Chatham House, London, UK; 2 Department of Energy and Technology, Swedish University of Agricultural Sciences, SLU, Uppsala, Sweden; 3 Bryndís Eva Birgisdóttir, Faculty of Food Science and Nutrition, School of Health Sciences, University of Iceland, 102 Reykjavik, Iceland; 4 Kerry Ann Brown, University of Exeter Medical School, University of Exeter, Exeter, UK; 5 World Wildlife Fund, Zeist, Netherlands; 6 Maijaliisa Erkkola, Department of Food and Nutrition, University of Helsinki, PO Box 66, 00014 Helsinki, Finland; 7 Morten Graversgaard, Department of Agroecology, Aarhus University, Blichers Allé 20, 8830, Tjele, Denmark; 8 Thorhallur Halldorsson, Faculty of Food Science and Nutrition, School of Health Sciences, University of Iceland, Reykjavik, Iceland; 9 National Food Institute, Technical University of Denmark (DTU), Kgs. Lyngby, Denmark; 10 The Norwegian Directorate of Health, Oslo, Norway; 11 Centre for International Climate Research, Oslo, Norway; 12 Natural Resources Institute, Helsinki, Finland; 13 Department of Nutrition, Institute of Basic Medical Sciences, University of Oslo, Oslo, Norway

**Keywords:** Dietary guidelines, nutrition recommendations, sustainable diets, sustainable consumption, food policy, agriculture, climate change, biodiversity loss, land use, planetary boundaries

## Abstract

This scoping review examines environmental impacts related to food production and consumption in Nordic and Baltic countries. The overarching advice to all Nordic and Baltic countries, in line with the current body of scientific literature, is to shift to a more plant-based dietary pattern and avoid food waste. Taking into account current consumption patterns, there is a high potential and necessity to shift food consumption across the countries to minimise its environmental impact. More specifically, a substantial reduction in meat and dairy consumption and increased consumption of legumes/pulses, whole grains, vegetables, fruits, nuts, and seeds are suggested as a priority intervention. Reducing the environmental impacts of seafoods is also key and suggestions include a shift to seafoods with lower environmental impacts such as seaweed and bivalves. As part of the suggested transition to a more plant-based diet, the scope for increasing the provision of plant-based foods through increasing the cultivation of legumes/pulses, vegetables, and grains and through feed-to-food shifts within the region should be explored.

## Popular scientific summary

- The environmental impacts related to food production and consumption have been assessed
- The paper takes a predominantly global perspective, while discussing the context and implications across the eight focal Nordic and Baltic countries
- The overarching advice to all eight countries is to shift to a more plant-based dietary pattern

The global food system currently exerts substantial environmental burdens, from harvesting fish and seafood from nearly every river, lake, and ocean; using around 50% of habitable land and 70% of available freshwater; causing major losses of nitrogen and phosphorous; being the main source of global anthropogenic methane emissions and accounting overall for around a third of anthropogenic greenhouse gas (GHG) emissions – driving biodiversity loss, deforestation, climate change, and eutrophication of fresh water and coastal ecosystems (1–3). On its current trajectory, food production is likely to cause more losses of biodiversity and carbon sinks such as forests, and further global temperature rise (4–7). Such impacts are already, and will increasingly, threaten human health and planetary health. For example, widespread and rapid changes in the atmosphere, ocean, cryosphere, and biosphere have occurred; and climate change is affecting many weather and climate extremes in every region across the globe – causing adverse impacts to ecosystems and human populations (8).

The planetary boundaries framework brings these major issues together to assess the current situation in relation to a set of interrelated and interacting biogeochemical boundaries at the global level – limits that if exceeded could result in large-scale abrupt (and potentially irreversible) ecosystem and climate destabilisation (9). The limits are based on evidence from earth system science in relation to how much of a given action or substance can be tolerated at the global level until regime shifts are likely triggered. Some boundary limits (e.g. climate) are based on evidence of large-scale (i.e. global, continental or regional) threshold behaviours, whereas other boundary limits (e.g. biodiversity and freshwater) are based on their interaction with sub-global or regional processes and boundaries. Assessments suggest that 6 of 9 planetary boundaries have exceeded a safe limit [climate change, land use change, biodiversity loss, biogeochemical flows (nitrogen and phosphorus), freshwater and novel entities (including plastics)] (9–11). A more recent analysis of spatial variability in both ecosystems’ sensitivity to nitrogen pollution and agricultural nitrogen losses found that the aggregated global surplus boundary for nitrogen is far exceeded by the current nitrogen surplus (12). Although not without its limitations (13), or criticisms – including those relating to the difficulty in defining global ecosystem thresholds for local environmental impacts (14), the planetary boundaries concept uniquely and visually provides an understanding and broad framing regarding key environmental limits within which food systems should operate, beyond the limited scope of climate change. We use the planetary boundaries framework in this paper to explore a range of environmental impacts in a comparative way across the Nordic and Baltic countries rather than as a predictive or prescriptive tool, and provide background data and methodologies to enable comparisons with other measures of sustainability (see Box 1 and Appendices 1–3). The difference between countries in terms of impacts should not be interpreted as a lack of need to reduce impacts in countries with relatively small impacts or to focus attention solely on countries with relatively large impacts.

**Fig. 1:**
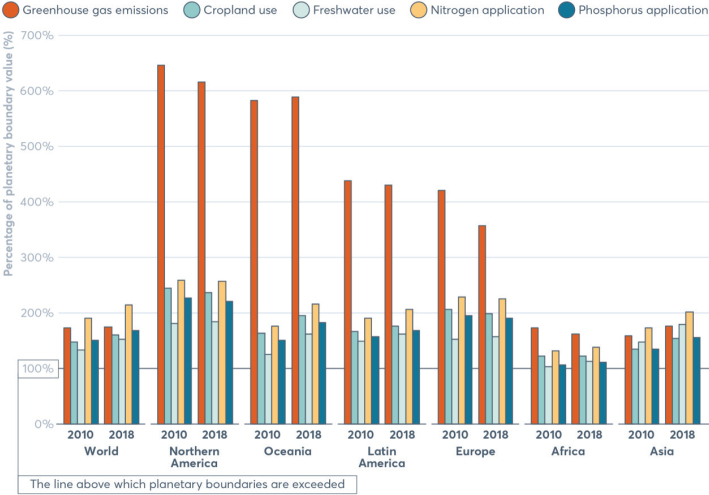
Global sustainability test comparing impacts of the food system with five global environmental targets^1^ Source: *Global Nutrition Report 2021 (**17**)^2^* The analysis utilises country-specific food consumption and environmental footprint data and relates them to the food portions of global environmental targets that is a global test to assess the impacts if everyone in the world consumed at the given rate. The methods and data are described in Box 1 and presented in Appendices 1–2.

**Fig. 2:**
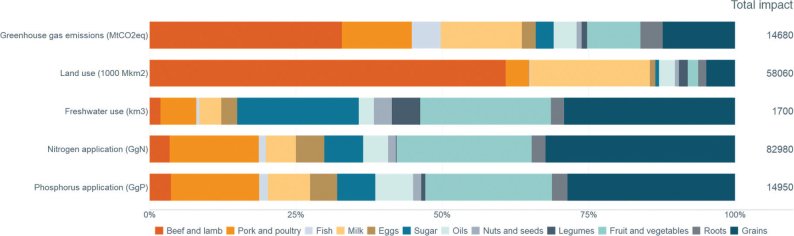
Environmental impacts of the global food system by type of impact and food group as a proportion of total impact Source: Global Nutrition Report 2021 (17). The analysis utilises country-specific food consumption and environmental footprint data. The methods and data are presented and described in Box 1 and Appendix 2.

No region in the world is on course to meet the food-related portion of global environmental targets, indicating that the global food system is exceeding a safe trajectory to stay within the planetary boundaries (as indicated in Fig. 1, where all 2018 impacts are above 100% in each region). In this test, regional diets in 2010 and 2018 are globally adopted and compared to global environmental targets (15). Despite a pressing need to reduce environmental burdens from all sectors, global impacts from the food system have increased by up to 14% over the past decade alone and have contributed to the exceedance of five global environmental thresholds (15). For example, in 2018, food-related GHG emissions at the global level exceeded the limit consistent with keeping warming below 2°C by 74% (Fig. 1). Cropland use was 60% above the value aligning with limiting the loss of natural habitat according to Aichi Biodiversity Targets (SDG 15). Freshwater use exceeded sustainable withdrawals by over 52% (SDG 6.4). Nitrogen and phosphorus application exceeded values (by 113% and 67% respectively) that would limit marine pollution to acceptable levels (as defined in SDG 14.1). Hence, major changes are required across all parts of the food system. Figure 2 shows the relative proportion of global environmental impacts from each food type, with beef accounting for the majority of land use and the largest proportion of GHGs, and grains accounting for the largest proportion of freshwater, nitrogen, and phosphorus use.

It is important to include the environmental impacts of imports from exporting countries and not limit sustainability considerations to local production as this can skew ‘rankings’ or perceptions of sustainability. For example, the Sustainable Development Report shows a very different performance score when ‘spillover’ impacts are included in an overall score that measures the total progress towards achieving all 17 SDGs and the impact of each country’s actions on other countries’ abilities to achieve the SDGs. When factoring in spillovers including ‘environmental and social impacts embodied into trade’, Finland, Denmark, Sweden, and Norway shift from their top 4 ranking positions globally on progress towards meeting SDGs to 124, 137, 139, and 146 global rankings respectively (16). The impacts of imports could differ substantially depending also on agricultural production standards in exporting countries, such as nitrogen use and types of permitted plant protection products. Politically, however, there could be a preference to import goods as their environmental impacts are not included in national inventories of importing countries (e.g. in the reporting of national GHGs under the UNFCCC). Such accounting preferences are not beneficial in terms of reducing overall environmental burdens from the food system.

Tackling the planetary health crisis requires extraordinary levels of action across all sectors at an unprecedented speed. According to the latest assessment from the Intergovernmental Panel on Climate Change (IPCC), ‘Rapid and far-reaching transitions across all sectors and systems are necessary to achieve deep and sustained emissions reductions and secure a liveable and sustainable future for all’ (8). Taking transformative action this decade, including in most cases immediate emissions reductions in all sectors, would not only reduce projected adverse impacts on humans and ecosystems but would deliver many co-benefits, especially for human health. Taking such actions in the near-term typically requires a range of enabling policies to provide high up-front investments and lessen potentially disruptive changes (8). Transforming food systems is a critical part of meeting planetary health goals. A substantial departure from business-as-usual expectations is required, and a shift in the roles and responsibilities of public sector actors versus businesses in shaping dietary demand (18). This is even more apparent if taking into account safe *and* just earth system boundaries (ESBs) that minimise humans’ exposure to significant harm from Earth system change (by comparison, the PBs identify only safe biophysical boundaries). A recent analysis at global and sub-global scales found that seven of eight globally quantified safe and just ESBs and at least two regional safe and just ESBs in over half of the global land area are already exceeded. Ensuring human well-being thus requires systemic transformations across energy, food, urban, and other sectors. Addressing the drivers of Earth system change is also required (including the economic, technological, and political), together with an increased understanding of the role of justice, economics, technology, and global cooperation in creating a safe and just future (19).

Box 1 Environmental data sources and handling methods.

**Fig. 3:**
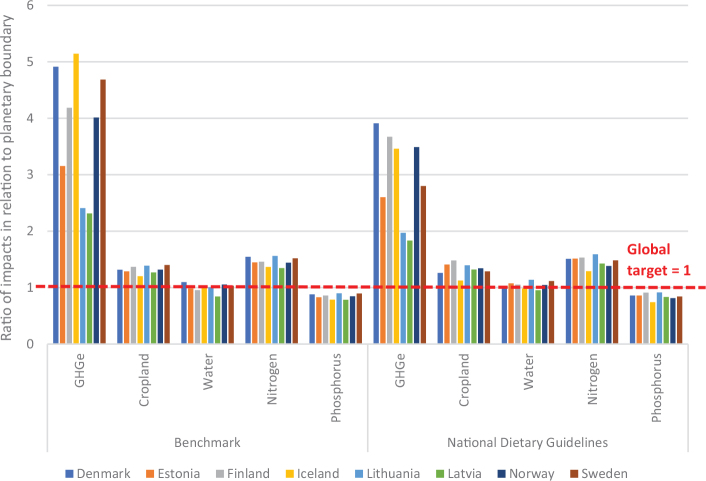
Impacts of current (benchmark) diets and adopting current national dietary guidelines in relation to the food-related portions of global environmental targets: by country and environmental metric.^13^ Source data: Springmann, M, Spajic, L, Clark, MA, Poore, J, Hertforth, A, Webb, P, Rayner, M, Scarborough, P. The healthiness and sustainability of national and global food-based dietary guidelines: modelling study (25). The analysis utilises country-specific environmental footprints described in Box 1 and Appendices 1–3.

**Fig. 4:**
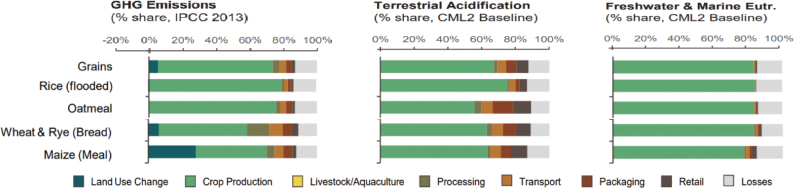
Proportion of environmental impacts from different life cycle stages: Cereals (global) (2).

**Fig. 5:**
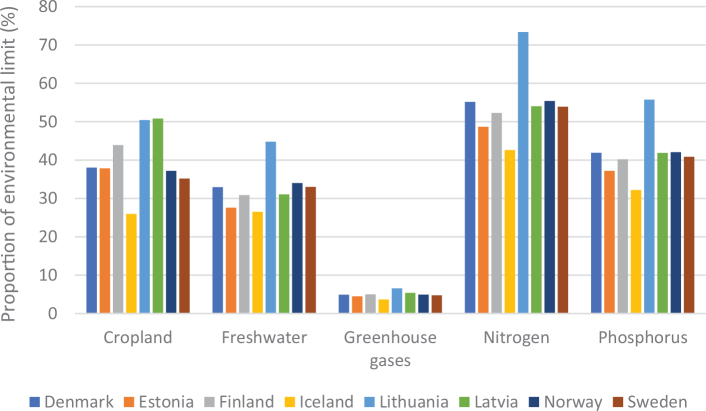
Impacts of cereal consumption across the Nordic and Baltic countries in relation to the food portion of global environmental limits (%). *Source: Global Nutrition Report 2021 (17).* The analysis utilises country-specific food consumption and environmental footprint data and relates them to the food portions of global environmental limits that is a global test to assess the impacts if everyone in the world consumed at the given rate. The methods and data are described in Box 1 and presented in Appendices 1–3.

**Fig. 6:**
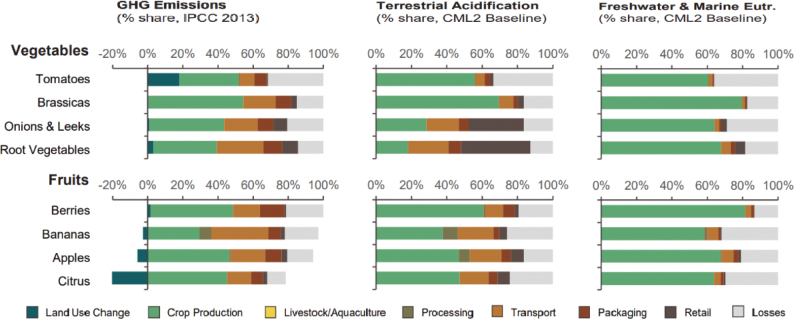
Proportion of environmental impacts from different life cycle stages: Vegetables, fruits and berries (global) (2).^18^

**Fig. 7:**
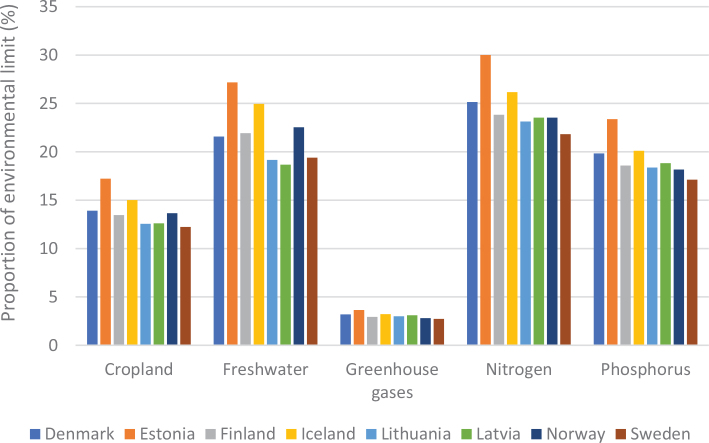
Impacts of fruit and vegetable consumption across the Nordic and Baltic countries in relation to the food portion of global environmental limits (%) *Source: Global Nutrition Report 2021 (**17**).* The analysis utilises country-specific food consumption and environmental footprint data and relates them to the food portions of global environmental limits that is a global test to assess the impacts if everyone in the world consumed at the given rate. The methods and data are described in Box 1 and presented in Appendices 1–3.

**Fig. 8:**
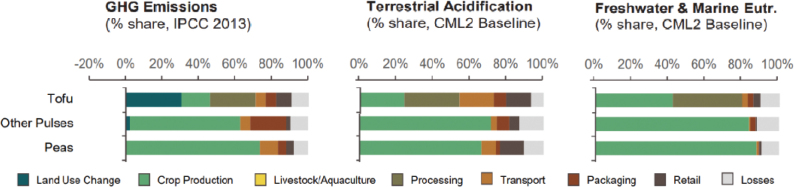
Proportion of environmental impacts from different life cycle stages: Pulses (legumes) (global) (2).

- Data on food production, imports, exports, and food supply quantity (kg per person per year) for all 8 countries are provided in most food group chapters, in Tables 4, 5, 6, 7, 9–15, 16, 17, 19–21, 23, 25, 27, 28, 30, 32, and 33. The data were sourced from FAOSTAT Food Balance Sheets (FBS) for the year 2019. The FAO provides the following description: ‘FBS are compiled every year by FAO, mainly with country-level data on the production and trade of food commodities. Using these data and the available information on seed rates, waste coefficients, stock changes, and types of utilisation (feed, food, processing, and other utilisation), a supply/utilisation account is prepared for each commodity in weight terms. The food component of the commodity account, which is usually derived as a balancing item, refers to the total amount of the commodity available for human consumption during the year. Besides commodity-by-commodity information, the FAO FBS also provide total food availability estimates by aggregating the food component of all commodities including fishery products. From these values and the available population estimates, the per-person dietary energy, protein, and fat supplies are derived and expressed daily. In the FBS, production data refer only to primary products while data for all other elements also include processed products derived there from, expressed in primary commodity equivalent’ (23). Food production is reported at the farm level for crop and livestock products (i.e. in the case of crops, excluding harvesting losses) and in terms of live weight for fish items (i.e. the actual ex-water weight at the time of the catch). Data are expressed in terms of dressed carcass weight, excluding offal and slaughter fats. Production of beef and buffalo meat includes veal; mutton and goat meat includes meat from lambs; pig meat includes bacon and ham in fresh equivalent. Poultry meat includes meat from all domestic birds and refers, wherever possible, to ready-to-cook weight. Per capita supply figures represent only the average supply available for the population as a whole and do not necessarily indicate what is actually consumed by individuals. Even if they are taken as approximation to per capita consumption, it is important to note that the amount of food actually consumed may be lower than the quantity shown in the FBS supply figures, depending on the degree of losses of edible food and nutrients in the household, for example, during storage, in preparation, and cooking (24). For this reason, where we use FBS food supply data in this paper, we refer to it as food supply, rather than food consumption.
- Every year, authorities in over 245 countries and territories submit national food and agriculture statistics, as well as micro datasets collected through farm and household surveys, to FAO. In the FAOSTAT database, national food supply is estimated as average per capita foods available for consumption based on domestic production adjusted for exports, imports, and non-food uses. National authorities, who annually supply data to FAOSTAT, are not required to use standardised methodologies when collecting, categorising and grouping data, which is a limitation of using FAOSTAT data in country-specific studies.
- To analyse the environmental impact of food availability, country-specific data from FAOSTAT are often paired with comprehensive country-specific databases of environmental footprints or life cycle assessment data that, to various degrees, include primary production, farmed animal feed requirements, processing, transport, and packaging, and may also consider food loss and waste. These models build on numerous assumptions and theoretical considerations. Despite commonly held limitations with all types of complex modelling endeavours, the models of environmental footprints that we refer to in this paper are innovative and represent the state-of-the-art at a level suitable for providing an indicative assessment of the environmental impacts of food consumption across countries and cross-country comparisons.
- In epidemiological national dietary surveys, 24-h recalls, food records, and Food Frequency Questionnaires are commonly used as evaluated methods of dietary assessment. In this paper, the country-specific food consumption, based on such methodologies, is compared to national dietary guidelines in Table 2. However, due to the differences in methodologies, food consumption data in Table 2 sourced from dietary surveys is not necessarily directly comparable with food supply data used in this paper, sourced from the FAOSTAT FBS. There are advantages and disadvantages with both datasets.
- In this paper, we use datasets from four analyses to demonstrate the environmental impacts of foods and food consumption at global, regional, and national levels – and in some cases, how they relate to the food portion of planetary boundaries: i) Springmann et al. (Nature, 2018), ii) Springmann et al. (BMJ, 2020), iii) Global Nutrition Report 2021, and iv) Poore and Nemecek (Science, 2018) (2, 14, 17, 25). Food supply corrected for waste (from FAO FBS) is used as an estimation of food consumption. When interpreting these data, it is important to understand that food consumption estimated from food supply in FBS may vary from the food consumption assessed by food records or 24-h diet recall methodologies. In relation to i) and ii) above: Springmannet al. (14) and Springmannet al. (25) used the same environmental footprints and data sources. Country-specific environmental footprints were calculated by combining national food consumption data with data on country-specific environmental footprints for crop, livestock, and fish production. Specifically, food consumption was calculated from the FAOSTAT FBS database as country-specific food supply data adjusted for waste (see Appendices 1–3). The environmental footprint data were calculated as follows:
  - GHG emissions:
    - Data on crop-related GHG emissions were taken from Carlsonet al. (Nature Climate Change 2017). Carlsonet al. compiled crop harvest and management data at the national and subnational level from > 15,000 units in the world, ranging from countries, states, and countries, as well as agricultural data from FAOSTAT [Monfredaet al. Glob. Biogeochem. Cycles 22, GB1022 (2008) and Ramankuttyet al. Glob. Biogeochem. Cycles 22, GB1003 (2008)]. These data provide a crop-specific national and subnational assessment of how agricultural management practices interact with biophysical characteristics to generate heterogeneous patterns of resources used for crop production. Country-specific GHG emissions from crops were then calculated from these data by using standard IPCC Tier 1 methodology for GHG emissions.
    - Data on livestock-related GHG emissions (including feed related emissions) were taken from Tubielloet al. (Environ. Res. Lett 2013). They compiled a global emissions database with country level details based on an inventory-based, bottom-up accounting of activity data from FAOSTAT (2012) and GHG emissions using Tier 1 IPCC methodology.
    - GHG emissions from farmed and wild-caught fish were calculated from data on fish production from Troellet al. (PNAS 2014), Chenet al. (WorldFish Center and Int. Food Policy Res. Inst. 2017) and Troellet al. (WorldFish Center and Int. Food Policy Res. Inst., 2014) and GHG emissions using IPCC methodology (Rosegrant et al., Int. Food Policy Res. Inst, 2017).
  - Country-specific data on cropland and blue water use were adopted from the IMPACT model (Robinsonet al. IFPRI 2015). These data include feed requirements for farmed animals in terrestrial and aquatic systems.
- iii): The Global Nutrition Report 2021 uses estimates of food demand calculated from FAO FBS and a database of country and food group-specific environmental footprints from i) Springmannet al. Nature 2018 and iv) Poore & Nemecek. Science 2018 (see Appendices 1–3). Data on food demand for each country from the FAO was paired with a comprehensive database of environmental footprints, differentiated by country, food group, and environmental impact. The footprints take into account all food production, including inputs such as fertilisers and feed, transport, and processing, for example, oil seeds to oils and sugar crops to sugars.
- In this paper, we use data/analyses from i) Springmann et al., 2018 (14) (Nature, 2018), ii) Springmann et al., 2020 (25), and iii) Global Nutrition Report 2021 (17), in Figs. 1, 2, 3, 5, 7, 10, 16, 21, 23, 25, and 29 (see Appendices 1–3). These data represent food consumption (not food supply). Figures 5, 7, 10, 16, 21, 23, 25, and 29 were created using data on planetary boundary impacts for each of the eight countries from the GNR 2021 excel sheet (tab ‘country environment’) (17). Figure 3 was created using supplementary information from Springmann et al., 2020 (25) available at https://www.bmj.com/highwire/filestream/1031038/field_highwire_adjunct_files/2/sprm054404.ww3.xlsx. The Excel sheet (‘global targets’ tab) contains an overview of the country-level results in terms of current food consumption and various dietary shift scenarios in relation to planetary boundaries (25).
- iv): Most of the food group chapters contain environmental impact data for relevant foods from Poore and Nemecek 2018 (2). Figures 4, 6, 8, 20, 22, 24, 26, 28, 30, and 32 are drawn from Figure S13, which provides the proportion of GHG emissions, acidification, and eutrophication by stage of the supply chain by product. The supply chain in this dataset begins with the extraction of resources needed to produce inputs for agricultural production, the initial impact of choice by farmers, and ends at the retail store, the point of choice for consumers. The data set covers ~38,700 commercially viable farms in 119 countries and 40 products representing ~90% of global protein and calorie consumption. It covers five important environmental impact indicators: land use; freshwater withdrawals weighted by local water scarcity; and GHG, acidifying, and eutrophying emissions. Land use was calculated from inverse yield and occupation time. Occupation time is reduced by multiple cropping but increased by fallow requirements. Land use is seed, on- and off-farm arable and permanent crops, fallow land, temporary pasture, and permanent pasture. GHGs are CO_2_, CH_4_, N_2_O to air, using IPCC AR5 100-year factors with climate-carbon feedbacks. Acidification is SO_2_, NH_3_, NOx to air, and eutrophication is NH_3_, NOx to air, NO_3_ –, NH_4_ +, P, N to water; both using CML2 Baseline for characterisation factors (26). Freshwater withdrawals and scarcity-weighted freshwater withdrawals are both irrigation, drinking, pond, and processing water. These five environmental impacts are provided per kg of retail weight in most food chapters, in Tables 3, 8, 13, 18, 22, 24, 26, 29, 31, and 34 (drawn from supplementary data file (aaq0216_datas2.xls), using mean quantities from tab ‘results – retail weight’ (2)).

This paper is the result of an expert elicitation developed as a collaboration between the Nordic Nutrition Recommendations (NNR)2023 project, Chatham House, and Nordic and Baltic scientists. Helen Harwatt, Chatham House, is lead author, and led the project with Tim Benton, Chatham House, as co-author. The contributing experts (JB, RB, BEB, KAB, CvD, ME, MG, TH, MH, AH, JM, BvO, MS, ET, and OÖ) have given significant scientific input relevant to the local context, while the members of the NNR Committee (RB, ET, TH, ME, and AH) have ascertained that the content is relevant to and within the scope of the NNR project. This paper, in addition to several additional papers and other major reports, will assist the NNR Committee when formulating science advice to the authorities.

## Aims of the paper

This paper examines environmental impacts related to current food production and consumption from the perspective of identifying problems and solutions. Because demand and supply are inextricably connected, for useful context we provide an overview of food production (including imports and exports), and consumption in relation to each major food group. We take a predominantly global perspective, while discussing the context and implications across the eight focal Nordic and Baltic countries (Denmark, Finland, Iceland, Norway, Sweden, Estonia, Latvia, and Lithuania). The global perspective is mostly relevant to high-income country settings, such as those across the Nordic and Baltic region. An overview of environmental sustainability considerations is provided for each food group within the Nordic Nutrition Recommendations (NNR), from these two perspectives (global and regional). From this assessment, we provide guidance for reducing the environmental impacts of food consumption. We identify considerations for reducing environmental impacts in each food group; however, the overarching consideration is to reduce the absolute environmental burden at the food system level, and where possible to reduce the absolute environmental impacts of food groups while recognising that some need to increase (e.g. fruits and vegetables) to cover nutritional needs and improve population health. The provision of prescriptive and detailed advice for each individual country is beyond the scope of this paper, as are recommendations on achieving sustainable food production. Similarly, we are aware that some of the focal countries place an importance on self-sufficiency in terms of food production, and a number of analyses have explored its prospects (20–22). While it might be possible to meet both environmental and self-sufficiency targets in some contexts and countries, self-sufficiency is not synonymous with sustainable food consumption. Also, for some countries (e.g. Iceland), growing enough food to feed the entire country is not a realistic option, even if it was desirable from an environmental sustainability perspective. The complexity of reconciling self-sufficiency goals with national diets and environmental sustainability from the perspective of planetary boundaries is beyond the scope of this paper.

### Research approach

Both between and within the countries, it is difficult to fully consider the great variation in agricultural structure and production, topographic and climate conditions, and land use (20). Given this consideration, combined with the unavailability of a comprehensive dataset on the spectrum of environmental impacts of food types consumed in the Nordic and Baltic region (e.g. biodiversity impacts across all food groups), we approached this assessment partly as an expert elicitation to ensure that the rich body of existing data on environmental impacts of foods and diets could be best interpreted within the context of the Nordic and Baltic region, and hence, within the project constraints, the contents would be as relevant as possible to the NNR2023. We collected input from 20 regional experts through a series of three workshops and via multiple reviews of the manuscript. The workshops were used to discuss the paper and its content in detail, and environmental sustainability issues within the region of relevance to the NNR food groups, including those global in scope, with focussed breakout sessions on meat, dairy, fruits and vegetables, pulses, fish and seafood, and ultra processed foods. The experts also contributed a range of specialist knowledge in relation to environmental impacts of food production and consumption across the region during three subsequent reviews of the paper. A workshop with all co-authors was also convened before the paper went to the public hearing. To maximise the confidence of our recommendations, in addition to the expert elicitation method, we draw from existing analyses and reviews from the scientific literature and refer to research conducted in the Nordic and Baltic region.

The 20 regional experts (see acknowledgements) were appointed by the NNR2023 Committee based on a public call and after careful evaluation of each expert’s competence and experience. To supplement the call, experts were also recruited after invitation from the NNR2023 Committee and from Chatham House. A fair distribution of experts among the Nordic and Baltic countries, in addition to experts with global competence, was sought when appointing experts. In addition, experts were recruited to bring a wide range of specialist knowledge from different research areas. While the input of the regional experts is highly appreciated, the final text in this paper is the sole responsibility of the authors.

Background information regarding food production and consumption in the eight focal countries is provided in Section 1, in addition to the aspects of environmental sustainability that apply across food groups and can therefore be considered as more general in application. Section 2 provides an overview of environmental sustainability considerations from a global and regional context for each NNR food group that we consider to be most significant in terms of consumption and environmental sustainability. Section 3 provides an overview of environmental sustainability considerations from a global and regional context for each NNR food group that has less overall environmental impact as a proportion of total diets compared to the focal food groups in Section 2, but which are still considered. Section 4 provides key considerations for the environmental sustainability of food consumption in Nordic and Baltic countries. Given the urgency of tackling a range of environmental issues, including climate change, we focus on options that have potential to reduce environmental impacts substantially in the immediate to short term, without reliance on supply side technologies currently under development, or not yet in widespread use. Hence, we focus our guidance on demand side shifts in consumption.

## Data sources for food availability, food consumption, and environmental impacts of food

The environmental analyses of food used in this paper represent the best available sources to demonstrate the impacts of food consumption in consistent units of analysis at the aggregate level across a range of food groups and across the Nordic and Baltic countries. As with all models that are analysing aggregate level data and impacts, the estimates provide an indication of impacts and a relative comparison between countries, foods, and food groups. They do not calculate the impacts of food production at the granular, detailed level within countries that is specific amounts of the numerous foods produced using a number of specific agricultural techniques. Hence, the environmental impact data presented in this paper do not constitute a national food system assessment that might consider the detailed impacts of food production and consumption.

All models for country-specific environmental footprint of foods, food groups, and food systems have specific data sources and handling methods. In Box 1, we describe data sources and handling that constitute the basis for the main environmental analysis and impact data used and cited in this paper.

## Section 1: Background context

### Food production in the Nordic and Baltic countries

All 8 countries use varying amounts of land area for agriculture. Denmark uses the largest proportion of land for agriculture (66%), followed by Lithuania (48%), Latvia (32%), Estonia (23%), Iceland (19%), Finland (7%), Sweden (7%), and Norway (3%).^3^ There are some large variations in terms of land used for agriculture across the 8 countries (Table 1). In all countries except Iceland and Norway, the largest proportion of agricultural land is used for temporary crops (defined by the FAO as crops with a less-than-one-year growing cycle, which must be newly sown or planted for further production after the harvest).

**Table 1:** Agricultural land use by country and type (2019) (27)^4^

	Cropland^5^ (1,000 ha)			Land under permanent meadows and pastures^6^ (1,000 ha)*
	Permanent crops^7^	Temporary crops^8^	Temporary meadows and pastures^9^	
Denmark	23.3	1794.4	524.6	206.7
Estonia	7.0	500.0	180.0	292.0
Finland	5.0	1237.0	786.0	24.0
Iceland	**	5.0	115.0	1751.0
Latvia	9.0	999.6	263.4	632.0
Lithuania	35.1	1886.4	258.5	728.0^10^
Norway	3.1	318.2	482.5	179.1^11^
Sweden	3.4	1320.7	1084.5	461.3

* Unless indicated by a footnote, land area under permanent meadows and pastures was not disaggregated into ‘cultivated’ and ‘natural growing’.

** Data missing or unavailable in the FAOSTAT database.

**Table 2:** Current food consumption (g/adult/day) per country and food group^12^

	Denmark	Estonia	Finland	Iceland	Latvia	Lithuania	Norway	Sweden	NNR2012 advice*
**Cereals#**	218		130	211	22,18	223	259	190	Depends on wholegrain content
**Bread**	138	71	92	81	3		184	87	
**Whole grains**	54			56			68	42	Increase (replace refined cereals)
Fruits and berries**	184	217	162	98	146	161	178	128	Increase
Vegetables**	199	145	184	114	216	185	155	176	Increase
Pulses***	1	6	13	5	17		4	12	Increase
**Potatoes**	92	94	74	72	105		67	88	-
**Fish and seafood#**	37	25	32	45	31	28	67	39	Increase
**Total meat#**	161	81	145	117	178	140	147	110	
**Red meat**	134	44	105	82	88	89	119	90	Limit
**Pig meat**	87		28	7		85			Limit
**Poultry**	27	19	39	35	49	49	28	22	-
**Dairy (without cheese)**	304	266	397	245	195	119	314	245	Replace with low fat types
**Cheese**	44	17	41	42	9	12	44	25	Replace with low fat types
**Eggs**	24	22	24	20	35	28	26	14	-
**Nuts$**	5	4	8	6	5		5	5	Increase

^1^ References for Table 2: Denmark: (34) 18–75 years, *n* = 3,016. Estonia: (35) 18–74 years, *n* = 2,713. Finland: (36) 25–74 years, *N* = 3,099. Iceland: (37) 18–80 years, *N* = 1,312. Latvia: Latvian National Dietary Survey (2007–2009), *N* = 1,377. Latvia: (38). Lithuania: (39) (2013–2014), *N* = 2,513.

Norway: (40) 18–70 years, *N* = 1,787. Sweden: (41) 18–80 years, *N* = 1,797.

* Qualitative advice from NNR 2012 (Table 1.1) (32).

** The food group ‘Fruits and berries’ does not include juice, while ‘Vegetables’ includes pulses, but excludes potatoes.

*** dry weight, # The food group Cereals contains both food ingredients and prepared foods such as bread (for Denmark also cakes and biscuits). Meat and fish are reported in raw weight, except sausages, cold cuts, and canned/smoked fish products. Total meat includes red meat and poultry – both uncooked and processed. Red meat and poultry in Estonia do not include processed meat. Red meat in Latvia does not include sausages. $ incl. seeds – if not included in bread.

Sweden has the largest area of farmland under organic production (613,964 ha), and Estonia has the largest share of total farmland under organic production (22%), followed by Sweden (20%), Latvia (15%), Finland (13%), Denmark (11%), Lithuania (8%), Norway (5%), and Iceland (0.4%) (28). Except for Iceland and Norway, all countries are part of the European Union, which has a goal as part of its farm to fork strategy for every member state to have a minimum of 25% of agricultural land under organic production methods by 2030 (29). Member states can go beyond this target.

In some countries, temporary pastures and meadows occupy much greater areas in comparison to permanent pastures and meadows, as shown in Table 1. As a comparison to the total area under permanent pastures and meadows, Denmark uses 2.5 times more land for temporary pastures and meadows, Finland uses 33 times more, Norway 2.7 times, and Sweden 2.4 times (Estonia, Iceland, Latvia, and Lithuania all use less land for temporary pastures and meadows compared to permanent pastures and meadows) (Table 1). In other analyses (i.e. not including the FAOSTAT used in Table 1), some of the land area used for food production in the Nordic and Baltic region is categorised as semi-natural grassland – this is usually an area of land cleared of native forest and maintained as low productivity grassland through farmed animal grazing on a proportion of native vegetation species and cultivated species. One estimate indicates that such areas tend to be much smaller than those used for permanent grazing, and also smaller than the areas used for temporary grazing. Sweden has the largest area of semi natural grassland, occupying an area equivalent to 10% of its total agricultural land – and potentially more than twice the area used for permanent grazing (30). However, the process of defining semi-natural habitats and assigning them to land use classes in national registers differs among the Nordic and Baltic countries (30), and as the FAOSTAT database does not include semi-natural pastures as categories of agricultural land, or natural growing permanent pastures and meadows for every country, it is not possible to obtain comparable land areas or food production outputs for such lands. Hence, we do not include semi natural grasslands as a category in our table of agricultural land (Table 1).

Additional food production and supply data (including imports and exports) from the FAOSTAT database in relation to each food group and country are provided in each major food group chapter, in as much detail as possible taking into account the limitations of data collection and availability (see Box 1 for more information on data sources and handling).

### Food consumption in the Nordic and Baltic countries

The mean daily consumption of selected food groups for adults derived from national dietary surveys in the Nordic and Baltic countries is presented in Table 2. The intakes differ between countries and also within countries, with large variances as described by Warensjö Lemming and Pitsi (31). Although differences between countries might be due to differences in the definitions of the food groups, and also that the years and methods of data collection differ (between 2007 and 2020), the countries share not only similarities but also differences in food preferences (31). The current Food Based Dietary Guidelines (FBDGs), which focus on population health outcomes, vary more or less between the countries when it comes to specific amounts or frequencies, but in general, all are partly based on the NNR 2012 (32). None of the Nordic countries are close to full adherence of their FBDGs. The surveys in different countries were conducted over a long period of time (2007–2022) and might not be accurate reflections of current intakes. The observed intakes may have been influenced by changing food markets (31). In Denmark, for example, intake of whole grains has increased considerably compared to the amounts shown in Table 2 (33).

#### Food consumption in relation to environmental sustainability targets

From an environmental sustainability perspective, the impacts of current diets in each of the Nordic and Baltic countries mostly exceed the levels that would be required to meet a range of global targets related to GHG emissions, cropland use, blue water use, nitrogen use, and phosphorus use (Fig. 3). A value above 1 (indicated by the red dashed line) exceeds the environmental target (or is consistent with the target being exceeded in future) and can be considered disproportionate in the context of an equitable distribution of environmental resources and mitigation efforts, and in effect, represents a country outsourcing its responsibility towards fulfilling the target (17),^14^ whereas a value or 1 or less is consistent with remaining inside planetary boundaries. For example, these calculations indicate that, if everyone in the world consumed the same average diet (indicated by ‘benchmark’) as currently consumed in Iceland, the food related component of the global GHG target (consistent with keeping warming below 2°C) would be exceeded by more than 5-fold. Shifting to current FBDGs would mostly improve the outcomes, but not enough. For example, if everyone in the world consumed the FBDG from 2013 for Denmark, the food related component of the global GHG target (consistent with keeping warming below 2°C) would still be exceeded by almost 4-fold (Fig. 3). Hence, not only are current consumption patterns largely incompatible with global environmental targets, but current FBDGs could also be improved to align with global environmental targets. It should be noted that some of the environmental impacts demonstrated in Fig. 3 occur in exporting countries, as the data represent consumption in the Nordic and Baltic countries.

Despite the varying approaches to assessing the environmental sustainability of current and future dietary patterns, the common conclusion is that environmental impacts generally decline as the amounts of animal products in the diet also decline – with vegan diets having the smallest environmental impacts, and diets highest in products from ruminant animals (cows, sheep, and goats) having the largest environmental impacts (2, 6, 14, 25, 43–51). This conclusion is also supported by systematic literature reviews (52–54) and original studies from the Nordic countries (55–58). Production locations and methods and food selection may add some nuance, for example, in terms of the importance of blue water use related to some imported vegetables, fruits, and nuts in some of the Nordic countries (59). The comparative approaches of the various dietary analyses of environmental impacts (i.e. Life Cycle Assessment, thresholds and systems) are described in the previous paper in this series and hence will not be described again here (13). In this paper, we focus exclusively on the environmental sustainability aspects of individual foods within food groups, and not dietary patterns. A combined assessment of nutritional aspects and environmental aspects of dietary patterns is considered in an upcoming paper of the series, where nutritional adequacy is assessed in the context of a whole diet rather than individual foods or food groups (60).

### General points across the food groups

A number of general, cross-cutting points apply across the foods and food groups and are therefore described in the following sections to avoid repetition in the food chapters.

#### Factors shaping environmental impacts

Environmental impacts are influenced by location and production methods, which can be determined by national contexts and policies (e.g. whether irrigated/rainfed, fertiliser types and amounts, plant protection types and amounts, land clearance, mono/poly cultures, crop rotations, manure management, tillage), processing (e.g. slaughter, cooking), packaging, and transportation (which can include refrigeration) type and distance (e.g. freight by road, rail, sea, or air). Hence, there can be significant variation in environmental impacts within the same food product depending on different practices and choices, primarily during production. For example, per unit of food production, due to their higher energy requirements, trawling fisheries and recirculating aquaculture can result in three times more GHGs than non-trawling fisheries and non-recirculating aquaculture; due to lower macronutrient densities and digestibility of feeds used in grass-fed systems, grass fed-beef requires more land and tends to emit more GHGs than grain-fed beef; and, in comparison to open field production, greenhouse production can have lower land requirements, higher yields, but almost three times higher GHGs (depending on heating and lighting usage and energy source) (61). However, the greatest differences in terms of environmental impacts tend to occur between foods and food groups, regardless of production practices (2). For example, in terms of GHGs, land use, energy use, acidification potential, and eutrophication potential, ruminant meats can have impacts 3–10 times higher than other animal-based foods and 20–100 times higher than plant-based foods on a per unit of weight basis (61).

The denominator used to express environmental impacts of a food can influence interpretation. For example, due to their high protein content, tree nuts, oils, pulses, rice, soybeans, and wheat are more efficient when measured by protein rather than energy, whereas cassava and sugarcane are more efficient by energy rather than protein content (1). While food waste is a major issue globally that must be addressed, it is not covered in this paper as it is assumed to be a known problem that applies across food types.

#### Technology

We are aware that there are numerous technologies designed to decrease the environmental impacts of agriculture, including livestock production. For example, several initiatives designed to reduce the environmental impacts of ruminant meat production are underway in the Nordic countries and elsewhere, including feed additives to reduce methane emissions and improved manure management. Technologies under development include cell culture and precision fermentation. For example, microbial proteins (62) could provide environmental benefits compared to animal-based proteins, and recombinant proteins synthesised by microbes could reduce environmental impacts compared to milk (63) and egg white proteins (64). Cell-culturing technologies can also be explored as a way to produce fats synthesised by microbes (65). While such technologies are all likely necessary to some extent, they are insufficient to deliver the scale and pace of transformation needed to meet planetary health goals – this necessitates changes in food consumption, and reducing food loss and waste (6, 14). Therefore, we concentrate our assessment on consumption shifts that are achievable in the short term [with appropriate reshaping of markets and political and public support (66)].

#### Supply side interventions

While the majority of environmental impacts of a food group or product often occur during the production (on farm) stage, there can be options for reducing impacts throughout the supply chain, including the processing and transport stages of the life cycle (67). For example, reducing GHGs from transport through improved loading logistics and reducing packaging amounts. In most food chapters, we demonstrate the variation in impact type and where it occurs along the supply chain for a range of foods. However, we assume that options for reducing the environmental impacts of production, processing, and transport (such as precision agriculture and other technological approaches) apply across the food groups and will therefore not necessarily be discussed in each individual food group. Existing technologies that are not currently implemented uniformly across food production include precision agriculture. Precision agriculture, a method used to apply inputs in a direct rather than diffuse way in appropriate amounts at the correct times, could be important for minimising inputs such as nitrogen and water use. The environmental benefits of increasing input efficiency through such technologies would be greatest in the least efficient systems (61).

Minimising environmental impacts in a relative way, for example, per unit of food produced, does not necessarily equate with environmental sustainability – an assessment will still be required to ensure the impacts of production are consistent with environmental goals. In addition, impacts will increase if relative impacts are reduced but absolute impacts increase due to an increase in production. Ultimately, while reducing environmental impacts throughout the food supply chain is necessary, this alone cannot achieve the absolute reductions required at the system level to align with environmental goals. Changing the types and quantities of food grown and consumed is also necessary (6).

#### Production methods

Using crop rotations and intercropping are considered important aspects of more environmentally sustainable food production, and directly overlap with sustainable food consumption in some cases. For example, combining grains and pulses during production and consumption is beneficial for farming practices and human health (67, 68). Conservation tillage and cover cropping, particularly with nitrogen-fixing crops, reduces the requirement for nitrogen inputs and increases fertiliser input efficiency by reducing nutrient loss from agricultural systems (61).

Organic food production can result in beneficial outcomes, including higher levels of associated biodiversity in comparison to non-organic production (69–72). For example, on-farm and near-farm biodiversity tends to be higher in organic agricultural systems, likely a result of lower fertiliser, herbicide, and pesticide use as well as through creating a more diverse landscape (73). In addition, the use of manure as fertiliser can promote higher soil organic carbon in organic systems. However, due to land use and land clearing requirements, applied on a large spatial scale organic agriculture would likely have a net negative impact on biodiversity and soil organic carbon (61). Therefore, while a shift to organic production at the global level could provide sufficient food availability, it is only feasible with a reduction in food waste and a shift to mostly plant-based diets to accommodate higher land use requirements [due to a reduction in yield, which is 34% lower when conventional and organic systems are most comparable (74)] without extending agricultural area (70).

Comparing the environmental impacts of conventional and organic food production at a local scale, a meta-analysis of Life Cycle Assessments (LCAs) found that, per unit of food produced, organic systems have higher land use requirements and eutrophication potential, lower energy use (due to less reliance on synthetic fertiliser, and pesticides), and no difference in GHGs and or acidification potential. The differences are largely a result of differences in nutrient management. Manure (which organic systems largely depend on for nitrogen) releases nutrients in response to environmental conditions rather than crop nutrient demand, which can reduce nutrient assimilation by plants, and lead to reduced growth and yields – in turn increasing land use requirements. Nutrients not taken up by plants can also result in eutrophication and acidification (61). However, there are techniques that can halve the land use difference between organic and conventional systems, including rotational farming, cover cropping, multi-cropping, and polyculture (61).

For some products, the difference in environmental impacts between organic and conventional production is relatively small, for example, GHGs from potatoes (75) and land use requirements for legumes and perennial crops (61). However, yield can be substantially lower in organic production – and can be further lowered if large portions of the harvest is lost or rejected (e.g. if it does not conform to certain size, appearance or shape standards, or due to higher vulnerability to pests and diseases). For example, organic banana plantations give around 50% lower yield compared to conventional production (75). Variety can also have an impact – for example, specialist varieties of tomato can have three times higher GHGs per kilogram of product than conventional varieties due to lower yields (75).

The solution might not be so simple as ‘organic or conventional’ or ‘pesticides or no pesticides’. Intercropping, agroforestry, and integrated pest management could play important roles – although the evidence for how this would operate in the Nordic region, with cold temperatures, needs to be further explored (76, 77). Crop rotations with brassicas to sanitise soil for cereals are considered important – but more knowledge about crops is needed, to identify which grow best together and which crop rotations are most successful – taking into account the practicalities for food producers that is establishing what can actually be grown. The choice between organic and non-organic also relates back to the choice of, or weighting of, metric. For example, organic farming has more benefits for biodiversity [e.g. underground biodiversity (78)] – but requires more land to produce the same amount; therefore, widespread uptake of lower-yielding, but nature-positive farming, requires commensurate changes in dietary composition (70). It is likely that a sustainable food system will need to integrate the benefits of conventional, organic, and other agricultural systems (61, 66).

#### Location and context

Due to a lack of comprehensive datasets on the environmental impacts of food production or consumption for each of the Nordic and Baltic countries for the range of foods covered in this paper, to demonstrate the environmental impacts of the foods within the NNR food groups, we used a global dataset of food LCA that has standardised boundaries to indicate a range of environmental impacts per unit of food production (2) (see Box 1 for more details on data usage).

The spatial distribution and concentration of different pressures related to food production vary on land and in aquatic environments. A recent analysis found that while global impacts of food production on habitat disturbance, GHGs, nutrient pollution, and freshwater use are dominated by land-based animal agriculture (with the greatest burden from pig meat closely followed by cattle meat), an estimated up to >10-fold variation in cumulative environmental efficiencies exist among countries for many livestock, fisheries, and crop products. For example, the efficiency of producing the same crops can vary 4–18 times among countries due to differences in water consumption, fertiliser/pesticide use, and farming practices. Similarly, efficiencies for marine fisheries vary up to 22-fold among countries depending on the specific species fished and equipment used within a country (1). Therefore, finer-scale analyses are useful to identify where environmental pressures are located and how the environmental efficiency of production might vary among regions. Spatial cumulative footprint assessments explore where and how much each type of food contributes to food’s total environmental footprint. The impacts depend on what is being displaced (e.g. forest, wetlands), the sensitivity of systems to specific pressures, and local biophysical and socioeconomic conditions (1). For example, if food production is spatially located in areas with the most suitable climatic and soil conditions for a crop, this can increase agricultural input efficiency and decrease environmental impacts but may have implications for how far food is transported (61).

Another issue related to location and context is the use of agricultural wastes and by-products as animal feeds, which has the potential to reduce the environmental impacts of livestock production by 20% (61) – but is dependent on food safety/environmental regulations in each country or location.

#### Biodiversity impacts

Generally, biodiversity can be considered as a broad concept that includes diversity at the genetic, species, and ecosystem level, and is an essential component of functional ecosystems. The available comparative data for LCA used in this paper does not incorporate metrics for the impact of production on biodiversity, partly because different studies are highly heterogeneous in what they assess as ‘biodiversity’, and partly because of very significant context- and scale-dependency. For the former, different taxonomic groups (birds, plants, insects, and microbes) may respond very differently to field-, farm-, and landscape drivers. For the latter, there are significant geographic and climatic effects that constrain a farm’s biodiversity, which interact with the nature of the surrounding landscape to determine what may be found there and how agricultural practices may impact (3, 66). Biodiversity impacts are therefore difficult to measure and indicators and metrics largely rely on value judgements (e.g. depending on which species or landscapes are considered of most value to humans), and the availability of a baseline comparison. The decisions around conservation or protection of certain species could differ depending on the values of the decision maker/s.

Farming impacts biodiversity in different ways and is very complex to assess at the system level and more so at the product level, making comparisons with other environmental metrics difficult. Monocultures reduce biodiversity both in terms of crop types, and by limiting the types of habitats and food availability needed to support a diverse range of wildlife (3). Areas where the same crops are grown every year lack the beneficial decontaminating effects of crop rotations, resulting in an increased requirement for plant protection products. Monocultures at a landscape level can have a range of impacts, including on the fauna and flora (73). For some crops, genetic diversity within the crop itself is important for reducing disease, for example, potatoes (75). Producing food with less pesticide use would be positive for biodiversity. Since the 1950s, pesticide use has had major impacts on soil and above ground biodiversity. Across Europe, pesticide and fertiliser use has been identified as the main pressure for bird population declines across the vast majority of common birds – especially invertebrate feeders but also farmland species, long-distance migrants, and woodland birds (79). Taking a cautious approach and applying pesticides and fertilisers only when absolutely necessary could help – via precision agriculture methods, for example. However, to maximise biodiversity gains, it is important to provide the necessary space and habitat for wildlife in the landscape – reducing the use of pesticides and fertilisers alone will not be sufficient (3).

That biodiversity is not covered in the LCA data presented in the food groups is not to detract from its local and global importance. Biodiversity underpins a wide range of supporting and provisioning ecosystem services (from soil fertility, carbon storage, pollination, and natural pest control) as well as having cultural and social value. Across Europe – and globally – wildlife has been in significant decline for decades, and the bulk of this is caused by agriculture interacting increasingly with climate change (80). Reducing the demand for, and pressure on, land through changing the composition of diets may allow more environmentally beneficial farming systems to be adopted, and help to protect and restore globally important carbon and biodiversity repositories (3, 4, 67, 81). The Kunming-Montreal Global Biodiversity Framework (82) was adopted in December 2022 (including by all eight Nordic and Baltic countries). The framework contains 23 targets for 2030, a number of which have significant implications for food production and consumption and could thus raise the political importance of such issues in the coming years.

## Section 2: Focal food groups

This section contains a chapter for each of the following NNR food groups: cereals; vegetables, fruits, and berries; pulses (legumes), fish, fish products and seafood, meat and meat products, milk and dairy products, and eggs.

## NNR Food Group 3: Cereals

### Global context

Globally, maize is the type of cereal produced in the largest quantity (1,162 million tonnes (mt)), followed by wheat (761 mt), rice (757 mt), barley (157 mt), oats (25 mt), and rye (15 mt) (83). In terms of use, cereals are a major source of livestock feed. Currently, 42% of global cereal production is dedicated to feeding farmed animals, which alone uses 43% of global cropland (2). As a proportion of global production, animal feed currently accounts for 17% of wheat, 4% of rice, 56% of barley, 61% of oats, 46% of rye, and 59% of maize production (84). It should be noted that the ratio of cereals allocated to farmed animal feed versus direct human food is not uniform each year – variations can occur due to, for example, weather conditions and fertiliser prices.

Across the impacts shown in Fig. 4, most impacts for cereals occur during production that is on farm. Maize has the most land-use change associated with production, followed by wheat and grains.^15^ All impacts from losses are very similar across the cereals.

Under business-as-usual food consumption and expected population growth to 2050, the absolute environmental impacts of cereal production will increase, even if the efficiency per unit of production improves. For example, it has been estimated that a further two- to three-fold increase in nitrogen (N) supply will be required to support global food production for the anticipated population of ~9.7 billion by mid-century. Synthetic N applied to cereal production (wheat, rice, and maize) accounts for more than 50% of the total fertiliser used globally for crops. Three countries consume more than half of this amount across the three cereals: China (22.7%), India (18.5%), and the US (14%) (85). However, there is substantial scope to reduce N use, given that only 35% of the 115 million tonnes of N applied annually to crops is actually taken up by them (the remaining 75 million tonnes of N, or ‘excess N’ discharges into the surrounding environment including waterways and atmosphere). Excess N varies significantly by region, with China having the largest share (33% excess N) of the global total, followed by India (18%) and the US (11%) (86, 87). Applying N in the correct quantities, at the correct time and in the correct place is an important method for reducing excess N. Such precision agriculture techniques can be assisted with the use of technology, and also require training and knowledge among farmers.

In terms of individual crops/products, maize meal tends to have the lowest environmental impacts across the cereals shown in Table 3, particularly in relation to freshwater use. Paddy rice has the highest GHG footprint, which is primarily a result of methane emissions from waterlogged rice fields during the production stage. The majority (80%) of global rice production is cultivated in waterlogged fields, which are either irrigated or rainfed. While rice can be grown in dry conditions, the yield is much lower in comparison (around 33% of the yield from paddy rice) (75, 88).

**Table 3:** Average environmental impacts per kg of retail weight: Cereals (global) (2)

	Land use (m^2^/kg)	Greenhouse gas emissions (kg CO_2_eq/kg)	Acidifying emissions (g SO_2_eq/kg)	Eutrophying emissions (g PO_4_^3-^eq/kg)	Freshwater (L/kg)
Wheat & Rye (bread)	3.9	1.6	13.4	7.2	648
Maize (meal)	2.9	1.7	11.7	4.0	216
Oatmeal	7.6	2.5	10.7	11.2	482
Rice	2.8	4.5	27.2	35.1	2,248

Substituting one cereal for another with lower environmental impacts could be an option for improving environmental sustainability outcomes. However, the intended use is important to consider. For example, where high protein and/or gluten content is required (e.g. wheat for bread making and pasta), this characteristic would need to be matched. In terms of environmental impact, high protein content tends to be associated with high application of N fertiliser (75) – although not necessarily if grown with legumes due to their nitrogen fixing properties (67, 68).

Thus, it might be more appropriate to consider alternative food products rather than seek like-for-like replacements. Another potential option is to grow cereals in rotations with legumes to benefit from their biological N fixation and in turn reduce reliance on synthetic N fertiliser (i.e. obtained through the Haber-Bosch process) (85).

An expansion of cereal production is not inevitable, even taking into account the expected growth in the human population. The major route for reducing all environmental impacts of cereals is in conjunction with a shift to more plant-based diets (particularly in high- and middle-income countries), thus reducing the amount of cereals required for farmed animal feed. The demand for animal feed is partly a result of high levels of livestock production, but largely due to the inefficiency of converting crop nutrients to animal products. For example, an analysis based on US production estimated that to deliver 1 calorie of beef for human consumption requires 37 calories of plants, 1 calorie of pork requires 12 calories of plants, 1 calorie of chicken requires 9 plant calories, 1 calorie of eggs and 1 calorie of dairy each require 6 plant calories (89). Global averages for protein feed conversion efficiency (i.e. the % of protein in feed converted to protein in product) range from 4% for beef to 25% for eggs. The equivalent range for calories (i.e. the % of energy in feed converted to energy in product) is 2% for beef and 24% for whole milk (90). At the global level, 36% all calories produced from crops are fed to farmed animals with only 12% of those calories returning as livestock products for human consumption, such as meat and milk (91) – equating to a loss of 32% of all crop calories produced due to this conversion process (92). The world’s croplands could potentially feed billions more people by shifting from animal feed to producing food for human consumption (87). It would also be important to reconfigure crop production, to enable human edible crops to be grown in place of animal feed crops. It has been estimated that by taking this approach in the US (i.e. replacing feed crops with healthy alternatives for human consumption, such as legumes, whole grains, fruits, and vegetables) could feed an additional 350 million people without increasing the cropland area (93).

### Nordic and Baltic context

Figure 5 shows the variation in terms of impacts of cereal consumption across the Nordic and Baltic countries and how they relate to global environmental limits for food consumption. For example, if everyone in the world ate the same amount of cereals as Lithuania it would use over 70% of the global nitrogen amount allocated to food consumption.

Denmark is the biggest grain producer of the Nordic and Baltic countries, followed by Sweden and Lithuania (Table 4). Grain production is sufficiently high to satisfy national consumption in every country, except Iceland and Norway, where there is some reliance (a strong reliance for Iceland) on imports to meet demand. Lithuania and Latvia are the largest exporters of cereals, followed by Denmark. It should be noted that production amounts and import demand is not uniform each year – instead, annual variations can occur.

**Table 4:** Cereal production and supply across the Nordic and Baltic countries in 2019 (84)

Country	Production (1,000 tonnes)	Imports (1,000 tonnes)	Exports (1,000 tonnes)	Balance* (1,000 tonnes)	Food supply (Kg/person/year)
**Denmark**	9,518	1,348	1,822	9,044	118
**Estonia**	1,630	165	1,162	633	96
**Finland**	4,036	327	716	3,647	119
**Iceland**	8	103	0	111	80
**Latvia**	3,163	861	3,223	801	115
**Lithuania**	5,252	796	4,298	1,750	147
**Norway**	1,324	885	41	2,168	124
**Sweden**	6,148	1,087	1,552	5,683	110

* The amount remaining for national use taking into account production, imports and exports (balance = production + imports – exports).

Lithuania is the biggest wheat producer, followed by Denmark and Sweden. Denmark is the biggest producer of rye, followed by Sweden and Latvia. Finland is the biggest producer of oats, followed by Sweden and Denmark. Denmark is the biggest producer of barley, followed by Sweden and Finland. Lithuania is the biggest producer of maize, followed by Denmark and Sweden. None of the countries currently produce rice {FAO, 2020 #64}. Globally, Finland and Sweden are within the top 10 largest oat producers (4th and 10th respectively), and Denmark is the 4th largest producer of rye.

In every country except Estonia, Iceland, and Sweden – more than half of cereal supply is used to feed farmed animals, and in every country animal feed accounts for a substantial share of cereal supply. Allocation is highest in Denmark which uses 75% of cereal supply for farmed animal feed, followed by Latvia (63%) and Finland (59%) (Table 5).

**Table 5:** Cereal allocation to animal feed and direct human food across the Nordic and Baltic countries in 2019 (84)

	Feed (1,000 tonnes)	Food (1,000 tonnes)	Feed as % of total supply^16^
**Denmark**	6,791	683	75
**Estonia**	315	127	50
**Finland**	2,149	659	59
**Iceland**	44	27	40
**Latvia**	505	219	63
**Lithuania**	963	405	55
**Norway**	1,103	670	51
**Sweden**	2,662	1,102	47

Given that animal feed occupies a large proportion of supply in every country coupled with the general need to reduce livestock production and consumption of animal products, there is scope to explore reducing overall cereal supply and/or reallocating supply from animal feed to human food for direct consumption. Depending to some extent on local conditions and cropping systems, it might be possible for all countries to shift supply of cereals from feed to food. Table 6 describes the relationship between cereals/grains used as feed and that used as human food. The larger the number the larger the proportion of cereals used for feed compared to use for food. For example, in Lithuania 114 times more oats are used for farmed animal feed compared to human food. The feed used to food supply ratios shown in Table 6 could guide the exploration, taking the size of the cereal food supply in Table 7 and the production in Table 4 into account. The option to shift cereal supply to human food for direct consumption would depend on the quality/grade of the cereal. Where the grade is not high enough for human consumption, there could be potential to increase the grade during production with additional resources, that might include skills and training for cereal producers.

**Table 6:** Feed to food supply ratio across the Nordic and Baltic countries in 2019^17^

	Wheat	Rye	Barley	Rice	Oats	Maize	Cereals, other
**Denmark**	6.8	8.5	-	0.2	8.8	10.6	61.0
**Estonia**	3.0	0.5	16.1	0.0	3.2	1.0	27.0
**Finland**	0.7	0.0	21.6	0.0	36.6	-	1.9
**Iceland**	0.8	-	4.9	0.0	0.0	-	-
**Latvia**	1.5	0.8	4.1	0.0	15.6	14.5	2.6
**Lithuania**	0.5	1.5	-	0.1	114.0	101.0	49.6
**Norway**	0.4	2.7	38.7	0.0	3.5	-	-
**Sweden**	1.0	0.0	-	0.0	21.4	3.2	71.3

- Data unavailable to make the calculation.

**Table 7:** Cereal food supply (not including cereals produced for feed) across the Nordic and Baltic countries in 2019 (kg/person/year) by cereal type

	Wheat	Rye	Barley	Rice	Oats	Maize	Cereals, other
**Denmark**	85	18	0	5	4	5	0
**Estonia**	55	16	5	3	5	10	3
**Finland**	79	26	5	5	3		1
**Iceland**	71	1		4	3		0
**Latvia**	62	25	18	3	3	1	4
**Lithuania**	113	15	14	3	0	1	1
**Norway**	102	3	3	5	12	0	0
**Sweden**	87	10	2	6	2	2	0

Wheat is by far the main grain consumed in each country, followed by rye – except for Iceland where more rice and oats are consumed in comparison to rye, and in Norway where more oats and rice are consumed in comparison to rye. In comparison to wheat and rye, there is generally a relatively low consumption of oats and barley across the countries (Table 7). Hence, there could be potential to increase the consumption of oats and barley in most if not all countries. Increasing/shifting to barley consumption could be a way to improve the environmental sustainability of cereals given that barley generally has the lowest environmental impacts across a range of metrics (substantially lower for some metrics, e.g. freshwater use) (Table 3). While the current availability of freshwater across the 8 countries is generally not a limiting factor for food production, it is possible that some recently observed conditions (e.g. drought) could intensify in frequency and magnitude in the future due to the impacts of climate change. Also, the subsequent impacts (e.g. low availability of hydro power and high export leading to high energy prices) could increasingly impact food production. Shifting a portion of oats and barley currently used for animal feed to human food for direct consumption could particularly benefit Iceland which is currently dependent on cereal imports to meet supply (Table 4), in turn helping to increase consumption of such cereals and potentially reducing import dependency.

Exploration of new crop options/reintroductions such as quinoa and millet could emerge as local growing conditions change and opportunities to cultivate different crops arise. For example, quinoa is now grown in Finland, quinoa and chickpeas are now grown in Denmark, and there is potential to resurrect millet production. There is scope to learn from similarly positioned countries that have experience growing such crops on a commercial scale (e.g. the UK {BBC News, 2014 #207}). Breeding of locally adapted varieties and development of locally applicable cultivation practices are also needed to support the production.

#### Considerations for reducing environmental impacts of cereal consumption in Nordic and Baltic countries

From the overview of cereal production, consumption, and trade provided in this section, we identify a number of opportunities to explore as potential ways to reduce environmental impacts of the cereal food group:

- **Opportunities with major benefits:**
  - Cereals have among the lowest relative impacts, from an environmental sustainability perspective, which makes increased consumption an option for increasing environmental sustainability of diets. Increased consumption of whole grains is in general recommended for humans based on health evidence, including for nutrient supply such as fibre (32, 94).
  - Shifting portions of animal feed to direct human food if possible (differences in cereal quality need to be determined and adjusted if needed, and crop reconfiguration to best support human health might be required) would allow cereal/grain consumption to increase, without increasing production (and the associated environmental impacts) and would need to be supported by a reduction in animal agriculture (see chapter 8).
  - Increase the proportion of cereals grown using less environmentally damaging methods (assisted by an overall reduction in cereals grown for animal feed). This could include organic methods (or similar), and increasing and improving crop rotations with legumes to reduce the requirement for synthetic N (20). Precision agriculture techniques could further reduce ‘excess nitrogen’ and other key resource requirements, including water.

**Opportunities with relatively minor benefits:**

- Explore new crop options and reintroductions (such as millet). As local growing conditions change, more opportunities to cultivate different crops might arise.
- Explore differences in environmental impacts from locally (i.e. within each country), regionally (i.e. within the eight countries) and internationally sourced cereals to potentially identify further options for reducing environmental impacts, taking into account potential changes in environmental conditions and increasing occurrence of environmental shocks. Environmental impacts of imported cereals might vary depending on the types and amounts of pesticides and fertilisers that are permitted in the country of origin, the methods of production such as intensive farming with tilling versus no till and organic (or similar) methods, and the conditions in the country of origin such as water shortages and soil degradation. Trade stipulations might be a useful tool for reducing the environmental impacts of imports.

## NNR Food Group 4: Vegetables, fruits, and berries

### Global context

At the global level, in terms of fruit, bananas are currently produced in the largest quantity (118 mt), followed by oranges (114 mt) and apples (87 mt). In terms of vegetables, potatoes (which are considered a separate food group in the NNR context) are produced in the largest amount (370 mt), followed by casava (300 mt) and tomatoes (180 mt) (84). Approximately 10% of global potato production and 30% of cassava production are used as farmed animal feed (84).

Figure 6 demonstrates the proportion of environmental impacts associated with different fruits and vegetables throughout their product life cycles. For some products, the production stage accounts for the majority of impacts, for example, brassicas and cultivated berries, while for other products and impacts, other stages are more important, for example, transport of bananas in relation to GHGs. Citrus production has the largest carbon sequestration benefits (through tree growth) per unit of product, followed by apples and bananas.

Root vegetables (including potatoes) and onions generally have the lowest environmental impacts per unit of weight, particularly in comparison with salad vegetables such as tomatoes. Root vegetables (including potatoes) and onions can be most easily stored, with relatively small inputs and little waste. In terms of fruits, apples and citrus tend to have the lowest associated environmental impacts, and also store well (Table 8).

**Table 8:** Average environmental impacts per kg of retail weight: Vegetables, fruits, and berries (global) (2)

	Land use (m^2^/kg)	Greenhouse gas emissions (kg CO_2_eq/kg)	Acidifying emissions (g SO_2_eq/kg)	Eutrophying emissions (g PO_4_^3-^eq/kg)	Freshwater (L/kg)
**Potatoes**	0.9	0.5	3.9	3.5	59
**Cassava**	1.8	1.3	3.4	0.7	0
**Tomatoes**	0.8	2.1	17.2	7.5	370
**Onions & leeks**	0.4	0.5	3.6	3.2	14
**Root vegetables**	0.3	0.4	2.9	1.6	28
**Brassicas**	0.6	0.5	8.2	5.0	119
**Citrus fruit**	0.9	0.4	4.0	2.2	83
**Bananas**	1.9	0.9	6.4	3.3	115
**Apples**	0.6	0.4	3.5	1.5	180
**Berries & grapes**	2.4	1.5	12.3	6.1	420

There are a range of factors that influence the environmental impacts of fruits and vegetables. Production practices can vary highly within products and even within countries (e.g. use of pesticides and fungicides varies significantly even across EU countries). The scale of production (from small holder to industrial level) can have a significant influence. Generally, larger scale production has higher yields and more efficient production, which can be used as a metric of environmental impact per unit of production (but not on biodiversity). However, that is not always the case and is potentially short-term if, for example, soil degradation prevents the maintenance of such yield levels. For example, highly mechanised production, such as in orange groves, can increase fuel use and GHGs, and a high application of synthetic fertiliser can result in pollution of nearby waterways.

Some crops are typically produced under a certain method. For example, bananas, citrus, and tomatoes are largely produced in intensive monoculture plantations for the export market, which have a range of associated issues such as use of herbicides to remove other vegetation from the land (leading to soil erosion and run-off), and larger requirements for plant protection products (such as chemical pesticides) and fertiliser. Potato production tends to involve more intensive soil tilling compared with other crops, which releases GHGs and reduces soil organic matter content, making organic fertilisers particularly important for crop rotations involving potatoes (75).

In general, more chemical plant protection products are used in the production of fruits and vegetables than other types of agricultural production (in terms of per hectare and kg of harvested product), and tend to be higher in intensive fruit and berry production compared with vegetables (75). While pesticide use is mostly concentrated during the production stage, for some fruits it is also applied at other stages, for example, fungicides applied to bananas for transportation, and some are applied to the soil, for example, soil disinfectant for strawberries to prevent mould. Production in warmer locations can also require higher levels of pesticide application. Some crops require plant protection to enable sufficient yields, for example, fungicides are applied to control potato blight (75). Hence, yields from organic potato production are significantly lower – potentially up to 50%. The use of climate-controlled greenhouses can reduce pesticide requirements, as can crop rotations (which can also help reduce the occurrence of potato blight) (75).

In comparison to outdoor production, greenhouses can produce greater quantities of food on a smaller area over a shorter time period. Food production in greenhouses can also reduce the reliance on having land available with appropriate soil quality, which is a limiting factor in some locations, for example, Norway, and can be more efficient with respect to water use. There can be substantial differences due to production methods, even with greenhouse production. For example, GHGs from organically produced tomatoes can be 40% higher due to a reduced yield in comparison to conventional production (everything else being equal in terms of energy to heat and light the greenhouse) (75). A major trade off compared with outdoor production is the energy requirements for heating (and lighting and sometimes cooling) the greenhouse and the subsequent GHGs (95), versus a greater requirement for plant protection products outdoors. However, this trade off could be reduced if renewable energy sources or waste heat are used to power the greenhouse – or with greater energy efficiency – or if crops with lower demands for heating/cooling were cultivated. In addition, food production in greenhouses generally requires less resource input in the form of fertiliser and pesticides compared to outdoor production (96) and can protect crops to a certain degree from climate extremes, thereby reducing crop losses in extreme weather events.

The impacts related to harvesting, processing, and losses incurred during the life cycle stages can also vary substantially. For example, higher levels of waste result from consuming orange juice compared to whole oranges. The types of equipment used during harvesting can also be important – for example, energy use in apple production tends to be highest where hydraulic ladders/platforms are used. Minimising losses during storage is particularly important (97).

While the environmental impacts of transport tend to account for the smallest portions of overall impact, the proportion of overall impact can be more significant for fruits and vegetables compared to animal products, for example. In general, transport via rail and sea is less impactful compared to air and road freight; however, the actual impacts of all types of food also depend on travel distance, load, and requirements for refrigeration. Seasonal consumption of locally produced fruits and vegetables can help to reduce the requirements for longer distance transport of fruits and vegetables with a short shelf life, and/or that require refrigeration. For some crops, it is possible to extend the season through selection of multiple varieties, for example, strawberries that fruit at different times, in turn reducing import requirements. Conversely, where growing conditions are particularly challenging, importing fruits and vegetables such as tomatoes, lettuces, and cucumbers from warmer regions could be the lower-impact option in terms of GHG emissions, for example, if the full life cycle is considered (i.e. not just comparing the transport impacts). For other impacts, such as water and pesticide use, production in the Nordic/Baltic countries could be more sustainable than imported products.

### Nordic and Baltic context

Figure 7 shows the variation in terms of impacts of fruit and vegetable consumption across the Nordic and Baltic countries and how they relate to global limits for food consumption. For example, if every country in the world consumed the same as Estonia, it would use 30% of the global limit in terms of nitrogen allocated for food consumption. Given that the consumption of this food group needs to increase across the Nordic region, reducing the environmental impacts of fruits and vegetables would be beneficial, particularly in relation to nitrogen use and pesticides.

Generally, there is significant potential to increase fruit and vegetable production across the Nordic region (including Iceland). In terms of fruit – the production of apples, pears, cherries, currants, and plums could potentially be increased. Strawberries, raspberries, bush blueberries, buckthorn, and other berries grow well in the local climate. The potential to harvest more wild berries might be substantial (most wild berries in Norway, Finland, and Sweden (98) are unpicked, e.g.). However, there are no data on the amount of wild berries that are accessible and could be harvested sustainably, the potential consumer demand for wild berries, or the potential impact on wildlife that might utilise the berries as a food source. Incentivising the harvest of wild berries could also be difficult (some countries such as Finland currently rely on seasonal labour from migrant workers) – but there is potential to link this with health and recreation activities rather than having a sole purpose of food harvesting. Wild herbs and plants such as nettles might also have some potential within the region. Root vegetables, including potatoes and brassicas could be increased. The main limitations across the region are availability of arable land, a short growing season and the need for a long storage period (with methods that reduce losses). Despite this, there are estimates to suggest that in Norway, under crop rotations and optimal use of agricultural soils, vegetable production could be increased by around five times and potatoes could be increased by around seven compared to current production levels (99).

Increasing climate-controlled greenhouse production could reduce the requirement for plant protection, and help to overcome limits of outdoor fruit production across the region (96). However, energy use could become an issue but this could evolve if the input from renewable energy sources increases and the energy efficiency of climate-controlled GHG production improves, making greenhouse production more viable (although reducing energy demand overall rather than replacing all fossil fuel energy with renewable sources should be a key step). Energy and food prices have been identified as important variables in the Norwegian context, and the provision of government support (investment, electricity costs), that is not unlike other aspects of government support (e.g. import protections; subsidised livestock production) to enable adoption (100–103). Iceland already uniquely benefits from substantial climate-controlled greenhouse production, facilitated by geothermal heat and hydropower (104).

Identifying the extent to which increasing crop diversity and agroforestry can reduce pesticide use and improve other environmental sustainability outcomes requires more study across the region. Based on a number of trial farms in Sweden, it could be viable to implement agroforestry to grow nuts, berries, and fruits in agricultural systems (105).

There is a good potential for rain-fed agriculture in the Nordic and Baltic region, with some possible requirement for irrigation during the summer season. While there are currently no major water stress issues in the Nordic and Baltic countries, there are several other considerations regarding increasing fruit and vegetable production. An increase in labour would be required, particularly for methods such as spatial intercropping on a small scale. Proximity to markets and/or processing facilities could be important for some crops, and profitability for farmers could be an issue. Competition with cheaper imported products, lack of agreement between producers and retailers, and toll barriers are also an issue – for example, domestic production needs to compete with imported products. The variation in trade support could therefore influence crop types. Also identified as important in the Nordic context is the preservation of certain types of food (from a normative or cultural perspective), which could play a role in influencing the viability of different crop types. There are some important locational perspectives, such as the policies to take peatland out of agricultural production in Denmark, Norway, and Finland. Although policy effectiveness is in turn a large determinant of whether such locational considerations impact food production – for example, a law to prevent peatland conversion in Norway has been found to be largely ineffective (106).

Currently, all Nordic and Baltic countries have a substantial dependency on imports to meet domestic demand for fruits and vegetables. For some countries, such as Iceland, a high dependency has been placed on air freight. Increasing consumption of vegetables and fruits is necessary to obtain healthy and sustainable plant-rich diets, and this requires either an increase in local production, a change in consumption (i.e. to eat more seasonally available products), an increase in imports, or a combination of those factors. There could be a cultural expectation among consumers to continue importing food in large proportions to maintain purchasing possibilities (such as strawberry availability during the winter), and to see a continuation of current land use nationally (such as high levels of forest cover), rather than more conversion to agriculture. One possible factor that might help to maintain such expectations is that the environmental impacts of imports are not seen or experienced directly by consumers in importing countries. However, this should be contrasted against concerns regarding preparedness for trade disruptions and resilience to environmental shocks. Some level of domestic food security considerations should be integrated into food system planning, to avoid an expectation among rich nations that they will always be able to ‘buy themselves out of food supply problems’. Such considerations could also be extended to imported supplies – for example, decreasing imports from water-scarce regions (e.g. in Spain) and regions that are likely to become water stressed.

Given the various trade-offs and variation in impacts, identifying which crops or production methods or locations are ‘best’ for environmental sustainability depends on the metric or metrics given most weight – which might be determined, for example, by a pressing environmental issue such as drought, or a value judgement. There is a lack of knowledge about the production systems and their environmental impacts for imported fruits and vegetables, which further complicates the process. Including issues, such as social impacts, in addition to environmental sustainability could also change the weighting/priority order. Furthermore, considering production systems over the long term is also important – for future generations and to account for environmental changes. For example, climate change is expected to shift pest and disease ranges suggesting that future farming systems, and crop suitability, will change in the Nordic region. More frequent periods of drought could enhance water pollution and create water scarcity issues. Therefore, a focus on groups of fruits and vegetables that are more resilient to climate and environmental shocks, and store well (such as potatoes and apples) could be most appropriate.

#### Fruits and berries

All countries have a substantial dependency on imports to meet domestic fruit and berry supply. Lithuania is the least dependent on imports, with a 4-fold ratio of production compared to balance (taking into account imports and exports) (Table 9).

**Table 9:** Fruit and berry production and supply across the Nordic and Baltic countries in 2019 (84)

Country	Production (1,000 tonnes)	Imports (1,000 tonnes)	Exports (1,000 tonnes)	Balance (1,000 tonnes)	Food supply (Kg/person/year)
**Denmark**	47	546	157	436	60
**Estonia**	4	103	5	102	75
**Finland**	31	420	14	437	73
**Iceland**	0	31	0	31	87
**Latvia**	17	184	76	125	49
**Lithuania**	42	345	213	174	47
**Norway**	33	397	2	428	77
**Sweden**	41	824	91	774	59

For all countries, other fruits (a group which includes pears, quinces, apricots, cherries, peaches, plums, strawberries, raspberries, gooseberries, currants, blueberries, melons, figs, and mangos) are the most highly consumed. Except for Estonia, this is followed by oranges, bananas, and apples, which are the most consumed single fruit in Estonia (Table 10).

**Table 10:** Fruit and berry food supply across the Nordic and Baltic countries in 2019 (kg/person/year) (84) by fruit type

	Other fruits	Bananas	Oranges	Apples	Grapes	Pineapple
**Denmark**	22	11	10	3	7	3
**Estonia**	21	14	15	16	5	2
**Finland**	21	18	16	9	4	3
**Iceland**	28	9	14	13	5	3
**Latvia**	17	11	9	4	2	1
**Lithuania**	15	8	11	6	2	1
**Norway**	22	15	17	13	5	2
**Sweden**	21	7	16	7	3	2

#### Vegetables

All countries have a substantial dependency on imports to meet domestic vegetable supplies. Lithuania is the least dependent on imports, with a 1.6-fold ratio of production compared to balance (taking into account imports and exports) (Table 11).

**Table 11:** Vegetable production and supply across the Nordic and Baltic countries in 2019 (84)^19^

Country	Production (1,000 tonnes)	Imports (1,000 tonnes)	Exports (1,000 tonnes)	Balance (1,000 tonnes)	Food supply (Kg/person/year)
**Denmark**	261	420	79	602	96
**Estonia**	25	97	9	113	81
**Finland**	263	253	8	508	85
**Iceland**	5	23	0	28	77
**Latvia**	62	175	52	185	89
**Lithuania**	178	232	116	294	96
**Norway**	195	244	1	438	75
**Sweden**	310	699	55	954	84

Except for Latvia and Lithuania, other vegetables (a group which includes cabbages and other brassicas, artichokes, asparagus, lettuce, spinach, pumpkins, peppers, carrots, mushrooms and frozen, dried, and preserved vegetables), are the most highly consumed across all countries, followed by potatoes (which are the most consumed vegetable in Latvia and Lithuania) (Table 12). Consumption across the countries is generally focused on very few products (e.g. cucumber and salad vegetables); hence, there is scope to expand the diversity of vegetable consumption.

**Table 12:** Vegetable food supply across the Nordic and Baltic countries in 2019 (kg/person/year)^20^ by vegetable type

	Other vegetables	Tomatoes	Onions	Potatoes
**Denmark**	76	14	7	61
**Estonia**	62	13	7	57
**Finland**	62	15	8	58
**Iceland**	59	12	6	39
**Latvia**	69	12	8	112
**Lithuania**	69	18	9	84
**Norway**	62	10	3	49
**Sweden**	61	16	8	55

#### Considerations for reducing environmental impacts of fruit, berry, and vegetable consumption in Nordic and Baltic countries

Even though vegetables, fruits, and berries have among the lowest relative impacts, from an environmental sustainability perspective, based on the overview of production, consumption, and trade, we identify a number of opportunities to explore as potential ways to reduce environmental impacts of the fruit, berry, and vegetable food groups. As consumption of this food group would need to increase to meet dietary guidelines (Table 2), the opportunities relate to reducing environmental impacts per unit of production, or relative impacts, rather than reducing total, or absolute, impacts from this food group. Our suggestions span across production and consumption aspects and could have relatively major or minor impacts depending on the level of uptake/implementation:

- Diversifying consumption of fruits and vegetables could reduce the dependency of imported salad vegetables during the winter months and import of fruits during the summer months. This would include a decreased consumption of tomato, cucumber, pepper, and lettuce during the winter and restricting consumption of these products more to the summer and autumn. During the colder months (winter and spring), a greater consumption of root vegetables (e.g. carrot, parsnip, celeriac, swede, and beetroot), brassicas (e.g. cabbage) and onions could potentially reduce the need for salad vegetables, in addition to using vegetables that preserve well to enable longer storage and use. Even where imports of vegetables are necessary during the winter months, importing Chinese cabbage, onions, and root vegetables has a lower GHG impact than importing salad vegetables, due to increased storability and reduced risk of waste during transport and post-retail. In terms of fruit, this could involve an increase in tree fruit (e.g. apples, pears, cherries, and plums) during the colder months and wild berries during the summer months. However, the potential impacts on local wildlife that rely on wild berry consumption should be considered. Estimated harvest levels are low across the region (107–109). However, the estimated harvest levels are variable and uncertain, and if berry utilisation should increase drastically, the impacts on biodiversity may need to be assessed.
- Increasing the supply of tree fruits (such as apples and pears) produced within the region could help reduce the import of citrus, bananas, and grapes. Increasing the domestic cultivation of a wider variety of apples would also help diversify the agricultural landscape, and extend the season due to variations in harvesting, ripening, and storage times (which could potentially be shortened). Tree fruits have the additional environmental benefit of carbon sequestration and storage and could be used in agroforestry systems.
- Increasing the use of climate-controlled greenhouses (or underground, thermally insulated, or vertical agriculture) could help to increase production of fruits and vegetables throughout the year within the region and reduce the requirement for plant protection products and water use in water scarce regions (e.g. in exporting countries such as Spain). The potential for this measure could be highest in countries where the proportion of renewable energy is the highest and could potentially increase over time if the proportion of renewables in the energy mix increases.
- Root vegetables (including potatoes) and onions generally have the lowest environmental impacts per unit of weight and can be most easily stored, with relatively small inputs and little waste. To reduce the requirement for potato blight treatment and related loss of crop, crop rotation and genetic diversity are known options.
- Increasing the proportion of fruits and vegetables (of both imports and domestic supply) grown using less environmentally damaging practices, such as organic methods, and intercropping, would help to reduce the use of plant protection products and synthetic fertiliser, and potentially provide biodiversity benefits by, for example, increasing landscape heterogeneity.
- One way to reduce the overall impact of fruit and vegetable production is to increase the proportion that reaches the market by reducing the stringency of shape/size/quality requirements in turn increasing the proportion of ‘ugly’ fruits and vegetables available for consumer purchase. The practice of removing products from the market that do not conform to pre-set standards is greater for fruits and vegetables compared to cereals and dried legumes – hence, there is most potential to utilise this measure as a way to reduce the environmental impacts of fruits and vegetables.
- Some environmental issues, such as soil health and biodiversity, are not captured by LCA. Hence, additional assessments would be required to avoid soil damage and biodiversity loss and ensure, for example, that the richest soils are utilised for the highest yields, using methods that protect soil and allow for sustained production over time.

## NNR Food Group 5: Pulses (legumes)

### Global context

Pulses are dried seeds from legume plants and include chickpeas, lentils, dried peas, and dried beans. Legumes include soya beans, fresh beans, and fresh peas (110). At the global level, 336 mt soya beans, 26 mt dry beans, 23 mt green beans, 14 mt chickpeas, 14 mt dry peas, 9 mt dry cow peas, 6 mt lentils, 5 mt dry broad beans, 4 mt dry pigeon peas, and 2 mt green broad and horse beans (83).

For most pulses and legumes, the majority (~80%) of environmental impacts occur during the production stage (Fig. 8). Transport generally accounts for a small proportion of impacts for all products. Two-thirds of global soybean production occur almost equally in the US and Brazil, followed by Argentina which produces 11% (111). Hence, imports of certain products, such as soya beans, to the European market, for example, will tend to involve long distance transport (but not necessarily air freight). Drying and preserving pulses and legumes extends their availability throughout the year and reduces waste. Dried products also have a smaller energy requirement in relation to transportation, compared to frozen or canned products (due to a reduced weight and no refrigeration) (75).

In general, pulses and legumes are among the foods with the lowest relative environmental impacts – particularly peas and beans (Table 13 – note that other pulses and peas are in dry weight).

**Table 13:** Average environmental impacts per kg of retail weight: Pulses (legumes) (global) (2)

	Land use (m^2^/kg)	Greenhouse gas emissions (kg CO_2_eq/kg)	Acidifying emissions (g SO_2_eq/kg)	Eutrophying emissions (g PO_4_^3-^eq/kg)	Freshwater (L/kg)
**Tofu (soybeans)**	3.5	3.2	6.7	6.2	149
**Other Pulses (dry)**	15.6	1.8	22.1	17.1	436
**Peas (dry)**	7.5	1.0	8.5	7.5	397

Growing practices greatly influence the environmental impacts of pulse and legume production, in terms of both scale and type. Grown as part of crop rotations with cereals for example can provide numerous benefits including increasing the yield of subsequent cereal crops grown in the same area (as they use the nitrogen supplied by the pulses and legumes) and less requirement for plant protection products (e.g. crop rotations reduce the risk of fungal disease such as pea root rot), as well as increasing landscape-scale heterogeneity and its associated biodiversity benefits. As legumes and pulses fix nitrogen in the soil, they do not require nitrogen fertilisers which is a major environmental benefit. There is less requirement for tilling of the soil for legume and pulse production (112).

Despite their nitrogen-fixing properties, there are production practices that use high amounts of nitrogen fertiliser to increase yields – for example, soya bean cultivation in the USA (113). Cultivating soya beans in monocultures requires the use of chemical plant protection products. In terms of pesticide residue, soybeans can have the same proportion of non-compliance with permissible limits as for fruit and vegetables (114).

Land use change associated with pulse and legume production can substantially add to their environmental impacts. For example, adding the CO_2_ emissions from deforestation and burning of crop residues more than doubled the impact of soybeans grown in Brazil to 1.6 kg CO_2_ equivalents per kg soya beans (115). However, even with such additions, the climate impacts alone are relatively much smaller than most animal products such as beef, pork, chicken, and cheese (see Table 18). Land use change, such as deforestation, and the installation of monocultures with fertiliser and pesticide application can adversely impact the landscape and surrounding biodiversity. For example, heavy expansion of soybean cultivation on the Brazilian Cerrado is endangering important biodiversity. Despite being considered the most species rich savannah in the world, the Cerrado is the least protected ecosystem in Brazil. This process of land clearing for soybean cultivation has also been introduced to the Amazon rainforest region (though 48% of soy production occurs in the Cerrado region and 13% from the Amazon) (111). One consequence of such large-scale soybean cultivation is the infrastructure created to enable harvests to be transported, which in turn opens up the region for further exploitation. Soybean cultivation in the Amazon region has largely resulted in the conversion of existing small-scale agriculture into large-scale farming with high use of machinery (and thus much less requirement for farmers), a dominance of monocultures, a lack of crop rotation, and ongoing soil erosion (75).

**Fig. 9:**
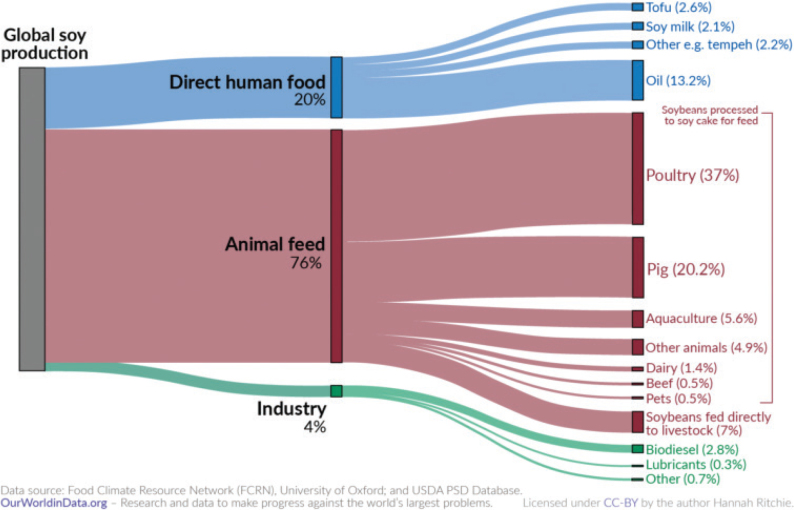
Allocation of global soy production to its end uses by weight (2017–2019) (111).

While soy production is a substantial driver of deforestation in the Brazilian Amazon, the expansion of pasture land for beef production is the leading driver. In addition, 95% of Brazilian soy is used for animal feed (111). Globally, the picture is similar – soy production is estimated to be the 3rd largest driver of deforestation (with cattle and palm oil being the 1st and 2nd largest drivers, respectively) (116). Only 7% of global soy production is used to produce tofu, soy milk, and tempeh directly for human consumption, and around 13% is used to produce oil for human consumption. Most soy (76%) is used to feed farmed animals – largely chickens and pigs (Fig. 9) (111). However, an analysis of soy embedded in food consumption across the EU found that farmed fish had the largest amounts per unit of product, followed by chicken meat (117). Globally, a small portion of soy production is certified as deforestation-free.

Around a quarter (23%) of pulses produced in 2019 were used for farmed animal feed (118). Reducing the consumption of animal products, particularly chicken and pork, is therefore the most effective way to reduce the environmental impacts of soy production, and also, but to a lesser extent and concern, pulse production. Replacing portions of beef consumption with pulses can also reduce environmental impacts considerably – for example, by reducing GHGs to make a substantial contribution to national climate goals, while also sparing large areas of agricultural land (119). Due to the inefficient conversion of plant nutrients to animal nutrients, the environmental impacts of humans consuming pulses directly are substantially lower (90).

### Nordic and Baltic context

**Fig. 10:**
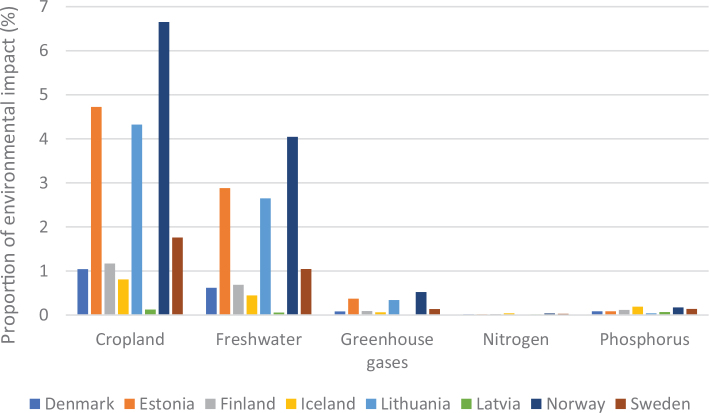
Impacts of pulse (legume) consumption across the Nordic and Baltic countries in relation to the food portion of global environmental limits (%). Source: *Global Nutrition Report 2021 (**17**).* The analysis utilises country-specific food consumption and environmental footprint data and relates them to the food portions of global environmental limits that is a global test to assess the impacts if everyone in the world consumed at the given rate. The methods and data are described in Box 1 and presented in Appendices 1–3.

Pulses (and legumes) account for low environmental impacts within the Nordic and Baltic diets. Due to the nitrogen fixing process during production, pulses account for little nitrogen use in relation to consumption across the Nordic region. Cropland and freshwater use tend to account for the majority of impacts, particularly for consumption in Norway (Fig. 10).

Pulses and legumes are an under-consumed food group across the region. The most promising process for increasing consumption is in conjunction with a reduction in meat consumption that is a replacement of portions of meat with legumes/pulses, which would maximise environmentally beneficial outcomes and potentially health benefits. Such a shift is considered a strong motivator for exploring options to increase local production. In addition, there are issues related to imports such as deforestation from soy. For example, soy imports to Denmark have been estimated to use a land area equivalent to the combined land area of Zealand and Lolland, and are considered to pose the greatest risk (in comparison to timber, pulp, and paper), as 65% of the land used is located in countries that are high or very high risk for deforestation and social challenges (120). In general, there is potential to increase pulse/legume production across the region. For some countries, current plans revolve around increasing pulse production as part of a crop rotation for the purpose of farmed animal feed (e.g. Norway). There are limits regarding the extent that pulses and legumes can be grown for direct human consumption in terms of variety, as lentils and chickpeas might be limited to certain areas and even the production of broad beans is limited in Finland. There are ongoing chickpea trials in Denmark, for example, with yields being weather dependent.^21^ There is also potential for learning from other regions with similarities in weather and climatic conditions that have recently started to produce chickpeas on a commercial scale [e.g. the UK (121)].

Some countries are more favourably positioned geographically for pulse production (e.g. greater potential in southern Sweden compared to Norway or Finland). Generally, peas and various types of beans grow well in the Nordic region (peas were historically grown on a larger scale for direct human consumption before shifting agricultural production to favour animal agriculture). Hence, there is a legacy of pulse and legume production in the region, which can be utilised to guide the expansion of current production. It is important to increase research in support of increasing pulse and legume production, including breeding and field trials (which might also involve increasing the grade of crops from animal feed to a grade high enough to enable direct human consumption) – and also providing appropriate training for practitioners to overcome a lack of experience in cultivating legumes, including the use of technologies to increase protein content and crop rotations techniques (75, 122–124). In Sweden, production is currently limited to one area and certain types of legumes/pulses (fava/broad beans and yellow peas), although it is possible to grow hardy varieties of soya beans in southern Sweden (75). However, changes in climatic conditions could also change the opportunities for pulse and legume production (e.g. in Sweden and southern Finland). The use of greenhouses and/or tunnels could increase the production of soy and broad beans. Therefore, the growth of pulses and legumes in the region could be considered dynamic rather than being limited to current opportunities. There is also an issue of scale. For example, in Sweden production tends to be niche and limited to local gardens, and hence would require scaling up (this is also an aspect where learning from other countries such as the UK could be particularly useful for informing how to scale from niche to commercial level). Due to the relatively small scale of production, pulses are generally lacking compared to other crops in terms of investments to increase yields, and research on pests and diseases.

An increasingly prominent area of research regarding pulse production and consumption across the region relates to their use in various meat-replacement products, or meat alternatives. For example, in Sweden the broad beans and yellow peas are currently used to produce tempeh. Increasing plant-protein production for such products is a growing field of emerging research in Finland. For example, in Finland, there are large research projects such as ScenoProt (125) and ‘Leg4Life’ (126) which aim to develop, with stakeholders, cultivation methods to increase production of legumes that flourish in Finnish conditions (pea, faba bean, lupins, and clovers) and processing methods to create legume products for animal feed and for direct human consumption. Such an initiative could help overcome a reliance on soya beans in processed products, which is apparent due to a lack of processing opportunities for domestically produced legumes (75). Research in this field has also increased in Denmark where old pea varieties grown for human consumption are being investigated in the project Peas & Love (covering pea yield stability, taste, and quality), and organic broad beans cultivation in the project ØkoFaba. In Norway, projects exploring the opportunities for growing legumes in a warming climate have yielded promising results (e.g. FoodProFuture and VOM).

For some countries (such as Iceland), a dependency on imports is likely to remain due to the limited potential for cultivating legumes and pulses, and for others (such as Norway) a reconfiguration of existing cropland, in addition to fixing the current yield gap (127), would likely be required due to a limited availability of cropland. For example, replacing a proportion of crops currently cultivated for animal feed with pulses or legumes for direct human consumption (128). If implemented as crop rotations with cereals, such a reconfiguration would help to tackle a number of fungal diseases that impact cereals. It is possible that some domestically grown pulses and legumes could at least partly replace imports; for example, brown beans are a good alternative to kidney beans imported from the US (75). In some regions and for some crops, there is also an element of cultural heritage related to legume production [e.g. brown beans on Öland, where farmers can claim environmental subsidies for conserving local varieties and farming methods (75)]. Legume production can have positive impacts on the landscape and biodiversity and contribute to a varied agricultural landscape (128). The specific opportunities to cultivate pulses and legumes within the region might be influenced to some extent by permissible plant protection products, as this is not the same in every country.

Currently, all countries produce pulses – with Latvia being the highest producer and Lithuania the lowest producer. Finland, Lithuania, and Norway depend to an extent on imports to meet supply (Table 14).

**Table 14:** Pulse^22^ production and supply across the Nordic and Baltic countries in 2019 (84)

Country	Production (1,000 tonnes)	Imports (1,000 tonnes)	Exports (1,000 tonnes)	Balance (1,000 tonnes)	Food supply (Kg/person/year)
**Denmark**	85	77	45	117	1
**Estonia**	111	4	67	48	5.7
**Finland**	64	28	0	92	2.6
**Iceland**	(no data)	0	(no data)		1.1
**Latvia**	102	59	132	29	0.4
**Lithuania**	306	19	270	55	2.6
**Norway**	30	584	2	612	12.6
**Sweden**	131	49	22	158	2.5

For comparison, pulse/legume consumption in line with a flexitarian diet that aligns with planetary boundaries (if implemented in combination with medium-ambition technological measures to reduce inputs during food production, and reductions in food loss and waste of 75%) recommends a minimum daily pulse/legume intake of 75 g, or 27.4 kg per year (as part of an average daily intake of 2,100 kcal) (14). Based on the annual supply data in Table 14 and consumption data in Table 2, all countries consume far below this recommended minimum intake (on a per person basis).

Peas are generally the most highly consumed across all countries, except for Iceland where beans are the most highly consumed (Table 15).

**Table 15:** Pulse (legume) food supply across the Nordic and Baltic countries in 2019 (kg/person/year) (84) by pulse type

	Beans	Peas	Pulses, other	Soybeans
**Denmark**	0.1	0.5	0.3	0.2
**Estonia**	0.05	5.5	0.01	0.2
**Finland**	0.03	2.3	0.08	0.2
**Iceland**	0.5	0.02	0.2	0.4
**Latvia**	-	-	0.2	0.2
**Lithuania**	0	1.6	0.9	0.1
**Norway**	0.1	7.4	4.8	0.3
**Sweden**	0.2	1.7	0.3	0.3

#### Considerations for reducing environmental impacts of legume and pulse consumption in Nordic and Baltic countries

From the overview of legume and pulse production, consumption, and trade provided in this section, we identify a number of opportunities to explore as potential ways to reduce environmental impacts of the legume and pulse food group:

- Pulses and legumes have among the lowest relative impacts, from an environmental sustainability perspective, and have much lower impacts across the board in comparison to meat, for example, whether the pulses/legumes are domestically produced or imported. Reducing the consumption of animal products and replacing portions of them with legumes and pulses is the major route to reducing overall environmental impacts while simultaneously increasing pulse/legume consumption. Reducing the absolute amount of beef, pork, and chicken production in particular could reduce the substantial environmental impacts (including deforestation) of soya bean production given that most (76%) soya beans are used for animal feed.
- Reducing imports of soya beans for farmed animal feed is considered in Section 8 on meats and meat products.
- Crop rotations with legumes/pulses and cereals would reduce the need for plant protection products such as fungicides, and nitrogen fertilisers. Increasing production of legumes/pulses for direct human consumption within the Nordic region would also help to diversify agricultural production and landscapes, and reduce reliance on imports.
- Reducing the overall land requirements of agriculture at the global level (by reducing livestock and feed crop production, and increasing legume/pulse consumption) could have additional major benefits for biodiversity and climate change targets if native ecosystems and vegetation cover were allowed to recolonise spared land. This shift would also allow space for more environmentally friendly farming methods that typically have lower yields compared to conventional production methods (e.g. organic) (3).
- Traceability within the supply chain of imported pulses/legumes is not always possible. Hence, the environmental impacts of imported products are not always known. A precautionary approach would be to adopt a default organic (or similar) procurement policy, and adopt approaches to enable and incentivise organic (or similar) methods with crop rotations for production within the Nordic region.
- Innovation in legume/pulse production, manufacture and processing, and consumer behaviour could facilitate an increased production and consumption of legumes/pulses for direct human consumption.

## NNR Food Group 7: Fish, fish products, and seafood

### Global context

Globally, around 200 million tonnes (mt) of fish and seafood are produced every year (176,592,630 tonnes in 2019 (84)]. In recent decades, growth in aquaculture has supplied most demand growth while growth from wild caught fish has been limited due to protective measures taken in light of the well-established scientific consensus on the state of the world’s wild fish stocks. Farmed fish and seafood now contribute 53% to total global production of fish and seafood and are expected to continuously increase due to limited growth potential in the capture sector (129). The majority (84%) of wild-caught fish are consumed directly by humans, and 16% are used to feed farmed animals (including 11% for farmed fish). China produces the largest amounts of fish and seafood (60 mt in 2017), followed by Indonesia, India, Vietnam, and the US (130).

Capture fisheries and aquaculture both have a range of environmental impacts, which vary depending on the habitat types, production method, and equipment type – ranging, for example, from intensive and higher-input shrimp and salmon farming to lower-input mussel farming in aquaculture; and from lower bycatch and lower fossil fuel pole and line fisheries, to benthic trawl fisheries with significant bycatch, damage to habitats and fossil fuel use. There are also dependencies between the two – for example, capture fisheries providing feed inputs to aquaculture; and various forms of pollution from aquaculture impacting capture fisheries (129). However, some impacts of capture fisheries are particularly difficult to measure – for example, in terms of catch, only the fish brought back to land (‘landings’) – are recorded in most databases, such as those of the UN fisheries. Discards are not reported, making it impossible (or very difficult and with uncertainties) to assess fish stocks (130). Despite producing only 1% (by weight) of food and feed for farmed animals globally, aquatic systems (wild and farmed) have been estimated to account for 10% of the footprint of all food produced in terms of GHG emissions, freshwater use, habitat disturbance, and nutrient pollution (1).

In terms of GHGs, the main impact from capture fisheries is fossil fuel use for fishing vessels [although emissions from shipping of exports can be higher, for example, seafood from Norway to Asia (131)], while the main GHG impact for aquaculture comes from feed production [although air freighted exports can have a higher impact, for example, farmed salmon from Norway to Asia (131)]. The impacts of climate change are expected to affect capture fisheries and aquaculture. For example, high-latitude regions will experience an average increase of 30 to 70% in terms of overall catch potential, while a decreased catch of up to 40% is likely to be experienced in tropical areas (129). Warming seas due to global temperature rise could reduce the nutritional content of fish – for example, plankton living in cold (−2°C) regions contain three times more unsaturated fatty acids than those in warm (29°C) waters (132). In terms of aquaculture, even small temperatures can impact productivity of many farmed species, in addition to creating new opportunities for diseases and parasites. Increased frequency and intensity of adverse weather could impact farm infrastructure, and land-based operations could experience freshwater shortages. Supply of crop-based feeds could also be threatened (129). Climate change could therefore negatively impact food webs and fisheries (132).

**Fig. 11:**
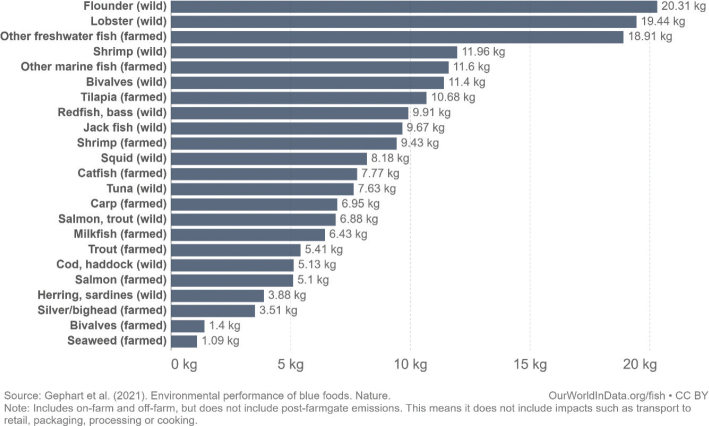
Greenhouse gas emissions per kilogram of edible fish and seafood.

Figure 11 provides a range of GHG footprints associated with fish and seafood production, based on data from 1,690 fish farms and 1,000 unique fishery records. The 23 represented species groups cover over 70% of global ‘blue food’ production (133). Generally, there is no clear size difference between GHG footprint in terms of capture vs farmed – both production types have a range of footprint sizes depending on species [although GHGs from some methods have been difficult to quantify, for example, bottom trawling, and require further investigation (134)]. Small pelagic fishes (herring, sardines) generate lower emissions than all fed aquaculture, and flatfish and crustaceans generate the highest amounts. When comparing the same species, farmed fish tend to have lower GHG footprints compared to wild caught (e.g. salmon and shrimp) – with the difference being most substantial for bivalves. However, there are examples where wild caught salmon have lower GHG footprints compared to farmed salmon. For example, a recent assessment of Norwegian salmon production found GHGs from wild caught to be up to 86% lower than farmed – which also had higher land use requirements and marine ecotoxic and eutrophying emissions (135). Seaweed has the smallest GHG emissions across the range of seafood (Fig. 11).

**Fig. 12:**
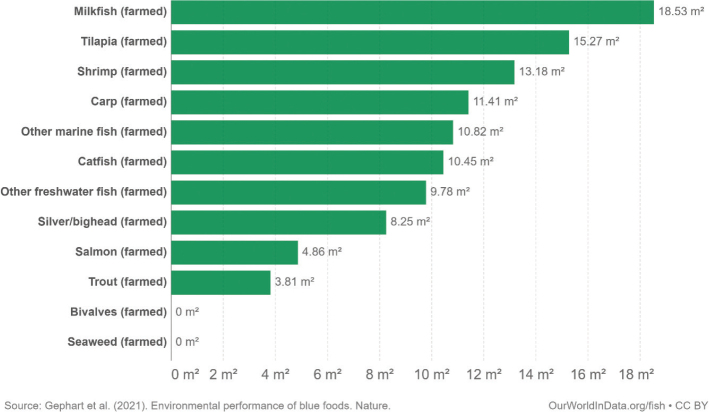
Land use per kilogram of edible fish and seafood (133).^23^

The land requirements for aquaculture result mostly from feed production, and less so from the conversion of terrestrial areas to fish farms, which account for 60% of global aquaculture production (10% of production is from coastal ponds and the remainder is from open-water cages and ropes, in seas and in lakes). Around 70% of farmed fish are given supplemental feed inputs derived from agriculture. Farming of unfed fish species like filter feeding molluscs accounts for around 24% of aquaculture production and put less pressure on land; however, this type of production is growing at a slower rate compared to fed species (129). Figure 12 shows a range of land use footprints from aquaculture, including land used for farms and feed production. The variation in land use between species can be substantial (e.g. milkfish vs trout). Bivalves and seaweed have the smallest land use requirements. One key consideration regarding land use is the opportunity cost that is other competing land uses, or uses that provide greater levels of social and environmental benefits in comparison. Growing crops for fish feed rather than crops for direct human consumption is a growing tension (129).

**Fig. 13:**
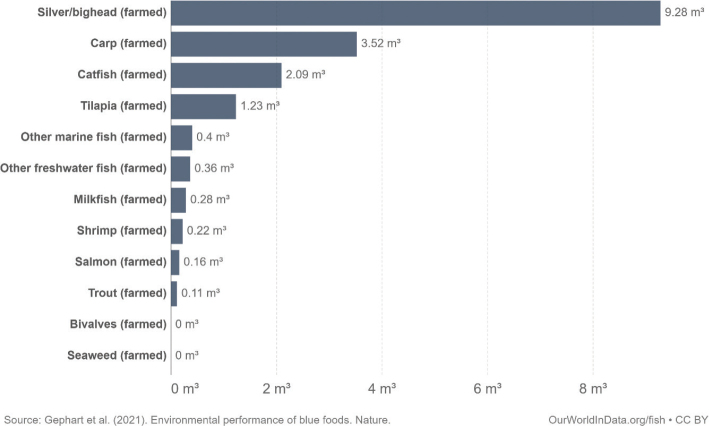
Freshwater use per kilogram of edible farmed fish and seafood (133).^24^

Freshwater use in capture fisheries relates almost entirely to post harvest activities. Direct freshwater use in aquaculture can be large for some pond-based systems but can vary significantly depending on system characteristics, location and targeted species. Some forms of aquaculture ‘recycle’ water by using it temporarily on the farm and releasing back in a more polluted form (e.g. added nutrients and chemicals), which might subsequently reduce its useability and/or effect nearby ecosystems. Water use for feed crops can also be significant (129). Figure 13 shows water use footprints for a range of farmed species. As for GHGs and land use, water usage can vary substantially between species, with trout being the fish with the smallest footprint. Bivalves and seaweed do not require freshwater for production, making them particularly favourable at least from a water scarcity perspective.

**Fig. 14:**
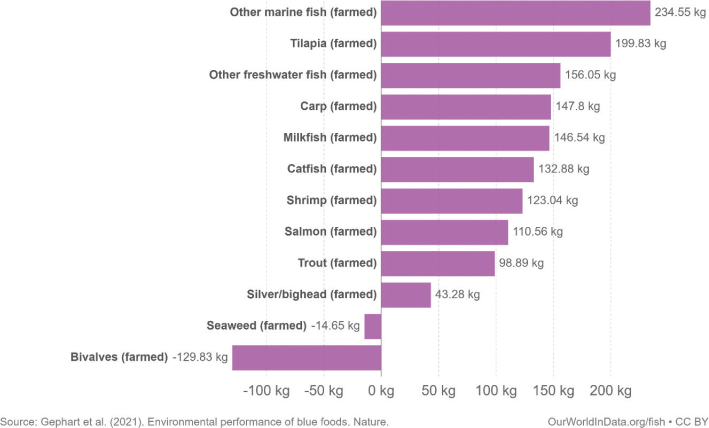
Nitrogen emissions per kilogram of edible farmed fish and seafood (133).^25^

**Fig. 15:**
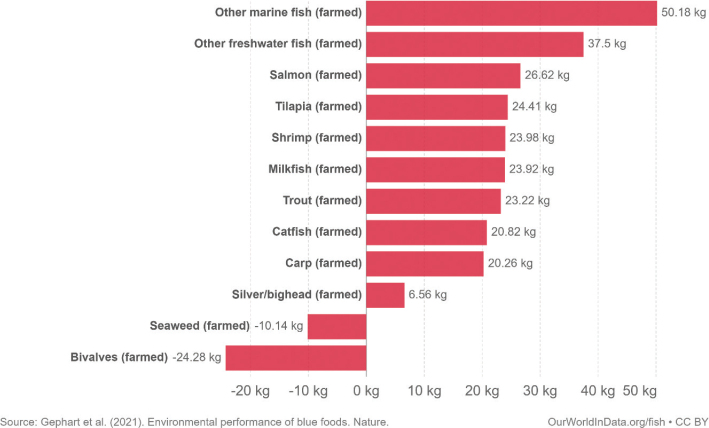
Phosphorous emissions per kilogram of edible farmed fish and seafood^26^

Nitrogen (N) and phosphorus (P) emissions from direct nutrient leakage from aquaculture farms, and also from the agricultural production of feed contribute to marine and freshwater eutrophication. Figures 14 and 15 show nitrogen and phosphorus footprints of a range of species produced from aquaculture. In fed systems, most N (>87%) and P (>94%) emissions occur on-farm. Silver/bighead fish have the lowest N and P footprints, while seaweeds and bivalves result in negative emissions due to being produced in extractive systems that remove more N and P than is emitted. Changing the feed type can change N and P impacts – for example, replacing feed for rainbow trout with Baltic herring could reduce the total eutrophication impact of rainbow trout. The impacts could be further reduced if the wild caught herring replaced the farmed trout (136).

Both capture fisheries and aquaculture have a range of adverse impacts on biodiversity. In relation to capture fisheries, over-harvesting, bycatch, and destruction of habitats through equipment are the major routes. Land and aquatic space conversion for farms, effluents (such as nitrogen and phosphorus emissions) to the local environment, and direct killing of wildlife (e.g. if they are a predator of fish) are the major routes for aquaculture. Genetic introgression from escaped species has also been reported (137). For some species (e.g. shrimp), aquaculture requires the capture of larvae from the wild, which also has bycatch impacts. Biodiversity impacts related to feed production (including habitat loss and degradation, use of plant protection chemicals, and nutrient pollution) also apply to fed aquaculture.

Currently, 92% of the total ocean is unprotected (i.e. is not governed by a global treaty), and 82% of national waters are also unprotected (130). Globally, 34% of fish stocks are overfished, 60% are maximally fished and 6% are underfished (according to the most recent estimate for 2017) (138). The Mediterranean and Black Sea have the highest proportion (63%) of stocks fished at unsustainable levels, followed by the Southeast Pacific (54%), and Southwest Atlantic (53%) (138). A number of species are estimated to be in a particularly dangerous state. For example, mackerels are estimated to be below optimal levels due to an increase in fishing intensity. Sharks are rapidly declining – and 37% of all sharks and rays are threatened with extinction. Southern Bluefin tuna need more time to recover to optimal levels. However, there are a number of issues that make such estimates of fish stocks unreliable. First, only the catch brought to land is counted in most databases (bycatch/discards is not counted), and data is much more sparse regarding the status of fish stocks across Asia, Latin America, and much of Africa, in comparison with Europe and North America. Assessing the status of fish stocks requires data on catch and how quickly fish populations recover. While there are measures in place to overcome some of the data issues (including an assessment every decade by the UN Fisheries Division), such data gaps present a major hinderance to asserting what is sustainable or not (130).

It has been estimated that most discards (93%) result from large, industrial scale fisheries with the largest discards associated with bottom trawling – on average, 21% of catch from bottom trawling is discarded, increasing to over 50% for shrimp trawling. In terms of global fish discards, bottom trawling is estimated to account for around 50% of the total fish discards from all methods (130). In addition to high discard rates, trawling physically damages ecosystems. The extent of the damage largely depends on the depth of the trawl into the sediment (although sediment type and lifeforms in the area are also important). On average, 6% (from Otter trawl) to 41% (from hydraulic dredging) of faunal biomass per pass are removed, with recovery of the damage taking 1.9–6.4 years post-trawling, depending on fisheries and environmental context (139). Trawling, for example, in the Barents Sea affects the biomass of all species but especially those easily caught by a trawl (140). Around a quarter of global annual fish catch is from bottom trawling, and it is the dominant method used in China and India. Purse seine (vertical nets) accounts for 20% of global annual catch and is also more commonly used in industrial fishing practices as, also for trawling, these methods tend to result in a higher yield. Purse seine is, in some cases, also associated with high levels of bycatch. The lowest levels of bycatch are associated with pole-and-line, and longline methods which are more commonly used in lower income countries for subsistence or small-scale capture (130).

There is growing evidence that seabed habitats throughout the world’s oceans are being impacted by physical destruction or selective removal of habitat-forming species. Removal of the latter, or drastic lowering of the population, can impact entire food webs and ecosystem functioning (particularly if apex predators are removed/reduced, and predator-prey interactions are impacted) (129). One consequence is a reduction in vegetation where predator populations are too low and allow herbivorous species to flourish – in turn reducing the carbon storage capacity of marine environments (141). In terms of habitat disturbance, demersal fish (also known as groundfish and include cod and haddock), are estimated to have by far the largest impact from capture fisheries, and shrimp have the largest impact from aquaculture. However, disturbance from capture fisheries tends to be much greater in comparison to that from aquaculture even when accounting for disturbance from feed production (1).

Aquaculture can also impact entire ecosystems, for example, freshwater and brackish-water pond farming has driven large-scale local and regional landscape transformations including large areas (~60,000 km^2^) of ecologically valuable coastal agricultural land and wetland habitats (mainly along South China, India, Vietnam, Indonesia, and Bangladesh), that have been fragmented through land reclamation and conversion. Mass escapes from fish farms resulting in cross breeding and behaviour change in wild salmon is an ongoing issue, as is pathogen spread to wild species, in addition to nutrient waste polluting local environments (129).

Ocean acidification (OA) resulting from increasing atmospheric concentrations of CO_2_ has been identified as a substantial threat to marine ecosystems, and both capture fisheries and aquaculture – and wider food systems – contribute through fossil fuel use (including direct and indirect use). Capture fisheries are expected to be impacted by OA, as the distribution and catchability of over a hundred fish species could decline by up to 30% (129). Warm water coral reefs and calciferous marine organisms are among the marine biodiversity impacted by OA.

The fishing industry contributes substantially to ocean plastic pollution – for example, over 46% of the mass comprising the great pacific garbage patch is fishing nets (142). Such waste from the fishing industry impacts biodiversity in a number of ways, including ingestion of plastic and entanglement in discarded nets and equipment.

As with other forms of animal farming, animal welfare and antibiotic use are important issues to consider. Antimicrobials are commonly used in aquaculture, contributing to Anti Microbial Resistance (AMR). While these topics are outside of the scope of the current paper, they are important issues to consider in any assessments of sustainable food systems and diets.

#### Nordic and Baltic context

**Fig. 16:**
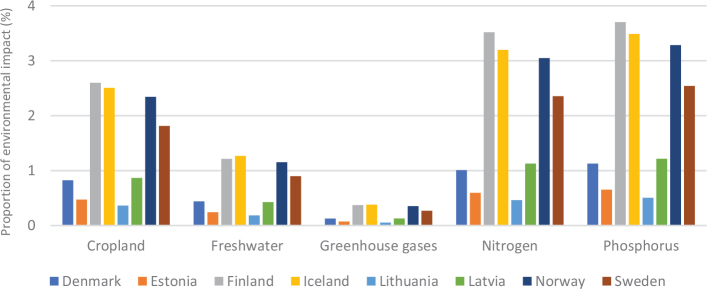
Impacts of fish consumption across the Nordic and Baltic countries in relation to the food portion of global environmental limits (%). *Source: Global Nutrition Report 2021 (**17**).* The analysis utilises country-specific food consumption and environmental footprint data and relates them to the food portions of global environmental limits that is a global test to assess the impacts if everyone in the world consumed at the given rate. The methods and data are described in Box 1 and presented in Appendices 1–3.

Figure 16 shows the variation in environmental impacts of fish consumption across the Nordic and Baltic countries and how they relate to global limits for food consumption. Nitrogen, phosphorus, and cropland tend to account for the majority of impacts.

A comprehensive assessment of environmental impacts of fish capture is difficult – particularly in relation to the biodiversity impacts. The lack of data availability regarding fish stocks makes an assessment of the environmental sustainability of capture rates impossible (due to the lack of frequent and comprehensive data collection on discards). Similarly, assessing the biodiversity impacts is impossible without reliable information on bycatch, or data on physical damage to ecosystems via trawling methods. The environmental impacts of aquaculture are generally less challenging to assess, with the exception of biodiversity. A lack of data availability needed to assess environmental sustainability in a comprehensive way, combined with an expected increase in global fish production (mostly via aquaculture) and the impacts of environmental shocks and climate change make a precautionary approach to recommendations for fish consumption important that is more information is required before any targeted increase in fish consumption in the Nordic and Baltic countries could be justified from an environmental sustainability perspective, and also from a food security/risk perspective. This might include identifying under-utilised freshwater fish stocks, shifting supply from animal feed to human consumption, and assessing the balance of impacts in different environments including the potential to reduce eutrophication in fresh water bodies. Other potential options to explore (particularly to reduce pressure on capture fisheries) are mussels, algae, and seaweed. For example, mussel farming already takes place along the west coast of Sweden and could provide an important case study in terms of environmental impacts and scalability.

Increasing aquaculture production could be an issue in terms of feed production from agriculture and the associated impacts (e.g. see chapter 5 for more information on the environmental issues related to soya production). An increased demand for feed necessarily raises the issue of feed vs food in relation to food security, and competition with other land uses such as biofuel production and carbon capture (this issue applies to all animal farming to varying extents, see for example chapter 8 for more information). Due to their inefficient conversion of shorter-chain fatty acid, α-linolenic acid (ALA; 18:3n-3), into eicosapentaenoic acids (EPA) and docosahexaenoic acids (DHA), salmon along with other cold water marine species of fish must obtain n-3 LC-PUFA through their diet (fish oil and fish meal in aquaculture). However, changes to feed composition (including an increased use of oil seeds), in response to rising demand for fish oil and fish meal, have resulted in a reduced nutritional benefit to humans consuming the farmed fish (143). Hence, there is scope to explore direct sources of n-3 fatty acids for human consumption as an alternative to consuming fish from aquaculture, such as microalgae already in use as a n-3 fatty acid supplement.

Increasing production from capture fisheries is limited in terms of location across the Nordic and Baltic region due to high levels of pollution in the Baltic Sea and also lakes in Sweden, for example, which could make it problematic to recommend local production. Hence, for some countries such as Sweden, increasing fish production would likely be from aquaculture. Otherwise, an increase in consumption might be met through imports from Norway, Denmark, or Iceland, for example, which are all large producers and exporters (Table 16), or through increasing production in lakes and capturing wild freshwater fish throughout Finland. In these scenarios, it would still be necessary to reduce the overall environmental burden of fishing and fish consumption to prevent problem-shifting, for example, reducing one environmental issue in one location while increasing them in another location, and also to contribute to a range of environmental targets. One aspect to consider in relation to the environmental impacts of importing (and exporting) fish is the use of air cargo. A full assessment of the environmental impacts of capture fisheries (including production methods) and aquaculture (including nutrient emissions and the feasibility of implementing technologies to reduce them, and antibiotic use) across the region would be required in order to identify optimal pathways (20).

**Table 16:** Fish (seafood) production and supply across the Nordic and Baltic countries in 2019 (84).^27^

Country	Production (1,000 tonnes)	Imports (1,000 tonnes)	Exports (1,000 tonnes)	Balance (1,000 tonnes)	Food supply (Kg/person/year)
**Denmark**	942	1,214	1,955	201	27
**Estonia**	84	50	112	22	15
**Finland**	205	134	73	266	34
**Iceland**	1,184	65	1,146	103	91
**Latvia**	119	88	134	73	25
**Lithuania**	94	196	197	93	33
**Norway**	3,677	1,222	3,105	1,794	51
**Sweden**	247	1,075	962	360	32

Norway is the largest producer, importer, and exporter across the Nordic and Baltic countries (Table 16), and the world’s second largest seafood exporter (144). In Norway, a discard ban, which comprises of several measures, has possibly significantly decreased discards (145), although monitoring is challenging (146). Iceland and the EU also have discard bans (the ban in the EU is less restrictive compared to the ban in Norway). The discard ban in the EU was launched in 2017, and its impacts have not yet been estimated (146). Fisheries management is international in scope, meaning that making changes to fisheries in Nordic/Baltic seas (e.g. to harvest rate, selection pattern, discard bans) needs to be decided with other countries with which the stocks are jointly managed through international agreements (e.g. the EU, UK, and Russia). For example, about 90% of Norwegian fisheries, both in value and volume, are taken from stocks it shares with other countries (147). However, given that Norway is the tenth largest producer (84) and second largest exporter of fish and seafood globally, it could have more potential to influence such decision making compared to the other Nordic and Baltic countries.

Iceland currently has the highest levels of fish and seafood supply across the region – almost double the amount consumed in Norway, which has the second highest supply levels (per person). Estonia has the lowest supply levels, around 6 times lower than Iceland (Table 16). For comparison, fish and seafood consumption in line with a flexitarian diet that aligns with planetary boundaries (if implemented in combination with medium-ambition technological measures to reduce inputs during food production, and reductions in food loss and waste of 75%), recommends a minimum daily fish and seafood intake of 28 g, or 10.2 kg per year (as part of an average daily intake of 2,100 kcal) (14). Based on the annual supply data in Table 16, all countries exceed this amount (on a per person basis) 1.5–9 fold. In contrast, using fish consumption data from Table 2 indicates that consumption in Denmark, Iceland, Sweden, and Norway exceeds the minimum amount consistent with the flexitarian diet – all other countries consume close to or slightly below this amount.

One assessment of fish production suggested at the global level a maximum amount of 3.5 kg per person per year would accord with a sustainable load on fish stocks (maximum sustainable yield of ~66 mt from capture fisheries in 2030) (148). Assuming around 50% (in line with the global production split between capture and aquaculture) of consumption within the Nordic and Baltic countries is sourced from capture fisheries, an annual consumption of 3.5 kg is around half of the amount consumed in the lowest consuming nation (Estonia) and 13 times lower than the highest consuming nation (Iceland) within the region. However, this estimated sustainable consumption could be questioned because of large uncertainties due to lack of available data, as described above – a comprehensive assessment of sustainable fish and seafood yields in the Nordic and Baltic countries is needed (20).

**Fig. 17:**
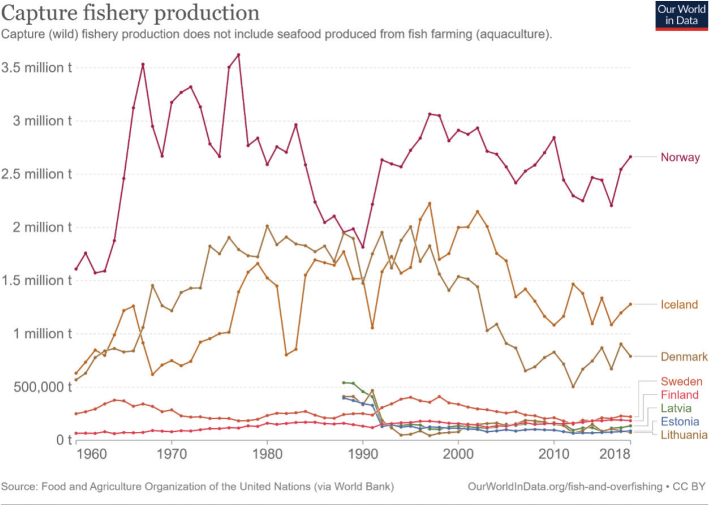
Capture (wild) fishery production (tonnes) in the Nordic and Baltic countries: 1960–2018.

For all countries, capture fisheries represent by far the largest proportion of fish production – the biggest contribution from aquaculture is in Norway, where it accounts for around a third of total fish production (Figs. 17 and 19). Production levels from capture fisheries have generally declined in Norway, Iceland, Denmark, and Sweden since 1997 (Fig. 17).

**Fig. 18:**
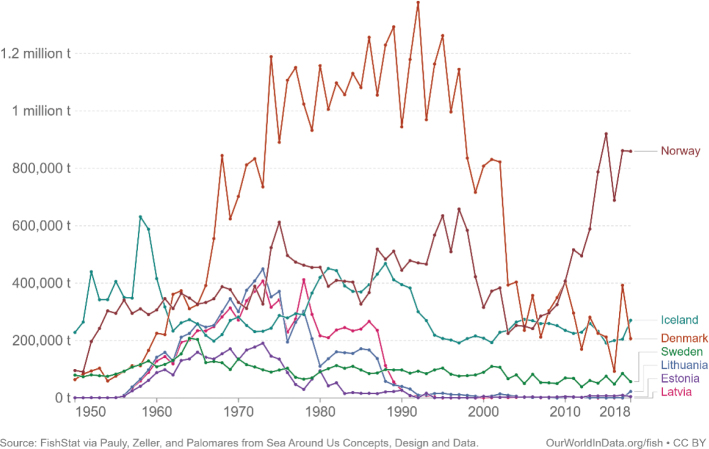
Wild fish catch (tonnes) from bottom trawling in the Nordic and Baltic countries: 1950–2018

Capture from bottom trawling has also generally declined from 1997 levels, except for Norway and Iceland, where bottom trawling has increased (Fig. 18). Bottom trawling is used for around a third of fish capture in Norway and around 21% of fish capture in Iceland.

**Fig. 19:**
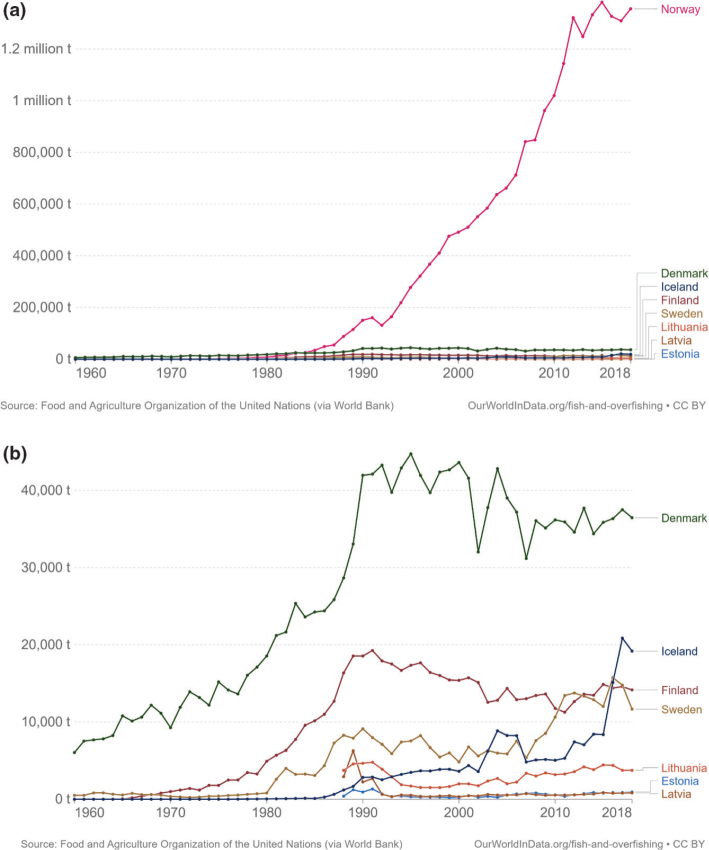
(A) Aquaculture production (tonnes) in the Nordic and Baltic countries: 1960–2018; (B) Aquaculture production (tonnes) in the Nordic and Baltic countries: 1960–2018 (excluding Norway to enhance visibility of production amounts from the remaining countries).

Norway has by far the largest production from aquaculture, which increased sharply from 1985 to a current production of around 1.3 mt per year (Fig. 19A). Across all countries in the region, the main production is fed finfish, dominated by salmonoids (salmon, trout, and char). The variation in environmental conditions between the countries makes some of them more favourably positioned for aquaculture and impacts sustainability considerations. For example, Iceland, Norway, and Denmark benefit from access to long coastlines with strong currents, whereas Denmark also has coastline towards the Baltic Sea, as do Finland and Sweden, which are highly polluted, populated, and has low oxygen levels, limiting the possibilities for large scale open-water aquaculture (149, 150).

The environmental impacts of most concern in relation to aquaculture production in Norway and Iceland relate to sea lice, escapes and disease and gene transfer to wild fish stocks, organic wastes, and land-use change concerns related to soy production (for feed). Nitrogen and phosphorus emissions, and organic wastes are the main concerns in Denmark, Sweden, and Finland. Within the region, the high reliance on inputs from agriculture (of which the high-grade ingredients could be used directly for human consumption) is common concern. In all settings, antibiotic treatments are of concern (150).

Nordic aquaculture policies currently focus on growth, both in terms of production volumes and economic gains (through high-value, fed-fish including salmon or trout), with strategies to intensify production through technology rather than shift to less environmentally burdensome species and production levels, and to replace soy with alternative proteins as feed. Hence, the priority appears to be an increased production with a reduced environmental impact per unit of production that is reducing relative rather than absolute impacts (150).

Combining supply from all production methods, pelagic fish dominate consumption in Denmark, Iceland, and Lithuania. In Estonia and Norway, demersal fish dominate; in Finland freshwater fish dominate; in Sweden the consumption of demersal and freshwater fish is equal, and higher than pelagic; and in Latvia ‘other marine’ fish dominate (Table 17). Iceland has the highest consumption of crustaceans, followed by Norway. This group includes lobsters and shrimps, which tend to have among the highest environmental footprints (especially from capture fisheries) (Figs. 12–14). Demersal fish tend to be mostly carnivorous and hence have different feed requirements and impacts within an ecosystem compared to herbivorous and planktivorous fish. Bluefin tuna (pelagic fish) are apex predators, which have a particularly important role in maintaining ecosystem functioning. Mackerels are also pelagic fish and are estimated to be below optimal levels due to an increase in fishing intensity. The sum of the unilateral quotas for mackerel and the resulting catches in the Northeast Atlantic and adjacent waters have exceeded the scientific advice by 41% on average since 2010 (151). However, it is generally unknown how much mackerel is consumed and where it is sourced from, in relation to consumption in the Nordic and Baltic countries. There is also lack of consensus on how the quota should be shared between the countries fishing this mackerel. Molluscs (a bivalve) tend to have among the lowest environmental impacts across the range of metrics (GHGs, land, water, nitrogen, phosphorus, and biodiversity) yet are consumed in lower quantities in comparison to other types of fish and seafood (except for cephalopods and other marine fish, which are consumed in lower amounts in most countries) (Table 17). Consumption data is not available for seaweed, which is the lowest impact seafood.

**Table 17:** Fish (seafood)^28^ supply across the Nordic and Baltic countries in 2019 (kg/person/year) (84) by fish type

	Pelagic	Demersal	Freshwater	Crustaceans	Molluscs	Cephalopods	Marine fish, other
**Denmark**	10	6	2	7	1.4	0.1	0.0
**Estonia**	2	7	1	3	0.6	0.2	0.0
**Finland**	10	3	18	2	0.2	0.1	1.2
**Iceland**	44	21	10	16	0.2	0.0	0.0
**Latvia**	8	4	2	1	0.5	0.2	10.0
**Lithuania**	28	1	2	2	0.4	0.3	0.0
**Norway**	6	23	11	10	0.8	0.1	0.1
**Sweden**	5	9	9	7	0.9	0.1	0.7

**Table 18:** Average environmental impacts per kg of retail weight: Meat and meat products (global) (2)

	Land use (m^2^/kg)	Greenhouse gas emissions (kg CO_2_eq/kg)	Acidifying emissions (g SO_2_eq/kg)	Eutrophying emissions (g PO_4_^3-^eq/kg)	Freshwater (L/kg)
**Bovine meat (beef herd)**	326.2	99.5	318.8	301.4	1,451
**Bovine meat (dairy herd)**	43.2	33.3	343.6	365.3	2,714
**Lamb & mutton**	369.8	39.7	139.0	97.1	1,803
**Pig meat**	17.4	12.3	142.7	76.4	1,796
**Poultry meat**	12.2	9.9	102.4	48.7	660

#### Considerations for reducing environmental impacts of fish and seafood consumption in Nordic and Baltic countries

Reducing fish consumption among high consumers in higher income countries with a good food supply will likely be important for reducing the overall environmental burden of the food system. In addition to the current issues regarding fish and seafood production, aquaculture is one of the fastest growing forms of food production globally and will continue to increase pressure on environmental resources and contribute to environmental problems. Challenges remain even for the most technologically advanced aquaculture systems. There is no single measure or innovation that will resolve all environmental challenges related to fish and seafood production (both capture and aquaculture); instead, a range of measures are required (129). We offer a range of considerations:

Major:

- Due to the potentially large-scale impacts on ecosystems and the largely unknown nature of fish stocks globally, a precautionary approach to this food group should be taken.
- A comprehensive assessment is needed to identify sustainable yields from marine and lake ecosystems within the Nordic and Baltic countries (20), including capture fisheries and aquaculture. It could also identify whether there are any sources of fish that on balance have positive environmental impacts. This should factor in potential impacts of climate change, environmental shocks, antibiotic use, animal welfare, waste, disease transfer and ecosystem-level impacts on biodiversity in addition to potential benefits such as removing nutrients from polluted waters. Due to changes in environmental conditions, regular monitoring, modelling, and recalculations will likely be needed.
- Underpinned by a precautionary approach, the assessment should also carefully consider if there is a need to increase consumption of fish and seafood, whether the environmental impacts of increasing consumption are adequately known, and the feasibility of shifting as much continued production and consumption as possible to the lowest impact foods [e.g. edible seaweeds which are particularly underrepresented in the literature (133)].
- The environmental impacts from all types of production should be minimised (this mostly applies to animal sourced capture and aquaculture). One important aspect for aquaculture is improving feed conversion ratios across all fed groups, which could reduce land and water use by up to 50%. Optimising capture equipment could reduce GHG emissions by more than 50% for some groups (133). However, it should be noted that minimising environmental impacts does not necessarily equate with environmental sustainability – an assessment will still be required to ensure production levels are consistent with environmental goals. In addition, impacts will increase if relative impacts are reduced but absolute impacts increase (due to an increase in production).
- Feed to food tensions including food security and efficiency aspects should also be considered, including the potential to reallocate resources used to produce feed to human edible foods.

Minor:

- Environmental impacts that are not included in the data presented in this chapter, for example, the energy used for drying seaweed and CO_2_ emissions during shell formation of bivalves, and energy used in the transportation of live bivalves, should be factored in (133).
- For any production of animals from aquaculture, the focus should be on bivalves (considering the previous point), which are a non-fed species with the lowest environmental impacts across a range of metrics. Currently, 30% of aquaculture production is ‘non-fed’ species (mostly carps and bivalves) (133).
- Environmental impacts of increasing imports and exports (such as those from air cargo) should also be factored into assessments.

## NNR Food Group 8: Meat and meat products

### Global context

At the global level, chicken meat is produced in the largest quantity (120 mt), followed by pig meat (110 mt), cattle meat (68 mt), sheep meat (10 mt), and goat meat (6 mt) – which together comprise the top 5 in terms of global meat production quantity in 2020 (84).

**Fig. 20:**
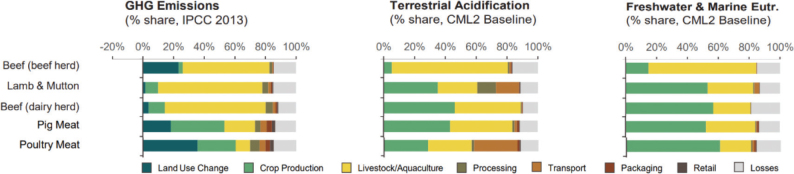
Proportion of environmental impacts from different life cycle stages: Meat and meat products (global) (2).

The environmental impacts of meat can vary substantially depending on the meat type and production method (Fig. 20 and Table 18). Across the entire product life cycle, most of the impacts from meat production occur during production (~80%), with a relatively small proportion resulting from processing, packaging, and transport. For some meat types and impacts, crop production (for animal feed) accounts for most of the burden, for example, eutrophication for beef meat production from dairy herds, pig meat, and poultry meat. Land use change accounts for the largest proportion of GHGs from poultry meat (Fig. 20). For the meats from ruminant animals (beef, lamb, and mutton), the largest proportion of GHGs arise directly from the animals in the form of CH_4_ from enteric fermentation during digestion (152). For beef meat, the majority of all impacts are directly associated with animal farming, for example, eutrophication and acidification from animal waste (Fig. 20).

Life cycle assessments of meat products have revealed a general trend in relation to impacts. Compared to meat grown using extensive (or organic) techniques, meat grown using intensive (or conventional) production methods tends to have lower impacts per kilogram across their life cycle in terms of land use, eutrophication, and acidification – but similar impacts in terms of GHGs and higher energy use (61). However, extensive methods might have lower impacts in terms of biodiversity loss and soil degradation (if production amounts were equal) (66). Extensive ruminant systems generally use a higher share of grassland and less arable land, utilise more permanent grasslands, and have higher organic matter applications to soil than intensive systems – all of which can increase soil carbon stocks in extensive production compared to intensive production (depending on current soil carbon content). However, soil carbon stocks are generally not included in such assessments due to a lack of data and methodological limitations (153, 154). The differences between the environmental impacts of intensive and extensive (and broadly conventional vs organic) production occur mostly due to a higher allocation of land (or area) and a longer lifetime of the animal under extensive methods due to a reduced productivity from a lower macronutrient density and digestibility of feed (61). Regardless of subsequent production methods, if initial land clearance for production displaces a native or pristine ecosystem such as primary forest, the impacts of such land use change could be substantial at least in terms of GHGs (43).

Depending on where and how feed crops are grown, they might also incur environmental impacts related to land use change, such as deforestation, including GHG emissions and biodiversity loss. Similarly, feed crop production might displace native carbon sinks such as peatland, where they are drained and converted to cropland. Relevant issues related to crop production (in this case, for feed production) are covered in chapters 3 and 5. Animals raised on pasture also have feed requirements, such as over winter where grass production may be less viable, and finishing feed to increase animal weight before slaughter. Pasture lands also have input requirements; for example, permanent pasture in the UK is the largest nitrogen user across all agricultural production (155).

Another important consideration is the localised, or site-specific impact. For example, manure management and/or use of fertilizsers and pesticides could have different impacts on the local environment depending on site-specific conditions, including the scale of operations, and due to nitrogen retention as a result of soils and drainage systems (156–158). Stocking density of farmed animals also plays an important role in determining the level and extent of environmental impacts (156). The severity of impacts could also vary depending on the types of inputs – for example, pesticides relatively high in toxic substances could have a more detrimental environmental impact compared to less toxic substances – however, this is generally not reflected by the most widely used environmental assessments (156) and therefore needs to be addressed in more detail in future studies.

While agricultural land used for grass production (including silage) can result in lower CO_2_ emissions from degradation of soil carbon stock per ha in comparison to agricultural land used for monocultures of annual crops (159), all land used for meat production, whether it is feed cropland (including silage) or pasture, and whether it is cultivated under organic or non-organic methods, has an associated ‘carbon opportunity cost’ related to the native vegetation cover that would occur if meat production ceased. Globally, this is mostly related to permanent pastureland that has replaced forest (43).

The production of meat, dairy, and eggs is the biggest single use of land globally – occupying 38% of all habitable land and 78% of agricultural land (2). The production of feed crops uses 43% of global cropland (2). Farming animals is a major driver of biodiversity loss – being the biggest source of nitrogen pollution (leading to oxygen depleted ‘dead zones’ in rivers and oceans through the process of eutrophication), ammonia deposition in landscapes, and land use and land use change, including deforestation (3, 160). For example, cattle farming is the single largest direct cause of deforestation, and soy production is the third largest (76% of soy production is used for animal feed, mostly to feed pigs and chickens) (111, 161). Biodiversity loss also occurs through the killing of animals that are considered detrimental to livestock farming, for example, through predation or spread of disease (162). Within key biodiversity areas, cattle farming has been identified as the largest single cause of biodiversity loss (accounting for 31%), with animal farming in total causing more than half of the loss of plants and vertebrates. From the perspective of land use, lightly grazed pasture was associated with half of all biodiversity loss – concentrated in middle- and low-income regions with rich biodiversity (163). Land classified as permanent pasture occupies 24% of global land area, and temporary pastures occupy 1% (27). The largest meta-analysis conducted on the biodiversity impacts of grazing farmed animals shows that only detritivores (e.g. dung beetles) benefit – all other species of animals and plants analysed decline in abundance and diversity in areas of livestock grazing (164).

#### Beef production

The impact of beef varies mostly depending on whether the meat is sourced from animals grown specifically for meat, or whether the meat is sourced from animals grown for both meat and dairy. For example, in terms of GHG emissions, according to a global average using retail weights, beef from a beef herd has around three times the impact of beef from a dairy herd [99.5 kg CO_2_e/kg compared to 33.3 kg CO_2_e/kg (2)]. The difference is 7-fold greater in relation to land use, but is lower in relation to acidifying emissions (0.9), eutrophying emissions (0.8) and freshwater use (0.5) (2) (Table 18).

Production methods also cause variation in environmental sustainability outcomes. Beef produced from grain-fed cattle uses less land and causes fewer GHG emissions and less eutrophication per unit of production compared to beef from grass-fed cattle, but has higher energy requirements. Cattle raised in both systems have feed, land, water, and energy requirements and produce manure – the environmental impacts of which can also vary substantially depending on management techniques (1, 2, 61, 156).

When considering such trade-offs at the aggregate or system level, demand for beef is an important consideration. If demand for beef declines substantially from current levels, there is more opportunity to focus production on extensive organic methods as the overall system-level land requirement for beef would decline. Adopting extensive organic beef production globally, and even nationally in some cases, would only be possible in combination with a substantial reduction in beef consumption from current levels. For example, a recent analysis of the US found that limiting production to exclusively pasture-fed beef would require reducing beef production to 27% of current levels – or a more than 3-fold increase in pasture area to continue current levels (165). Adoption of integrated mixed farming systems (e.g. extensive livestock coupled with diverse rotations) could be beneficial from a broad sustainability perspective, if demand for ruminant meat declined sufficiently to reduce incentives to expand agricultural land use (66). The main trade off (or opportunity cost) in this situation is the large area of land use that has typically displaced a more carbon- and biodiversity-rich habitat such as forest or grassland. If land-based climate change mitigation and biodiversity needs are considered to be more beneficial than livestock production, the land used for extensively raised organic cattle could instead be restored to native vegetation cover to maximise its carbon sink potential (such as reforestation of pastureland) and in turn provide opportunities for biodiversity that would occur in such native habitats.

Beef production has a substantial climate impact – accounting for 6% of total global anthropogenic GHGs (92). Farmed ruminant animals contribute the largest source of anthropogenic methane (CH_4_), accounting for around 30% of global emissions (166). While the environmental impacts of beef production could be to some extent reduced through various technological approaches (see section ‘general points across the food groups’), and through utilising waste streams from other systems for some portions of feed, these improvements alone are not enough. To align with planetary boundaries, reducing overall demand for beef at the global system-level remains essential even when optimistic improvements in production methods (to reduce environmental impacts per unit of production) are considered (6, 14, 42).

#### Pork production

Several of the issues and trade-offs related to beef production also apply to pig production in terms of large land use, biodiversity impacts, GHGs, feed requirements, and manure waste, albeit in different relative proportions and scales. Pig meat tends to have higher environmental impacts across a range of metrics in comparison to chicken meat, but much lower impacts in comparison to beef meat (including dairy herd) and sheep meat (Table 18). The largest proportions of overall environmental impacts from pig meat production tend to be a result of feed production and manure management (Fig. 20) (2, 167, 168). In terms of GHGs, feed has impacts via land use change (e.g. deforestation to create cropland for soybeans), and also directly in the form of nitrous oxide emissions from nitrogen fertiliser use and manure, and carbon dioxide from fuel used in field machinery and CH_4_ emissions from rice production. Hence, the total impacts of feed depend on whether they are driving land use change and the extent to which they generate impacts during production (152). Relevant issues related to crop production (in this case, for feed production) are covered in more detail in chapters 3 and 5.

#### Chicken meat production

Many of the same issues apply to the production of chicken, also a monogastric animal, as for pig meat production. Several of the issues and trade-offs related to land use, biodiversity, GHGs, feed requirements, water use, and manure waste are the same albeit in different relative amounts when assessed on a per weight unit basis, for example. As for pigs, the largest proportions of environmental impacts from chicken meat production tend to be a result of feed production and manure management (Fig. 20) (2). Again, the total impacts of feed largely depend on whether they are driving land use change and the extent to which they generate impacts during production (such as eutrophication of waterways) – see chapters 3 and 5 for more details on the impacts of crop production.

Chicken meat production has risen by 10% globally in the past 5 years alone (and is the fastest growing sector in terms of livestock production) (84). Another important consideration is the prospect of shifting from other types of meat with higher environmental impacts, to chicken which, in comparison to the top 5 most highly produced meats, tends to have the lowest environmental impacts across a range of metrics on a per kg basis (Table 18). However, to be able to assess the impact of shifting from other meat types to chicken, the full range of environmental impacts at the absolute level, and their relation to planetary boundaries, and other environmental risks (e.g. from increased use of antibiotics, spread of zoonotic disease, increased number of farmed animals) must be considered. The prospect of increasing deforestation for feed production is also relevant – for example, around 28% of soy produced globally is used to feed farmed chickens (Fig. 9).

#### Nordic and Baltic context

**Fig. 21:**
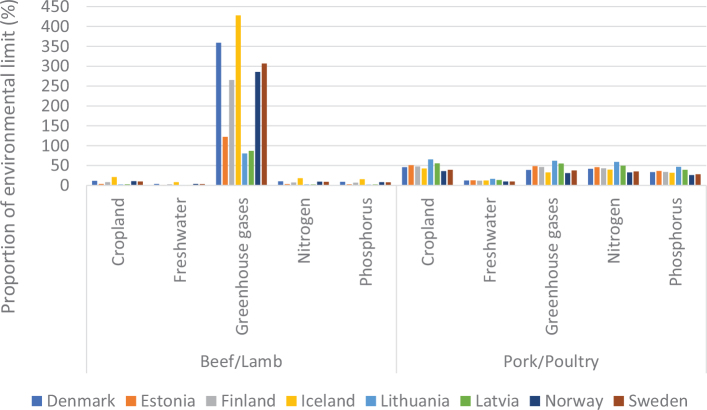
Impacts of meat consumption across the Nordic and Baltic countries in relation to the food portion of global environmental limits (%). Source: *Global Nutrition Report 2021* (17)^.^ The analysis utilises country-specific food consumption and environmental footprint data and relates them to the food portions of global environmental limits that is a global test to assess the impacts if everyone in the world consumed at the given rate. The methods and data are described in Box 1 and presented in Appendices 1–3.

It is necessary to reduce meat consumption to some degree across the Nordic and Baltic region. The current levels of red meat intake are higher than the maximum levels recommended in health-based dietary guidelines (Table 2). Figure 21 shows the variation in terms of impacts of meat consumption across the Nordic and Baltic countries and how they relate to the food portion of global environmental limits. For example, if everyone ate the same amount of chicken/pork as Lithuania it would use over 65% of the global cropland limit for food consumption. If everyone ate the same amount of beef/lamb as Iceland the threshold for GHGs from food consumption would be exceeded by 4.3 times (Fig. 21). Hence, reducing meat consumption would have considerable beneficial environmental impacts.

While the data in Table 18 suggest a shift from beef to pork or chicken would reduce GHGs, it is important to explore the realities and trade-offs of such an option. For example, shifting some beef consumption to pork and/or chicken could reduce GHGs, but increase cropland, water, and nitrogen use to produce feed crops (Fig. 21), and possibly cause further land use change, and biodiversity loss. However, the overall area of pastureland would decline due to less cattle – some of which could be restored to its native habitat cover, in turn sequestering carbon and enhancing biodiversity. Hence, depending on the priorities, such changes could shift rather than solve environmental problems. The current intake of beef, chicken, and pig meat in the region causes large impacts on land use, Greenhouse Gas Emissions (GHGE), and nitrogen and phosphorus application (Fig. 21), which suggests that a substantially reduced intake of all meats would be optimal in terms of meeting planetary health goals.

The environmental sustainability issues outlined in relation to the global context above also apply to meat production and consumption in the Nordic and Baltic region. However, there are also a number of important location-specific issues to consider. Generally, freshwater use is not currently a major problem in Nordic production because of low irrigation rates and abundant water resources. The main issues with water use relate to imported foods produced outside of the region (59). Nitrogen pollution is a major issue and is largely due to pollution from animal agriculture (manure and feed crop production). The Baltic Sea is one of the most polluted seas in the world (149) and the Oslo fjord is being increasingly polluted by nitrogen from agricultural run-off (169). This is a substantial constraint to livestock production and limits other uses of the water bodies. Limits on pollution are required (170, 171), although strict regulations of fertiliser use and manure management have resulted in large reductions in nitrogen emissions in Denmark (170, 172). A potential precedent has been recently established in the Netherlands, after the European Court of Justice ruled that the state had not taken sufficient action on reducing nitrogen pollution, and resulted in targets being identified to halve livestock numbers in order to tackle the problem (173, 174).

Although some environmental impacts of meat from farmed animals might be lower if produced in the Nordic countries compared to some countries that export to the Nordic countries, consumption of locally produced meat, assuming current consumption is maintained, does not sufficiently reduce environmental impacts, as indicated in Nordic studies on dietary patterns (47, 59, 175). Local production does have some practical benefits compared to meat imports in that the storage and transport issues are reduced which can be important for such highly perishable products.

Although in the Nordic region, livestock production on semi-natural grasslands does not always directly compete with food production for human consumption, local production of substantial amounts of crops for animal feed has a major environmental impact. This is a problem across the region and highlights the competition for feed versus food. Reconfiguring feed crops for direct human consumption (where possible) in combination with a reduction in livestock production and consumption could therefore be a method for reducing the environmental impacts of food production within the region (20–22).

Despite biodiversity issues (including those related to feed imports) being particularly important in Sweden, Finland, Norway, Iceland, and Denmark, it is not possible to adequately assess them across all countries due to a lack of comparative data. Although land use change, for example, from forest to agriculture is not a major issue in the Nordic and Baltic countries, the negative impacts of livestock production on terrestrial biodiversity are considered a serious threat (171, 176). Across the EU27 (which includes Denmark, Estonia, Finland, Latvia, Lithuania, and Sweden), livestock production has been estimated to cause 26% of total biodiversity loss (measured as mean species abundance), mostly through feed production on grasslands and croplands (171). There are limited contexts where low density livestock grazing is used to maintain semi-natural grasslands, which are linked to certain types of biodiversity and may contribute to cultural heritage, social cohesion, and recreation – particularly in Sweden, Norway, and Estonia, for example (177, 178). However, these areas are currently being threatened more by structural changes in agriculture rather than climate or environment related policies. It is important to expand current knowledge in order to assess biodiversity impacts alongside other important environmental metrics. Even with adequate data, it will still be impossible to ‘weigh’ the impacts, for example, how many units of biodiversity at the local level are equal to how many units of GHGs? Intensive animal production, in general, seems to have little or no benefit for species richness (176, 179), or to carbon sequestration (180) – but generally has lower environmental footprints in terms of land use and GHGs in comparison to extensive production. Changes in production would therefore likely be assessed using value-based judgements in relation to impacts on biodiversity, and in dealing with trade-offs against other environmental impacts.

There are also major issues related to imported feed. For example, in Norway and Sweden, the majority of imported soy, and in Finland approximately half, was certified as deforestation-free (181) in 2020, yet in the other Nordic and Baltic countries, the proportion was smaller or negligible (182) [although there are some potential issues with transparency and shifting demand due to rising costs in relation to certification schemes (183)]. In terms of reducing the impact of imported feed, the availability of lower impact feed alternatives and/or whether they can be produced in-country (rather than being imported from another country) could be important areas of inquiry if not already in progress. In Denmark, for example, farmers have begun partly replacing soy imports used for farmed animal feed with vicine-free fava beans (184–186). Similarly, in Finland, the Leg4Life initiative is operational (126).

Iceland dedicates by far the largest proportion (99%) of its agricultural land to pasture (mostly permanent), followed by Norway (67%, mostly temporary), Sweden (54%, mostly temporary), Estonia (48%, mostly permanent), Latvia (47%, mostly permanent), Finland (40%, mostly temporary), Lithuania (34%, mostly permanent), and Denmark (29%, most temporary) (Table 1). Large areas of land are used for sheep production in Iceland which causes biodiversity loss and damage to ecosystems via sheep grazing on emerging vegetation and is a particular problem in sensitive areas (187).

In Sweden, domestic production is dominated by beef from the dairy sector (64%) (188) that is from culled cows and offspring raised for meat. There has been a large reduction in dairy cows due to increasing milk yield, and these have been replaced by an increasing number of suckler cows. Most bulls are raised indoors to a slaughtering age of between 15 and 24 months on a diet of approximately 50% forage and 50% grains (188). The use of soy for feed is low in beef production and 2.5% in dairy production (which a substantial part of beef comes from) (189). 15% of slaughtered beef animals are raised under organic production methods. Suckler cows (and their calves), dairy cows and heifers graze in summer (required by Swedish animal welfare legislation). Grass-clover ley grown in rotation with other crops or in monocultures is the main feed source (47, 58).

In Denmark, the largest proportion of all beef originates from dairy systems (83%), estimated from data on the production of bone-free meat from different types of cattle in Denmark. Intensive beef accounts for 15% and extensive beef for 2%, which is mostly under organic production. Within the dairy systems, dairy cows supply the largest share of minced meat and dice/strips (55 and 63%, respectively), whereas calves supply the largest share of steaks (59%). Beef produced from dairy cows in the Nordic countries appears to result in lower environmental impacts compared to beef produced from beef cattle. Mogensenet al. (190) estimated that GHGs from beef produced in Denmark and Sweden from dairy bull calves slaughtered between 9 and 19 months old had the lowest GHGs, ranging from 9 to 12 kg CO_2_/kg of carcass weight. Comparatively, GHGs of beef from beef breed systems ranged from 23 to 30 kg CO_2_/kg carcass weight. The differences were largely attributed to differences in feed intake over the animal’s lifetime. A study of beef production in Finland (191) estimated a range of environmental impacts of (carcass weight) beef produced from dairy cows and beef cattle respectively: GHGs were 25 and 32 kg CO_2_eq/kg; aquatic eutrophication was 22 and 33 g PO_4_^3-^eq/kg; and acidification potential was 58 and 64 gAE-eq/kg. In these examples, the environmental impacts were higher for beef produced from beef cattle compared to beef produced from dairy cows, which is also apparent for GHGs and land use based on a global average of beef produced from beef herds and beef produced from dairy herds, but not for acidifying and euthrophying emissions (Table 18) (2).

In Finland, the majority of pastures are intensively farmed and are not adequately supporting biodiversity (176). It is not necessarily possible to expand agricultural land in Finland as this could result in even more peatland being brought into production (currently 8–11% of agricultural land is peatland).^29^ Reducing the demand for agricultural land through shifting to more plant-based diets would allow peatland to be taken out of production, rather than replace grass production on peatland with grain production, for example, which could increase GHG emissions (192). A reorganisation of crop rotations on the remaining agricultural land would be required to maintain or improve the carbon stock and agricultural condition of farmland (193).

In Norway, a large area of land used for temporary pasture is deemed unsuitable for crop production under current market incentives. Most (~90%) crops produced in Norway are used for farmed animal feed. This includes grass and roughage production, but also around two thirds of the grains produced are feed rather than food grains. Outdoor grazing is limited due to seasonal reasons. When indoors, cows receive a mixture of roughage and concentrates. However, recent analysis suggests that more of the cultivated land could be used for producing plant-based foods (194). An increase in vegetable production (including potatoes) is also possible, and portions of feed could be reallocated to human food (e.g. oats) (99). Structural changes have increased the size of farms, with much fewer small farms now operating. Due to smaller production and long distances, production in more marginal areas is no longer financially profitable (195). Improving incomes and maintaining viable districts with livestock production is considered a priority area in Norway, and has resulted in conflicting agricultural and environmental policy goals (196). This demonstrates some of the socio-political challenges in terms of reducing the environmental impacts of food production and consumption within the region and the importance of addressing such issues in order to progress towards more environmentally sustainable agriculture.

The overarching issue across the Nordic and Baltic region in relation to livestock production is land use. If, where and how to reduce production is likely to be considered from the perspective of value judgements which currently dominate decisions around the relative value of maintaining livestock production versus increasing biodiversity and mitigating climate change. The favouring of the latter against the former is largely dependent on whether the focus is on maintaining the status quo (i.e. current income sources, current land use patterns, current levels and types of biodiversity, current response to meeting the Paris Agreement goals), or taking a more ambitious approach to tackling climate and biodiversity issues, and aligning income sources with those goals (66).

A broader point is that current agricultural land use across the countries could usefully be examined to identify ‘best’ uses for maximising environmental sustainability outcomes. For example, fully repurposing some areas currently under permanent pasture would allow native vegetation and ecosystems (e.g. the Boreal forest or wetlands in relevant countries), to regenerate, and wildlife to be reinstated (and predator–prey relationships), providing additional benefits in terms of climate change mitigation, biodiversity, and more functional ecosystems. Any such changes to land use might require identification of alternative or replacement income sources.

#### Beef production and trade

Of the 8 countries, Sweden is the largest beef producer, but none of the countries contribute substantially to global beef production. All countries except Latvia rely on beef imports to some extent to supply national consumption (Table 19). In countries with a greater reliance on imports, there is less opportunity to influence the environmental impacts of current consumption through changing production methods. However, all countries could influence environmental impacts by reducing national beef consumption, and focusing on production from systems (home and abroad) with lower environmental impacts. Supply levels vary quite substantially across the 8 countries – with levels in Denmark more than four times those in Lithuania (Table 19). Hence, for some countries there is scope for reducing national beef consumption more significantly.

**Table 19:** Beef production and supply across the Nordic and Baltic countries in 2018 (84)

Country	Production (1,000 tonnes) (% of global total)	Imports (1,000 tonnes)	Exports (1,000 tonnes)	Balance (1,000 tonnes)	Food supply (Kg/person/year)
**Denmark**	129 (0.2)	124	107	146	23.52
**Finland**	87 (0.1)	27	4	110	19.26
**Iceland**	5 (<0.0)	1	0	6	14.89
**Norway**	89 (0.1)	13	0	102	17.66
**Sweden**	137 (0.2)	113	12	238	22.58
**Estonia**	13 (<0.0)	8	4	17	8.64
**Latvia**	17 (<0.0)	7	13	11	5.73
**Lithuania**	42 (0.1)	8	33	17	5.36

For comparison, beef consumption in line with a flexitarian diet that aligns with planetary boundaries up to 2050 (if implemented in combination with medium-ambition technological measures to reduce inputs during food production, and reductions in food loss and waste of 75%) recommends a daily maximum beef intake of 7 g, or 2.6 kg per year (as part of an average daily intake of 2,100 kcal) (14). Based on the annual supply data in Table 19, all countries exceed this amount (on a per person basis) 2–9 fold. Similarly, using red meat consumption data from Table 2 indicates that consumption in all countries far exceeds the amount consistent with the flexitarian diet.

#### Pork production and trade

Denmark accounts for 1.3% of global pig meat production and is by far the largest producer from the 8 countries. All countries except Denmark rely on pig meat imports to some extent to supply national consumption (Table 20). Hence, there could be greater potential to reduce the environmental impacts of production in Denmark compared to other countries that rely more on imports. However, all countries could influence environmental impacts by reducing national consumption, and focusing on production from systems (home and abroad) with the most transparent environmental impact data. Supply levels vary quite substantially across the 8 countries – with levels in Lithuania almost twice those in Denmark. Hence, for some countries, there is scope for reducing national consumption more significantly. For comparison, pork consumption in line with a flexitarian diet that aligns with planetary boundaries up to 2050 (if implemented in combination with medium-ambition technological measures to reduce inputs during food production, and reductions in food loss and waste of 75%) recommends a daily maximum pork intake of 7 g, or 2.6 kg per year (as part of an average daily intake of 2,100 kcal) (14). Based on the annual supply data in Table 20, all countries exceed this amount (on a per person basis) 8–19 fold. Similarly, using pig meat consumption data from Table 2 indicates that consumption in all countries far exceeds the amount consistent with the flexitarian diet, except in Iceland (although data is missing for 4 countries in Table 2).

**Table 20:** Pig meat production and supply across the Nordic and Baltic countries in 2018 (84)

Country	Production (1,000 tonnes) (% of global total)	Imports (1,000 tonnes)	Exports (1,000 tonnes)	Balance (1,000 tonnes)	Food supply (Kg/person/year)
**Denmark**	1,583 (1.3)	148	1,445	286	26.7
**Finland**	169 (0.1)	42	23	188	37.7
**Iceland**	7 (<0.0)	1	0	8	20.9
**Norway**	137 (0.1)	6	5	138	22.9
**Sweden**	249 (0.2)	111	29	331	30.9
**Estonia**	42 (<0.0)	40	28	54	37.8
**Latvia**	39 (<0.0)	58	11	86	43.6
**Lithuania**	72 (<0.0)	89	19	142	48.4

#### Chicken meat production and trade

Of the eight countries, Sweden is the largest chicken meat producer, but none of the countries contribute substantially to global chicken meat production. All countries except Denmark and Lithuania rely on chicken meat imports to some extent to supply national consumption (Table 21). Hence, there is significant potential to reduce the impacts of both production and consumption in Denmark and Lithuania. In countries with a greater reliance on imports, there is less opportunity to influence the environmental impacts of chicken production. Latvia and Estonia who import more than they produce, have least ability to influence the environmental impacts of production given that it is mostly served via imports. However, all countries could influence environmental impacts by reducing national consumption, and focusing on production from systems (home and abroad) with the most transparent environmental impact data. Supply levels vary quite substantially across the 8 countries – with levels in Lithuania almost twice those in Sweden. Hence, for some countries, there is scope for reducing national consumption more significantly and replacing where needed with more environmentally sustainable alternatives such as legumes and pulses.

**Table 21:** Poultry meat production and supply across the Nordic and Baltic countries in 2018 (84)

Country	Production (1,000 tonnes) (% of global total)	Imports (1,000 tonnes)	Exports (1,000 tonnes)	Balance (1,000 tonnes)	Food supply (Kg/person/year)
**Denmark**	156 (0.12)	141	142	155	26.9
**Finland**	135 (0.11)	17	11	141	19.4
**Iceland**	9 (0.01)	1	0	10	30.9
**Norway**	98 (0.08)	2	0	100	20.6
**Sweden**	161 (0.13)	81	26	216	16.9
**Estonia**	19 (0.02)	30	12	37	22.2
**Latvia**	34 (0.03)	47	21	60	21.0
**Lithuania**	134 (0.11)	45	66	113	29.0

For comparison, poultry meat consumption in line with a flexitarian diet that aligns with planetary boundaries up to 2050 (if implemented in combination with medium-ambition technological measures to reduce inputs during food production, and reductions in food loss and waste of 75%), recommends a daily maximum poultry meat intake of 29 g, or 10.6 kg per year (as part of an average daily intake of 2,100 kcal) (14). Based on the annual supply data in Table 21, all countries exceed this amount (on a per person basis) by around 2–3 fold. In contrast, using chicken meat consumption data from Table 2 indicates that consumption in Finland, Iceland, Lithuania, and Latvia exceeds the amount consistent with the flexitarian diet, whereas other countries consume below or close to this amount.

#### Considerations for reducing environmental impacts of meat consumption in Nordic and Baltic countries

- A reduction in meat consumption is a first order priority – with greatest scope in countries consuming above levels compliant with planetary boundaries (mostly beef and pork across all countries, but also chicken for some countries).
- If meat consumption needs to be replaced and not just reduced (e.g. to meet recommended nutrition intakes), replacement with plant-based foods such as legumes, pulses and whole grains would reduce the impacts of meat consumption as much as possible and avoid trade-offs to the greatest extent (in comparison to replacing beef with chicken or pork, for example). The environmental impacts of replacing some meat consumption with the lowest impact seafoods could be assessed, with a requirement to avoid problem shifting.
- Reducing meat production and consumption would reduce the demand for feed crops (see related considerations in sections 3 and 5 for cereals and pulses/legumes); hence, replacing meat with plant-based foods would not require an increase in cropland (rather a reconfiguration of crop production to best suit human needs).
- The increased use of locally grown legumes as food could give incentives for farmers to include legumes in crop rotations, which in turn would reduce the requirements for synthetic nitrogen fertilisers and improve soil quality. Other plant-based meat replacement products might also be suitable alternatives – however, this would need to be assessed depending on type and availability. Potential food shifts will be discussed further in the next paper of this series.
- Changing elements of production such as replacing feed with legally permitted and safe by-products and shifting to more extensive methods might offer benefits in some contexts. However, such options do not negate the primary need to reduce meat consumption substantially. For example, shifting all beef production to extensive methods would require a reduction in overall beef production levels to avoid increasing GHG emissions and land use (21).
- Given the range of factors involved and the complexity of trade-offs and location specific impacts and contexts, a national strategy would be important to usefully direct the reduction and replacement of meat production and consumption. An important part of this would be a comprehensive review to identify and explore the relevant range of issues including human health and social impacts, animal welfare, and current and emerging threats, for example, zoonotic diseases (and the risks of emerging zoonoses with potential pandemic consequences), and environmental change such as temperature extremes.
- Overarching, and in parallel, a national land use assessment could inform optimal land uses for meeting a range of environmental goals, also accounting for the environmental impacts of food imports in producer countries. One important inclusion would be an assessment of different types of pasture lands in terms of their current value and necessity for food production versus alternatives uses, such as restoring native habitats to help meet other social goals (i.e. meeting climate and biodiversity goals). For example, on native grasslands, improved grazing management could partially restore vegetation and related carbon stores but would come with a trade-off of non-CO_2_ GHGs from the grazing ruminants. Fully restoring pasture land to native forest cover (where relevant) removes this trade off, and could have large scale potential to sequester more carbon and provide habitats for a wider range of wildlife across the region (43, 197).
- Ensuring a trajectory within planetary boundaries requires a longer-term perspective on the role of livestock within the food system rather than focussing only on the realities of today’s market and other immediate socio-political determinants. Situations can change rapidly and business as usual cannot be assumed under the expected impacts of climate change alone.

## NNR Food Group 9: Milk and dairy products

### Global context

At the global level, 876 mt milk (excluding butter) were produced in 2019 of which 3% were used for farmed animal feed. The majority was produced from cows (81%), followed by buffalo (15%), goat (2%), sheep (1%), and camel (<1%). India produces by far the largest quantity of milk (187 mt in 2019), followed by the US (99 mt), Pakistan (56 mt), China (36 mt), and Brazil (35 mt) – making up the top 5 producers globally (83).

**Fig. 22:**
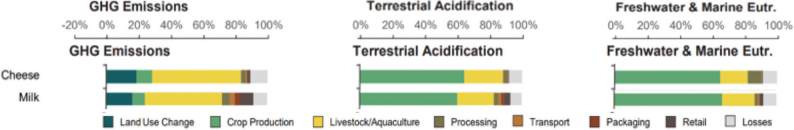
Proportion of environmental impacts from different life cycle stages: Milk and dairy products (excl. butter) (global) (2).

Collectively, dairy production has a large environmental footprint at the global level, accounting for 4% of total anthropogenic GHGs, for example (92). This is largely a result of the use of ruminant animals for the majority of milk production (cows, buffalo, goats, and sheep). The environmental impacts of dairy products tend to be concentrated during the production stages of the life cycle, which account for around 80% of impacts. For example, in terms of GHGs, the animal farming component accounts for the largest share of emissions as a result of methane and nitrous oxide released from the ruminant animals and from their manure, whereas acidification and eutrophication impacts are largely attributed to feed crop production (Fig. 22).

Cheese has a much higher impact compared to dairy milk as it is a concentrated form of milk, requiring around 10 litres of milk to produce 1 kg of cheese (Table 22). The impacts of butter are not described here, but animal fat is included in section 11 on fats and oils.

**Table 22:** Average environmental impacts per kg of retail weight: Milk and dairy products (global) (2)

	Land use (m^2^/kg)	Greenhouse gas emissions (kg CO_2_eq/kg)	Acidifying emissions (g SO_2_eq/kg)	Eutrophying emissions (g PO_4_^3-^eq/kg)	Freshwater (L/kg)
**Milk**	9.0	3.2	20.0	10.7	628
**Cheese**	87.8	23.9	165.5	98.4	5,605

Many of the same issues discussed in chapter 8 related to meat production (e.g. feed production, deforestation, large land use, manure and urea, nutrient pollution of surrounding ecosystem, resource use on farm including water and energy for housing, and culling of local wildlife), apply to dairy production. Additionally, for dairy products, animal welfare and antibiotic use are also important issues to consider (198), but are outside the scope of the current paper.

As for the impacts of meat production, the level of impacts per unit of milk production varies depending on a number of factors including milk yield (which is not uniform across the global herd of farmed cows), and feed type, production methods, and production location. For example, locally grown feed produced by nitrogen fixing legumes will potentially have lower environmental impacts compared to imported grains. CH_4_ emissions from ruminant animals can also vary depending on feed type and its digestibility (higher cellulose feeds result in higher CH_4_ release from the animals), and emissions from manure can also vary, for example, depending on temperature, amounts, oxygen availability, and storage type (75). How environmental impacts are reported per unit of milk are also dependent on the allocation method used in the life cycle assessment. For example, if economic allocations are applied, a greater proportion of environmental impacts over a cow’s life will be attributed to meat production rather than milk production. However, on a weight basis, most of the environmental impact would be allocated to milk production (13).

### Nordic and Baltic context

The Nordic and Baltic countries have some of the highest levels of dairy milk consumption in the world – for example, Finland currently has the second highest global consumption rates, with Estonia having the 6th highest, Denmark the 9th highest, and Iceland the 18th highest (84). Environmental impacts of dairy consumption are also high across the countries, making up a substantial proportion of total impacts from all food consumption – particularly in relation to GHGs and land use, but phosphorus and nitrogen are also significant. Reducing dairy consumption is the most effective way to reduce environmental impacts from this food group and will be necessary to align with food-related global environmental limits.

**Fig. 23:**
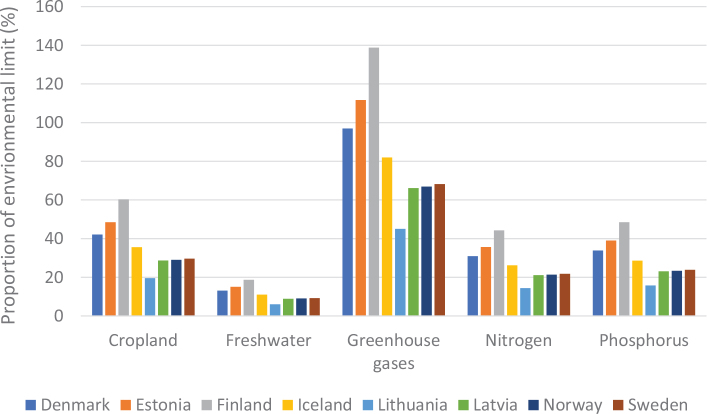
Impacts of milk consumption across the Nordic and Baltic countries in relation to the food portion of global environmental limits (%). Source: *Global Nutrition Report 2021 (**17**).* The analysis utilises country-specific food consumption and environmental footprint data and relates them to the food portions of global environmental limits that is a global test to assess the impacts if everyone in the world consumed at the given rate. The methods and data are described in Box 1 and presented in Appendices 1–3.

Figure 23 shows the variation in environmental impacts of dairy milk consumption across the Nordic and Baltic countries and how they relate to the food portion of global limits. For example, if everyone in the world consumed the same amount of dairy as each person in Finland, the global GHG limit for food consumption would be exceeded by almost 1.4 times and would use 60% of the cropland limit for food consumption.

Many of the issues covered in the global context regarding the environmental impacts of dairy are also relevant to the Nordic and Baltic countries. There are some regional and local variations; for example, some regions are more dependent on feed imports (e.g. rapeseed cake in Finland) due to shorter grazing seasons and less local production of feed crops. Dairy cows are generally fed a mixture of roughage (silage, hay, pasture, and maize) and various types of concentrates derived from crops such as cereals, rapeseed, soya bean, beet pulp, and molasses. The impacts of feed production vary depending on which crops are used, and where they are sourced from – for example, regulations on plant crop protection varies across the world and therefore soybeans imported from Brazil could have different impacts compared to locally produced pulses. In Sweden, soybean use for dairy cows is approximately 2.5% of the feed (189). The amount of soy that Norway imports for animal feed would cover more than 50% of the Norwegian population’s basic protein needs (199). Production methods are also important; for example, intensively grown animal feed can result in soil erosion within the region (see chapters 3 and 5) (20).

Intensively produced dairy has a range of environmental issues across the region. Generally, dairy farms have become fewer in number and larger in size, which concentrates certain impacts in the local environment, such as nutrient pollution. Farm location also impacts energy use – for example, areas with shorter grazing seasons will need to use relatively more energy for lighting and ventilation for animal housing (75).

Some variation exists in terms of environmental impacts in comparison to the global average of 3.2 kg CO_2_eq/kg retail weight in Table 22. For example, a Swedish study (200) estimated the impact for energy corrected milk (ECM), including surplus calves and culled cows, to be 1.16 kg CO_2_e/kg ECM in Sweden, similar to an estimate of milk in Denmark (1.06 kg CO_2_e/kg ECM) (201). However, the amount used in Fig. 23 to estimate the GHG impact of milk consumption across the Nordic and Baltic countries in relation to planetary boundaries is 1.39 kg CO_2_eq/kg (see Appendix 2). The main route for reducing the environmental impacts of dairy production is a reduction in production amounts, to reduce absolute impacts. The main route for reducing GHGs per unit of production (relative impacts) for both organic and non-organic is increasing milk yield (75). However, this is already very high and will not reduce the absolute impact of dairy production if production levels increase with increasing demand. The impact of plant protection products can vary the impacts of organic relative to non-organic production. For example, some chemical products can be used in conventional milk production via feed crops, but are not permitted in organic crop production (although small amounts of non-organic feed might be permitted for organic dairy production in some countries such as Sweden) (75). Using a larger proportion of locally and regionally produced organic feeds such as grains, oil seeds, and pulses, some feed from pastures under extensive production (20), and potentially low-opportunity-biomass (i.e. biomass not in competition with human food) (202) could help to reduce the impacts of dairy per unit of production; however, this does not fully address a number of important issues including the competition for agricultural land (e.g. feed versus food), and the need for overall dairy consumption levels to decline across the region.

Denmark is by far the largest producer of dairy milk, followed by Sweden and Finland. Denmark is also the largest net exporter of dairy milk, followed by Estonia and Latvia. All other countries, except Iceland, are net importers of dairy milk. Finland is the largest dairy milk consumer in the region on a per person basis, followed by Estonia and Denmark. Lithuania has the lowest consumption levels (Table 23). Milk from cows accounted for by far the majority of production and consumption in all countries.

**Table 23:** Milk (excluding butter) production and supply across the Nordic and Baltic countries in 2019 (84)

Country	Production (1,000 tonnes)	Imports (1,000 tonnes)	Exports (1,000 tonnes)	Balance (1,000 tonnes)	Food supply (Kg/person/year)
**Denmark**	5,615	934	3,148	3,401	238
**Estonia**	822	81	376	527	278
**Finland**	2,374	759	209	2,924	336
**Iceland**	156	1	7	150	203
**Latvia**	981	260	554	687	165
**Lithuania**	1,551	657	629	1,579	108
**Norway**	1,573	131	90	1,614	166
**Sweden**	2,704	998	340	3,362	182

For comparison, milk consumption in line with a flexitarian diet that aligns with planetary boundaries up to 2050 (if implemented in combination with medium-ambition technological measures to reduce inputs during food production, and reductions in food loss and waste of 75%) recommends a daily maximum milk intake of 250 g, or 91.3 kg per year (as part of an average daily intake of 2,100 kcal) (14). Based on the annual supply data in Table 23, all countries exceed this amount (on a per person basis), with Lithuania being closest with an average daily consumption of 296 g. Average supply in Finland exceeds the amount by 3.7 times, followed by Estonia (3 times), Denmark (2.6 times), Iceland (2.2 times), Sweden (2 times), Norway (1.8 times), and Latvia (1.8 times). In contrast, using milk and cheese consumption data from food diaries in Table 2, consumption in all countries exceeds the amount consistent with the flexitarian diet, except for Latvia and Lithuania.

#### Considerations for reducing environmental impacts of milk consumption in Nordic and Baltic countries

- Reducing dairy milk consumption, especially cheese, would provide the greatest environmental benefits across a range of metrics (e.g. agricultural land use, GHGs, nutrient pollution, biodiversity loss). Hence, a reduction in consumption should be considered a first order priority – with greatest scope in countries consuming above levels compliant with planetary boundaries. Currently, dairy cows are used to supply milk during their lives and beef meat at slaughter. Therefore, for maximum effect, a reduction in dairy consumption should be paired with a reduction in beef consumption – otherwise, beef cattle herds (which have higher environmental impacts per unit of beef compared to beef produced from dairy herds) might be increased to compensate for a reduced beef output from the dairy industry.
- If a replacement of nutrition is required (rather than just a reduction), this should also be considered in terms of opportunities to minimise environmental impacts through replacement with plant-based alternatives where possible.
- Reducing dairy production and consumption would reduce the demand for feed crops (see related considerations in chapters 3 and 5 for cereals and pulses/legumes); hence, replacing dairy milk with plant foods may not require an increase in cropland.
- Similarly for meat production and consumption, given the range of factors involved and the complexity of trade-offs and location specific impacts and contexts, a national strategy should usefully direct the change in milk production and consumption. An important part of this would be a comprehensive review to identify and explore the range of issues including biodiversity, human health and social impacts, animal welfare, and current and emerging threats, for example, antibiotics, where necessary, and zoonotic-driven pandemics.
- The approach to reducing dairy production and consumption must be done in conjunction across all livestock production and consumption given the important connections, interdependencies and similarities between meat and dairy, and must take a longer-term perspective rather than being limited to the realities of today’s market and other socio-political determinants. Situations can change rapidly, and business as usual cannot be assumed under the expected impacts of climate change alone.

## NNR Food Group 10: Eggs

### Global context

At the global level, 90 mt eggs were produced in 2019 of which 93% were from chickens. China produced 37% of the global total (37 mt in 2019), followed by the US (7 mt), Indonesia (5 mt), India (5 mt), and Brazil (3 mt) – making up the top 5 producers globally (84).

**Fig. 24:**
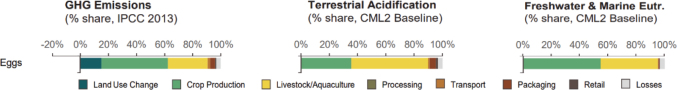
Proportion of environmental impacts from different life cycle stages: Eggs (global) (2)

The environmental impacts of eggs tend to be largely concentrated during the production stages of the life cycle, which account for around 90% of impacts. For example, GHGs are largely attributed to feed crop production, whereas the animal farming component accounts for the largest share of acidification impacts, and eutrophication impacts are more equally spread between feed crop production and animal farming (Fig. 24). Table 24 shows a range of environmental impacts per kg of egg production as a global average.

**Table 24:** Average environmental impacts per kg of retail weight: Eggs (global) (2)

	Land use (m^2^/kg)	Greenhouse gas emissions (kg CO_2_eq/kg)	Acidifying emissions (g SO_2_eq/kg)	Eutrophying emissions (g PO_4_^3-^eq/kg)	Freshwater (L/kg)
**Eggs**	6.3	4.7	53.7	21.8	578

The types of environmental issues related to egg production are largely the same as those related to meat and dairy production (e.g. land use, manure and urea, nutrient pollution of surrounding ecosystem, resource use on farm including water and energy for housing) (see chapters 8 and 9), which includes a significant proportion of impacts from feed production (see chapters 3 and 5 for more information on the environmental impacts of cereals and legumes). Antibiotic use, pathogen spread, and animal welfare are also relevant to egg production, but are beyond the scope of the current paper.

#### Nordic and Baltic context

**Fig. 25:**
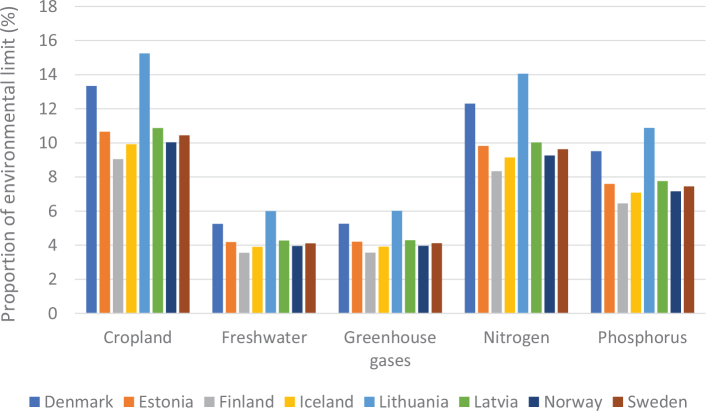
Impacts of egg consumption across the Nordic and Baltic countries in relation to the food portion of global environmental limits (%) Source: *Global Nutrition Report 2021 (**17**)* The analysis utilises country-specific food consumption and environmental footprint data and relates them to the food portions of global environmental limits that is a global test to assess the impacts if everyone in the world consumed at the given rate. The methods and data are described in Box 1 and presented in Appendices 1–3.

Figure 25 shows the variation in environmental impacts of egg consumption across the Nordic and Baltic countries and how they relate to global limits. For example, if everyone in the world had the same per person egg consumption as Lithuania, it would use 15% of the global cropland limit for food consumption and 14% of the nitrogen limit for food consumption.

While the environmental impacts of egg consumption might appear insignificant or relatively small compared to other animal products such as meat and dairy, the environmental impacts of egg consumption exceed the impacts of other important food groups in a number of ways within the region. For example, egg consumption in Denmark has a greater environmental impact in terms of GHGs, cropland, water, nitrogen, and phosphorus than the consumption of root vegetables. In Estonia, egg consumption requires more cropland than legumes and roots combined. Egg consumption in Sweden requires more nitrogen than fruits and vegetables, and more land than the consumption of legumes and nuts combined. In Lithuania, egg consumption uses more cropland and emits more GHGs than those related to fruit and vegetable consumption. More freshwater is used for egg consumption in Iceland compared to legumes and nuts combined (Figs. 10 and 29).

**Fig. 26:**
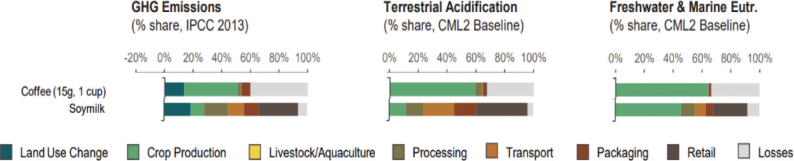
Proportion of environmental impacts from different life cycle stages: Coffee and soy milk (global) (2).

Sweden is the biggest producer of eggs, followed by Denmark and Finland. Denmark, Sweden, and Lativa have the largest supply of eggs, followed by Estonia. Finland and Latvia are net exporters of eggs, while Denmark is the largest net importer followed by Estonia (Table 25). For comparison, egg consumption in line with a flexitarian diet that aligns with planetary boundaries up to 2050 (if implemented in combination with medium-ambition technological measures to reduce inputs during food production, and reductions in food loss and waste of 75%) recommends a daily maximum egg intake of 13 g, or 4.7 kg per year (as part of an average daily intake of 2,100 kcal) (14). Based on the annual supply data in Table 25, all countries exceed this amount (on a per person basis), by 2–3 times. In contrast, using egg consumption data from food diaries in Table 2, Sweden is the only slightly above the flexitarian diet recommendations – consumption in all other countries exceeds this amount.

**Table 25:** Egg production, trade and supply across the Nordic and Baltic countries in 2019 (84)

Country	Production (1,000 tonnes)	Imports (1,000 tonnes)	Exports (1,000 tonnes)	Balance (1,000 tonnes)	Food supply (Kg/person/year)
**Denmark**	78	40	23	95	14
**Estonia**	9	11	2	18	13
**Finland**	76	3	12	67	11
**Iceland**	5	0	0	5	12
**Latvia**	46	16	31	31	14
**Lithuania**	48	18	21	45	12
**Norway**	71	1	0	72	12
**Sweden**	143	18	20	141	14

#### Considerations for reducing environmental impacts of egg consumption in Nordic and Baltic countries

Egg consumption in all countries exceeds amounts consistent with planetary boundaries. While the environmental impacts of egg consumption might appear insignificant or relatively small compared to other animal products such as meat and dairy, they should be considered within the context of the amount of nutrients provided to diets via egg consumption, and the potential to instead at least partly achieve such nutrition from other less environmentally burdensome sources as a contribution to reducing the overall environmental impacts of nutritionally adequate diets and the food system in line with key environmental targets.

## Section 3: Additional food groups to consider

### NNR Food Group 1: Breastfeeding

While breastfeeding is not a major area of concern regarding the environmental impacts of food consumption across the Nordic and Baltic countries, there is an emerging literature that demonstrates some considerations in terms of reducing environmental impacts. The majority of breast milk substitutes are based on bovine milk (from cows, which are ruminant animals) and environmental impacts occur largely from this ingredient. For example, breast milk substitutes based on bovine milk assessed in 4 different countries (UK, China, Brazil, and Vietnam) all revealed the majority (68–82%) of the GHGs impact of production arise from raw milk. The remaining emissions arise from other ingredients including lactose or glucose syrup, vitamins and minerals, and a blend of vegetable oils; and transport, production and in-home sterilisation of bottles, and preparation of breast milk substitutes. In comparison, in all four countries the GHG impact of breastfeeding was 40%, 53%, 43%, and 46% lower than breast milk substitutes (203). Similarly, a recent study of Norwegian consumption (204) found that 4 months exclusive feeding with infant formula had an environmental impact of 35–72% higher than 4 months exclusive breastfeeding, depending on the impact category which included global warming potential, terrestrial acidification, marine and freshwater eutrophication, and land use. Bovine milk was the main source of environmental impact across all categories, for the infant formula. The environmental impact of breastfeeding was based on the additional food intake required for breastmilk production in lactating mothers. Additionally, a recent analysis of UK consumption estimated that breastfeeding for six months could save 95–153 kg CO_2_ equivalents per baby compared with breast milk substitutes based on bovine milk – equivalent to GHG emissions from 50,000 and 77,500 cars each year in the UK (205). While it is possible to reduce the environmental impacts of breast milk substitutes, it has been demonstrated that for both Ireland and China, the GHG emissions saved by achieving the minimum 50% Exclusive Breast Feeding target are greater than decarbonising current consumption (including breast milk substitutes) with renewable energy alone (206).

Dairy milk production has a substantial environmental burden in terms of GHGs, land use, nitrogen use, phosphorus impacts, freshwater use, and biodiversity loss, and is explored in detail in ‘NNR Food Group 9: Milk and dairy products’ (see chapter 9).

## NNR Food Group 2: Drinks

The drinks group includes coffee, tea, sugar sweetened and artificially sweetened drinks. However, sugar is covered in ‘NNR Food Group 12: Sweets and confectioneries’ (see chapter 12); therefore, this chapter will focus on tea and coffee, and plant-based drinks.

### Global context

Globally, in 2019 10 million tonnes (mt) of coffee and 7 mt of tea were produced. Brazil produced the largest quantity of coffee in 2019 (3 mt), followed by Vietnam (1.6 mt) and Columbia (0.9 mt). Tea production is concentrated in east and south Asia, with China producing the largest amount in 2019 (2.8 mt), followed by India (1.4 mt) (83).

The creation of tea plantations typically displaces native forest and results in biodiversity loss (207).

Globally, coffee production was estimated to be the sixth largest driver of deforestation – and thus biodiversity loss – from 2001 to 2015, contributing a slightly smaller share than cocoa (see chapter 12), and around 24 times less deforestation in comparison to cattle (116).

The land use change impacts are reflected in the proportion of environmental impacts across different stages of the life cycle, with more than 10% of the GHGs related to coffee resulting from land use change. Crop production, that is on farm, is generally the most important life cycle stage in terms of environmental impacts; however, coffee has relatively large impacts associated with losses (in comparison to other food products which typically have less than 20% of life cycle impacts from losses e.g. cereals). The impacts of soy milk tend to be more spread across the stages, with retail having the largest proportion of GHGs and acidification impacts, and crop production causing the largest share of eutrophication impacts (Fig. 26).

**Fig. 27:**
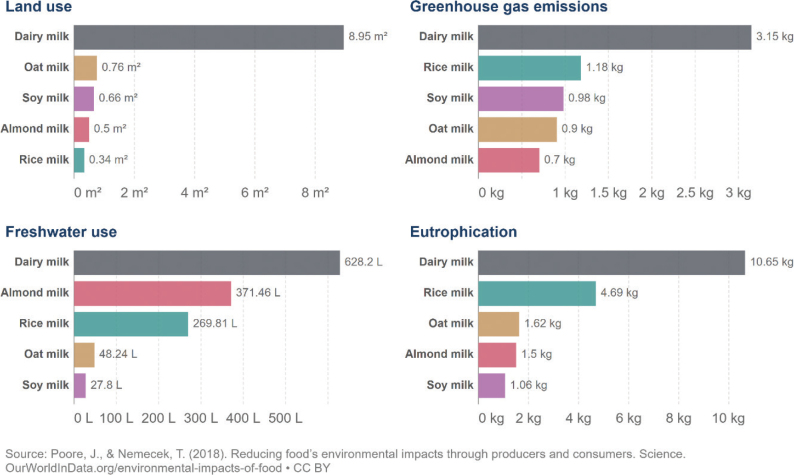
Environmental impacts of plant-based drinks, per litre (213).^30^

Table 26 shows a range of impacts related to coffee and soy milk production. Note that the amounts given are per kg of retail weight, and a cup of coffee typically requires 15 g of coffee; hence, the amounts shown represent coffee for around 67 cups. In that case, 1 cup of coffee would, for example, result in 0.4 kg CO_2_eq, in addition to the resources used for water (e.g. energy for boiling), milk, sweetener, and packaging (such as disposable cups or aluminium foil for individual portions for coffee machines). Per cup of tea, the GHG impact has been estimated to be up to 0.2 kg CO_2_ depending on how it is grown, processed, shipped, packed, brewed, and discarded. Loose leaf tea has a smaller impact (around 10 times less) due to a reduced need for packaging. Boiling water is one of the highest energy-consuming aspects of tea beverages (207).

**Table 26:** Average environmental impacts per kg of retail weight: Coffee and soy milk (global) (2)

	Land use (m^2^/kg)	Greenhouse gas emissions (kg CO_2_eq/kg)	Acidifying emissions (g SO_2_eq/kg)	Eutrophying emissions (g PO_4_^3-^eq/kg)	Freshwater (L/kg)
**Coffee**	21.6	28.5	83.1	110.5	26
**Soy milk**	0.7	1.0	2.6	1.1	28

Figure 27 shows environmental impacts of a range of plant-based drinks, with dairy milk included as such drinks tend to be used in the same way, and plant-based milk or plant-based dairy alternatives are mentioned in many FBDGs (208). An increased demand for such alternatives is expected over the next decade and lactose malabsorption is highly prevalent in the global population (including 28% of the population in western, southern, and northern Europe), making it difficult to digest cow’s milk (209). In terms of relative environmental impacts, all plant-based drinks have lower impacts than dairy milk across the range of metrics (Fig. 27). However, the nutritional values can differ significantly between dairy and plant-based in terms of saturated fat and cholesterol, and also calcium and protein depending on whether they are supplemented with nutrients. An analysis of 399 products (semi-skimmed cow’s milk and soy-, oat-, almond-, coconut-, and rice drink) across 6 European countries (including Sweden) found that 50% of the regular plant-based drinks were fortified with calcium, whereas the organic plant-based drinks were mostly unfortified. Soy drink had the best protein quality to carbon footprint ratio, followed by cow’s milk (210). However, it is important to note that fortification of dairy cow feed is common practice, in amounts similar to that of plant-based dairy alternatives (~140 mg of minerals and 1 mg vitamin per 100 g milk). The main difference between the fortified plant-based dairy alternatives and dairy milk is in protein content (211), which is unlikely to be a concern in most developed/high income country settings where protein is consumed well above the recommended level (212).

**Fig. 28:**
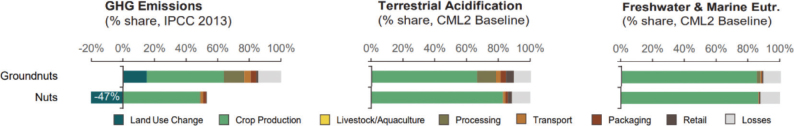
Proportion of environmental impacts from different life cycle stages: Nuts (global) (2).

Many of the same issues related to soy milk, tea, and coffee production (e.g. land use, land use change, freshwater use, nutrient pollution of surrounding ecosystems, monocultures, intensive farming methods, soil erosion, use of crop protection products, and biodiversity loss), apply to cereal, fruit, and legume production and are therefore covered in detail in the relative chapters (see chapters 3, 4, and 5 for more details). An additional issue related to coffee production is a partial proportion (up to 40%) of the yield being dependent on pollinators (214).

### Nordic and Baltic context

All countries are entirely dependent on tea and coffee imports from tropical areas for national consumption (Tables 27 and 28). In all countries, coffee is consumed in much higher quantities in comparison to tea – in some cases substantially more; for example, 59 and 41 times more coffee is consumed compared to tea in Finland and Norway, respectively. Globally, Finland, Sweden, Iceland, Norway, and Denmark have the 3rd, 4th, 5th, 6th, and 8th highest coffee consumption respectively.

In terms of plant-based drink products on the Danish market, recent testing revealed the protein content is generally low, except for soy drinks, and the vitamin and mineral content such as calcium may also be low if not fortified (215). This difference might be important for consumers with poor dietary diversity, or a reliance on dairy milk for protein intake, for example [although this is unlikely in a developed/high income country setting where protein is generally consumed above recommended levels (212)].

**Table 27:** Coffee production, trade and supply across the Nordic and Baltic countries in 2019 (84)

Country	Production (1,000 tonnes)	Imports (1,000 tonnes)	Exports (1,000 tonnes)	Supply (Kg/person/Year)
**Denmark**	0	50	8	7.3
**Estonia**	0	8	1	4.7
**Finland**	0	91	10	11.8
**Iceland**	0	3	0	8.9
**Latvia**	0	12	6	2.8
**Lithuania**	0	24	11	4.9
**Norway**	0	45	1	8.2
**Sweden**	0	135	34	10.1

**Table 28:** Tea production, trade and supply across the Nordic and Baltic countries in 2019 (84).

Country	Production (1,000 tonnes)	Imports (1,000 tonnes)	Exports (1,000 tonnes)	Balance (1,000 tonnes)	Supply (Kg/person/Year)
**Denmark**	0	6	1	-	0.7
**Estonia**	0	0	0	-	0.3
**Finland**	0	1	0	-	0.2
**Iceland**	0	0	0	-	0.6
**Latvia**	0	1	0	-	0.3
**Lithuania**	0	1	0	-	0.3
**Norway**	0	1	0	-	0.2
**Sweden**	0	5	1	-	0.4

#### Considerations for reducing environmental impacts of drinks consumption in Nordic and Baltic countries

While tea and coffee consumption is not a major area in terms of potential to reduce environmental impacts from food consumption within the Nordic and Baltic countries, there are some aspects to consider. For example, coffee consumption could be reduced without a need for replacement, to amounts that correlate positively with health. In addition, herbal teas sourced from plants grown within the region (and possibly considered weeds, such as nettles) could be alternatives to imported tea. However, herbal teas do not contain caffeine and therefore are not a functional replacement for caffeinated drinks. Reducing the environmental impacts of other ingredients added to coffee and tea, such as dairy milk, could also be explored – for example, the replacement of dairy milk with fortified plant-based drinks (see chapter 9 for considerations on reducing the impacts of dairy consumption). There are a number of measures to take regarding energy consumption of boiling water, for example, using renewable energy sources and more energy efficient equipment.

## NNR Food Group 6: Nuts

### Global context

At the global level, 131 mt nuts were produced in 2019 of which 48% were coconuts, and 38% were groundnuts (peanuts). Around 1% of total groundnut production was used for farmed animal feed. Cashews and almonds each accounted for ~3% of global production, and walnuts and chestnuts each accounted for ~2%. Combined, Indonesia, Philippines, and India produced 67% of all coconuts, and China produced the largest quantity of groundnuts (37% of the global total). The US produced the most almonds and India produced the most cashews. China was the main producer of walnuts and chestnuts (83).

The environmental impacts of tree nuts and groundnuts tend to be largely concentrated during the production stages of the life cycle, particularly in terms of eutrophication for which crop production accounts for around 80% of impacts. Tree nuts have a large carbon sequestration effect through the process of photosynthesis, resulting in a net negative land use change impact in terms of GHGs, whereas groundnuts have net positive GHG emissions related to land use change for crop production (Fig. 28).

**Fig. 29:**
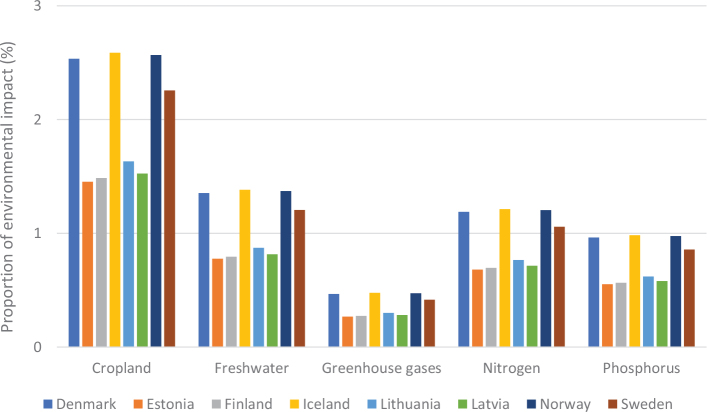
Impacts of nut and seed consumption across the Nordic and Baltic countries in relation to the food portion of global environmental limits (%). Source: *Global Nutrition Report 2021 (**17**) .* The analysis utilises country-specific food consumption and environmental footprint data and relates them to the food portions of global environmental limits that is a global test to assess the impacts if everyone in the world consumed at the given rate. The methods and data are described in Box 1 and presented in Appendices 1–3.

Table 29 shows a range of environmental impacts per kg of groundnut and tree nut production, as a global average. Groundnuts generally have lower impacts compared to tree nuts, except for GHGs. Groundnuts are a leguminous crop and fix nitrogen during production; hence, the need for nitrogen fertiliser is reduced. This again highlights the importance of trade-offs regarding environmental impacts – for example, selecting to increase the production of groundnuts rather than tree nuts on the basis of lower land use, acidifying emissions, eutrophying impacts, and freshwater use would increase GHGs and result in further land use change for crop production, in turn adversely impacting biodiversity (which is not indicated in Table 29). In addition to being important for carbon storage, maintaining and increasing biodiversity (depending on pesticide regimes and scale of monoculture), trees also help to regulate weather across large areas and can therefore be important from a micro-climate perspective, for example, rainfall and floods. Hence, there are important factors to consider beyond the utility of LCA metrics in determining the relative costs and benefits of increasing tree nut and groundnut production.

**Table 29:** Average environmental impacts per kg of retail weight: Nuts (global) (2)

	Land use (m^2^/kg)	Greenhouse gas emissions (kg CO_2_eq/kg)	Acidifying emissions (g SO_2_eq/kg)	Eutrophying emissions (g PO_4_^3-^eq/kg)	Freshwater (L/kg)
**Nuts**	13.0	0.4	45.2	19.2	4,134
**Groundnuts**	9.1	3.2	22.6	14.1	1,852

Many of the same issues related to nut production (e.g. land use change, nutrient pollution of surrounding ecosystems, monocultures, use of crop protection products, biodiversity loss), apply to cereal and legume production (see chapters 3 and 5 for more details). Additionally for groundnuts, aflatoxins (produced by fungus *Aspergillus flavus*) could be an increasing problem due to more frequent weather extremes (flood and drought).

### Nordic and Baltic context

Figure 29 shows the variation in environmental impacts of nut and seed consumption across the Nordic and Baltic countries and how they relate to the food portion of global limits (as nuts consumed are imported, the majority of impacts occur outside of the region). GHGs from nut and seed consumption tend to account for the least impact on planetary boundaries, with cropland being the biggest impact, followed by freshwater and nitrogen use (biodiversity impacts are not included in the LCA data presented here). As nut consumption is recommended to increase across the Nordic and Baltic countries, minimising such impacts per unit of production would be beneficial in order to reduce environmental burdens as consumption rises. Precision agriculture, a method used to apply inputs such as water and fertilisers in a direct rather than diffuse way in appropriate amounts at the correct times, could be important for minimising nitrogen and water use, and agroforestry could be important in terms of land use as the impacts could be spread across different crops and potential leisure activities. While Table 29 shows environmental impacts are generally lower for groundnuts in comparison to tree nuts, there is potential to grow tree nuts across the region (and possibly groundnuts in sunnier areas with some protection). Large areas of the region were covered in native Boreal forest; hence, there is potential for tree growth in conjunction with other food group changes. For example, reconfiguring portions of permanent or temporary pastureland that displaced forest, with tree nut production – and reconfiguring portions of animal feed crop land with groundnuts. Increasing tree nut production could also make important contributions to climate (in terms of carbon sequestration) and biodiversity goals. Hence, increasing the consumption of nuts should be considered in conjunction with reduced environmental impacts from shifts in other food groups, rather than being considered as an increase in the overall environmental burden of diets.

All countries are currently fully reliant on imports for nut consumption. In all countries, nut consumption is dominated by tree nuts, except for Finland where consumption is equally shared with groundnuts. The lowest proportion of groundnuts in relation to total nut intake is in Estonia, where it accounts for 13%. In Latvia, groundnuts account for 30% of total nut consumption, 32% in Denmark, 33% in Iceland, 38% in both Lithuania and Sweden, and 43% in Norway (84). For comparison, nut and seed consumption in line with a flexitarian diet that aligns with planetary boundaries up to 2050 (if implemented in combination with medium-ambition technological measures to reduce inputs during food production, and reductions in food loss and waste of 75%), recommends a daily minimum nut and seed intake of 50 g, or 18.3 kg per year (as part of an average daily intake of 2,100 kcal) (14). Average supply of tree nuts and groundnuts shown in Table 30 falls far short in all 8 countries. Based on consumption amounts for nuts in Table 2, consumption is below the amounts by at least 6-fold in all countries (there is no data on nut consumption for Lithuania in Table 2).

**Table 30:** Tree nut and groundnut production and supply across the Nordic and Baltic countries in 2019 (84)

Country	Production (1,000 tonnes)	Imports (1,000 tonnes)	Exports (1,000 tonnes)	Balance (1,000 tonnes)	Food supply (Kg/person/year)
**Denmark**	0	34	6	28	4.2
**Estonia**	0	5	0	5	3.0
**Finland**	0	14	0	14	2.3
**Iceland**	0	1	0	1	2.8
**Latvia**	0	6	2	4	2.2
**Lithuania**	0	15	7	8	2.7
**Norway**	0	21	1	20	3.3
**Sweden**	0	40	5	35	3.2

#### Considerations for reducing environmental impacts of nut consumption in Nordic and Baltic countries

As a substantial increase in nut consumption would be required across the region to meet current dietary guidelines (Table 2), or to align with recommended intakes in the Planetary Health Diet, it is important to consider the environmental impacts in an absolute and relative sense. The main goal is to reduce the relative environmental impacts of nuts per unit of production while also reducing the absolute impacts of diets in terms of total food consumption, by reducing environmental impacts through shifts in other food groups that have higher environmental impacts such as meat and dairy. We identify the following considerations in relation to the environmental impacts of increasing nut consumption:

- In a relative sense, the environmental impacts of nut production per unit of output could be reduced through the use of precision agriculture, polycultures, and organic production methods (or similar), for example, to reduce the impacts of crop protection products and provide more opportunities for biodiversity. However, the overall, or absolute impacts of nuts in the diet might still increase due to an increase in consumption.
- Reducing the disproportionately sized environmental impacts of animal products (particularly meat and dairy) will be important in reducing the total, or absolute, environmental impacts of diets, and therefore in creating ‘space’ for increased nut consumption. The potential to shift some animal product consumption to nuts could also be explored.
- The potential to increase nut production (such as hazelnuts and acorns) within the region could be included in a land use assessment, and also considered in relation to major environmental goals such as climate – particularly in relation to carbon sequestration benefits of tree nut production, and biodiversity, and also in reducing nutrient pollution to ecosystems such as the Baltic Sea. Hazelnuts have a legacy in the region and could therefore provide a useful starting point. A land use assessment would bring a comprehensive perspective, which is important for avoiding increasing total environmental loads through increased overall agricultural production, in comparison to reducing overall environmental loads through land use reconfiguration.
- Increasing nut production within the region could help to reduce import dependency, and in turn the potential insecurity of imports due to environmental issues such as increased aflatoxin in production areas more prone to weather extremes (e.g. Africa), and water stress (e.g. China).
- Other options might be useful to explore; for example, rather than increasing nut consumption to the full extent in line with dietary guidelines, seeds (e.g. sesame, linseed, or hemp), might provide nutritional qualities and have lower environmental impacts.

## NNR Food Group 11: Fats and oils

### Global context

At the global level, 223 mt of fats and oils were produced in 2019 of which 93% were vegetable oils. Palm oil was produced in the largest quantity (75 mt) – accounting for 33% of total production, followed by soybean oil (60 mt), rapeseed oil (24 mt), sunflower oil (20 mt) and pig fat (11 mt) (83). Globally, more than 300 million hectares of land is used for oil crop production (216). The majority (68%) of palm oil is used for human food, and 27% is used for industrial applications including soaps. While palm oil is consumed around the world, Indonesia accounts for 57% of annual production, and Malaysia produces 27%. The amount of land used globally for palm oil production has increased from 4 million hectares in 1980 to 19 million hectares in 2018, and is concentrated across the tropics. Most of this land use is in Malaysia and Indonesia (63%). In Indonesia, palm oil plantations are estimated to account for 23% of deforestation over a 15-year period from 2001 to 2016. Globally, from 2001–2015, oil palm was responsible for around 25% more deforestation in comparison to soy, and around 4 times less deforestation in comparison to cattle (116).

**Fig. 30:**
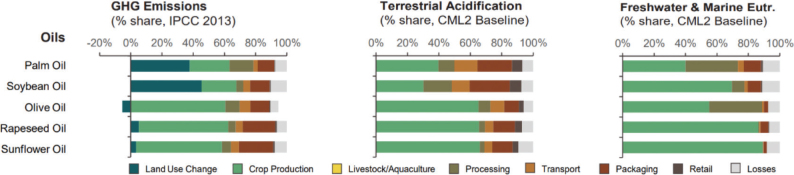
Proportion of environmental impacts from different life cycle stages: Oils (global) (2).

The land use change impacts are reflected in the proportion of environmental impacts across different stages of the life cycle (Fig. 30). For example, palm oil and soybean oil have large GHG emissions associated with land use change. Conversely, olive oil has net negative GHG emissions related to land use change, due to olive trees sequestering carbon during photosynthesis. For olive, rapeseed and sunflower oil, the majority of environmental impacts occur during crop production.

None of the oils listed in Table 31 have the lowest relative impacts across the board. For example, palm oil has the lowest impacts in comparison to the other oils listed, except for acidifying emissions, where soybean oil has a lower impact. This variation across the oils in terms of relative environmental impacts demonstrates the trade-offs involved in assessing environmental sustainability of food and diets. It also omits information that is important when assessing potential shifts, such as growing conditions. For example, palm is a tropical fruit and hence its production location is limited to the tropics.

**Fig. 31:**
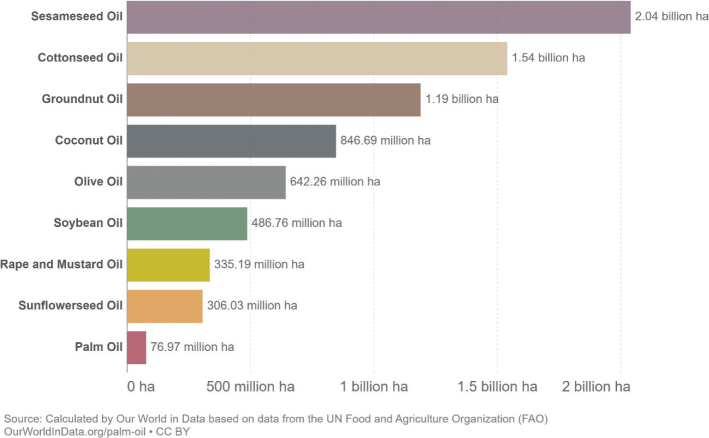
Area of land needed if each oil type alone was to meet global vegetable oil demand, 2018 (216).^31^

Focusing on single metrics, such as land use, when assessing potential to reduce environmental impacts can also be problematic. For example, as demonstrated in Fig. 31, shifting all oil production to palm oil would reduce the overall land requirements for oil production, but as indicated by Table 31, this shift would in turn increase GHGs. It also fails to account for location-specific impacts relating to crop production, for example, growing sunflower seeds in a temperature climate in comparison to clearance of carbon and biodiversity rich forest in the tropics to grow palm oil (the area needed for palm oil equates to a 4-fold increase from the current crop area of 19 million ha). Hence, in some cases, a shift to other oils could potentially have a lower overall impact, for example, replacing some palm oil in Europe with rapeseed or sunflower oil. However, the substitutability of palm oil depends on its intended use – it is a largely unique oil in terms of its use in food products. A more effective strategy would be to reduce the use of palm oil in diets/food manufacture, and also to consider shifting palm oil used for biofuels, as a way to reduce overall demand more substantially (216). Nutritional properties should also be considered, for example, the requirement for poly unsaturated fatty acid rather than saturated fat.

**Table 31:** Average environmental impacts per kg of retail weight: Oils (global) (2)

	Land use (m^2^/kg)	Greenhouse gas emissions (kg CO_2_eq/kg)	Acidifying emissions (g SO_2_eq/kg)	Eutrophying emissions (g PO_4_^3-^eq/kg)	Freshwater (L/kg)
**Soybean Oil**	10.5	6.3	15.7	11.7	415
**Palm Oil**	2.4	7.3	17.5	10.7	6
**Sunflower Oil**	17.7	3.6	28.0	50.7	1,008
**Rapeseed Oil**	10.6	3.8	28.5	19.2	238
**Olive Oil**	26.3	5.4	37.6	37.3	2,142

### Nordic and Baltic context

All countries, with the exception of Estonia which is a net exporter, are dependent on vegetable oil imports to meet national supply. Norway and Sweden have the highest dependency on imports (Table 32). A flexitarian diet that aligns with planetary boundaries up to 2050 (if implemented in combination with medium-ambition technological measures to reduce inputs during food production, and reductions in food loss and waste of 75%) recommends a daily maximum vegetable oil intake of 80g, or 29.2 kg per year, in addition to a maximum intake of 2.5 kg of palm oil per year (as part of an average daily intake of 2,100 kcal) (14). Based on the annual supply data in Table 32, all countries consume below this amount, from 2 to around 15-fold (on a per person basis). Vegetable oil consumption data from national food diaries (i.e. Table 2) is not available for comparison.

Finland, Norway, and Sweden are net importers of animal fats, whereas Denmark, Iceland, and Lithuania are net exporters (Table 33). Denmark, Finland, Iceland, Latvia, Norway, and Sweden all consume a larger amount of animal fats per person in comparison to vegetable oils. Only Estonia has a lower consumption of animal fats in comparison to vegetable oils, on a per person basis (Tables 32 and 33).

**Table 32:** Vegetable oil^32^ production, trade and consumption across the Nordic and Baltic countries in 2019 (84)

Country	Production (1,000 tonnes)	Imports (1,000 tonnes)	Exports (1,000 tonnes)	Balance (1,000 tonnes)	Food supply (Kg/person/year)
**Denmark**	247	724	368	603	2
**Estonia**	53	34	71	16	13
**Finland**	70	178	43	205	3
**Iceland**	2	10	2	10	11
**Latvia**	68	103	26	145	16
**Lithuania**	128	239	97	270	10
**Norway**	96	571	100	567	5
**Sweden**	139	880	175	844	8

**Table 33:** Animal fat^33^ production, trade and supply across the Nordic and Baltic countries in 2019 (84)

Country	Production (1,000 tonnes)	Imports (1,000 tonnes)	Exports (1,000 tonnes)	Balance (1,000 tonnes)	Food supply (Kg/person/year)
**Denmark**	269	184	301	152	19
**Estonia**	9	4	5	8	5
**Finland**	134	69	45	158	15
**Iceland**	63	8	49	22	18
**Latvia**	44	10	10	44	22
**Lithuania**	87	27	76	38	10
**Norway**	117	194	91	220	11
**Sweden**	172	45	25	192	17

#### Considerations for reducing environmental impacts of oil and fat consumption in Nordic and Baltic countries

The main considerations are:

- Consider the overall consumption needed in healthy plant rich diets.
- Shift from animal to (mostly unsaturated) plant-based fats for human consumption.
- Focus consumption on local (e.g. rapeseed, sunflower) over imported palm or soy oil if the environmental impacts are lower and health impacts are equal or improved.

## NNR Food Group 12: Sweets and confectioneries

### Global context

Due to a lack of environmental impact assessment data on processed food products (which are generically covered in chapter 15), this chapter focuses on some of the key commodity crops that are major constituents of sweets and confectioneries – sugar and cocoa. In addition, a number of important foods relevant to sweets and confectioneries are covered in other chapters, including cereals (chapter 3), dairy (chapter 9), eggs (chapter 10), and oils and fats (chapter 11).

Sugar cane is produced in higher quantities than any other crop. At the global level, 2 billion tonnes of sugar cane, 280 million tonnes of sugar beet and 6 million tonnes of cocoa beans were produced in 2019. Around 3.5% of sugar cane and 6% of sugar beet was used for farmed animal feed. Sugar cane is generally grown in tropical and subtropical regions. Brazil produced the most sugar cane in 2019 (752 mt), followed by India (405 mt) and Thailand (131 mt). Sugar beet production is concentrated in temperate regions of the northern hemisphere. Russia produced the most sugar beet in 2019 (54 mt), followed by France (38 mt) and Germany (30 mt). Cocoa beans are generally grown in tropical and subtropical regions. Cote d’Ivoire produced the largest quantity of cocoa beans (2 mt), followed by Ghana (0.8 mt) and Indonesia (0.8 mt) (83). Globally, cocoa production was estimated to be the fourth largest driver of deforestation – and thus biodiversity loss – from 2001 to 2015, contributing a slightly larger share than coffee, and around 20 times less deforestation in comparison to cattle (116).

**Fig. 32:**
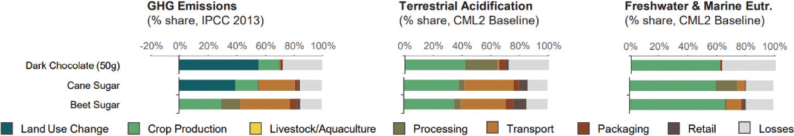
Proportion of environmental impacts from different life cycle stages: Sweets and confectioneries (global) (2).

The land use change impacts are reflected in the proportion of environmental impacts across different stages of the life cycle, with more than half of the GHGs related to dark chocolate resulting from land use change – despite cocoa being a seed from tree fruit. Crop production is most important in terms of eutrophication impacts; however, other stages (mainly transport) are also important for GHGs and acidification impacts of beet and cane sugars (Fig. 32).

On a per unit basis, beet sugar has lower environmental impacts than cane sugar across the spectrum of metrics shown in Table 34. Sugar cane is produced from a grass (sugar beet is a root) and generally requires more processing in comparison to sugar beet, although this does not account for the majority of the difference in impacts.

**Table 34:** Average environmental impacts per kg of retail weight: Sweets and confectioneries (global) (2)

	Land use (m^2^/kg)	Greenhouse gas emissions (kg CO_2_eq/kg)	Acidifying emissions (g SO_2_eq/kg)	Eutrophying emissions (g PO_4_^3-^eq/kg)	Freshwater (L/kg)
**Cane sugar**	2.0	3.2	18.0	16.9	620
**Beet sugar**	1.8	1.8	12.6	5.4	218
**Dark chocolate**	69.0	46.7	46.3	87.1	541

Many of the same issues related to sugar and cocoa production (e.g. land use, land use change, freshwater use, nutrient pollution of surrounding ecosystems, monocultures, intensive farming methods, soil erosion, use of crop protection products, and biodiversity loss), apply to cereal and legume production (see chapters 3 and 5 for more details). An additional issue related to cocoa bean production is a very high (>90%) yield dependency on pollinators (214).

#### Considerations for reducing environmental impacts of sweets and confectioneries consumption in Nordic and Baltic countries

**Fig. 33:**
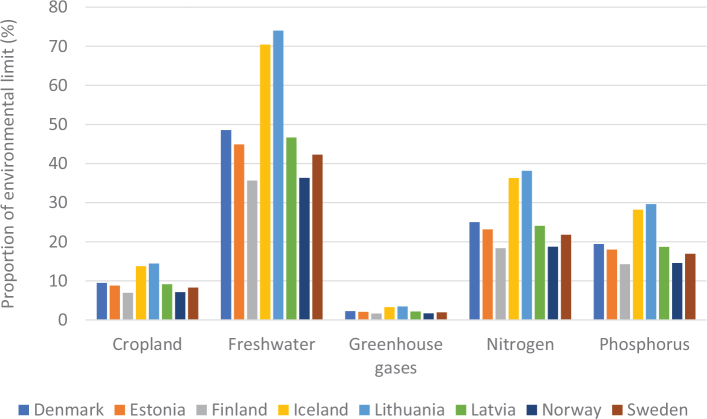
Impacts of sugar consumption across the Nordic and Baltic countries in relation to the food portion of global environmental limits (%). *Source: Global Nutrition Report 2021 (**17**).* The analysis utilises country-specific food consumption and environmental footprint data and relates them to the food portions of global environmental limits that is a global test to assess the impacts if everyone in the world consumed at the given rate. The methods and data are described in Box 1 and presented in Appendices 1–3.

Figure 33 shows the variation in terms of the impacts of sugar consumption across the Nordic and Baltic countries and how they relate to the food portion of global environmental limits. For example, if everyone ate the same amount of sugar as Lithuania it would use over 70% of the global freshwater limit for food consumption. The impacts are highest in terms of freshwater use, but nitrogen and phosphorus use are also substantial, and cropland to a lesser extent.

Annual supply of sugar per person varies among the countries, being 42 kg in Denmark, 20 kg in Estonia, 33 kg in Finland, 30 kg in Iceland, 26 kg in Latvia, 30 kg in Lithuania, 27 kg in Norway and 33 kg in Sweden in 2019 (84).^34^ In comparison, a flexitarian diet that aligns with planetary boundaries up to 2050 (if implemented in combination with medium-ambition technological measures to reduce inputs during food production, and reductions in food loss and waste of 75%) recommends a daily maximum sugar intake of 31 g, or 11.3 kg per year (as part of an average daily intake of 2,100 kcal) (14). Based on the annual supply of sugar, all countries consume above this amount, by around 2 to 4-fold (on a per person basis). Sugar consumption data from national food diaries (i.e. Table 2) is not available for comparison.

The biggest challenge in relation to sweets and confectioneries is reducing their consumption in line with dietary recommendations. However, there are a number of considerations for reducing environmental impacts:

- Replacing sweets and confectioneries as much as possible with a diversity of nutritious foods (such as nuts, seeds, pulses/legumes, grains, and fruits/vegetables) would likely reduce environmental impacts of diets overall, and free up agricultural land and resources that in turn could enable an increased production of such foods while also providing a nutritional and health benefit. Any continued consumption of sweets and confectioneries (i.e. that meet health criteria) should be plant-based (e.g. made without dairy and animal fats where possible to minimise negative environmental impacts) and should diversify ingredients to help promote a diversity of crop production from farming techniques that favour biodiversity and minimise adverse environmental impacts. For example, carob (a nitrogen fixing leguminous crop) could be explored as an alternative to cocoa.
- More understanding of complex food products and supply chains relative to the Nordic and Baltic countries (and their impacts, such as deforestation) would help to shape recommendations on sweets and confectioneries from an environmental sustainability perspective. Sugar taxes that help reduce imports could provide a useful tool if carefully planned.

## NNR Food Group 15: Ultra processed foods

### Global context

The risks and benefits of ultra-processed foods (UPF) have, until recently, largely been understudied. The definition of UPF itself can be contested (217). The NOVA classification system has been endorsed by the WHO/FAO and is a widely accepted definition in the scientific literature (218, 219). NOVA classifies all foods and food products into one of four categories due to the nature and extent of industrial processing to which they have been submitted (unprocessed and minimally processed; processed culinary ingredients; processed foods; and UPF) (217). In this classification, UPFs are ‘ready-to-consume and ready-to-heat formulations, made by combining substances derived from foods with cosmetic additives, typically through a series of industrial processes’ and often contain little or no whole foods. Some examples of UPFs are biscuits, confectionery, reconstituted meats, margarine, and many ready-made meals (220).

The definition of UPF is still contested in some areas, with a resistance to classifying foods as ultra-processed because they contain certain additives, irrespective of their full nutritional profile or an argument that these foods are discretionary and can still play a role in a balanced diet. Nevertheless, there is growing concern that the average global diet has quickly transitioned to a highly processed form. Since the 1950’s, UPFs have been increasingly consumed in high income countries and now contribute to a substantial proportion of energy intake (e.g. more than 50% in the USA and UK; and more than 33% in Australia and France) (221). From around 1980 onwards, following the globalisation of food systems, consumption of UPFs began to increase in middle-income countries and now accounts for 30%, 29%, and 22% of energy intake in Mexico, Chile, and Brazil, respectively (222). UPF consumption is also rising in lower income countries within Asia, Africa, and Latin America (221).

**Fig. 34:**
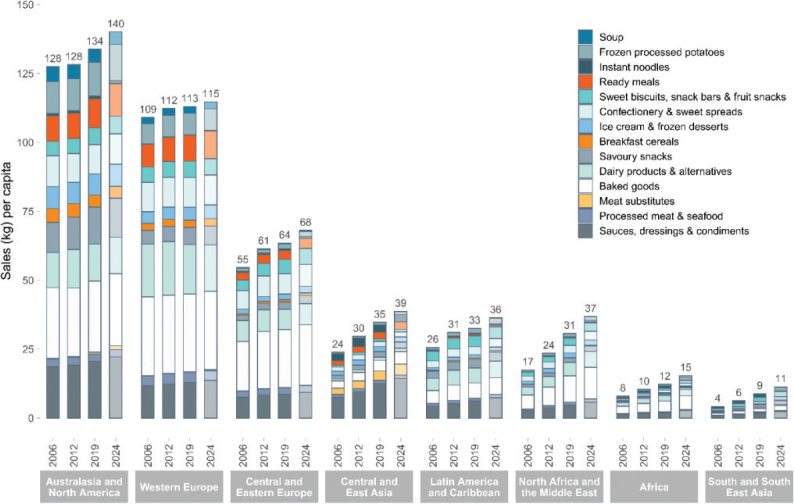
Ultra-processed food sales (kg) per capita by region 2006–2019 with projections to 2024 (223).^35^

A general observation is that as country income rises, greater volumes and a wider variety of UPFs are sold. At first, high-income groups account for the majority of UPF consumption within a country. Then, as a country continues to prosper, and similar to trends in obesity prevalence, low-income groups begin to account for the majority of UPF consumption (223). There are, however, large variations within these trends at regional and country levels. Sales volumes are highest in Australasia, North America, Europe, and Latin America, yet growing most rapidly in Asia, the Middle East, and Africa (Fig. 34). UPF transitions are closely linked with the industrialisation of food systems (the mass production of primary agricultural commodities), technological change (conversion of these commodities into a diverse range of cheaper ingredients available for use in food manufacturing), and globalisation (opening up market opportunities) and diet-related ill-health associated with excessive energy (kcal) consumption or risks associated with non-sugar sweeteners (223).

Currently, there is a paucity of evidence in terms of environmental impacts of UPFs and their role in environmentally sustainable diets (220). One analysis of food consumed in Australia estimated that ‘discretionary foods’, which includes UPFs, accounted for 29.4% of diet-related GHGs (224). An assessment of UK consumption found that UPFs had lower GHGs and were cheaper to buy compared to minimally processed foods, yet there were of a lower nutritional quality (225). An important concern with UPFs is not necessarily processing per se, but their high palatability and ability to encourage overconsumption of energy (kcal) with minimal nutritional benefit. This has direct consequences for the environment. For example, many UPFs contain palm and soy oils, which have substantial negative environmental effects on land use change, such as deforestation (see chapter 11: fats and oils) (220).

The impacts of UPFs on agrobiodiversity are an emerging area of research. One concern is that UPFs displace the cultivation and consumption of fresh and minimally processed foods, and in turn further the reduction of crop diversity as their ingredients tend to be sourced from a few high-yielding plant species grown as global commodities in monocultures (such as sugar, maize, and wheat) (221). The homogeneity of agricultural landscapes reduces opportunities for wildlife by limiting the types of habitats and available resources (3). UPFs containing animal products such as meat further increase their environmental impacts due to the feed requirements for farmed animals (particularly if the feed is sourced from large scale monocultures and limited to a few crop types), in addition to the large environmental impacts of animal farming (see chapter 8 for more information on the inefficiency of and environmental impacts of animal feed and farmed animals) (221).

### Nordic and Baltic context

Taking the global context regarding UPFs, the same concerns would apply in the Nordic context. For example, absolute (system level) vs relative environmental impacts of UPFs, and contribution to land use change and biodiversity loss via imports as consumption rises. Some countries already have a high consumption of UPFs (e.g. Iceland, where UPFs account for 40% of adult energy intake) (226).

To assess UPFs in relation to the Nordic and Baltic countries it is considered essential to define them adequately and consistently. For example, using the NOVA definition might lead to the assumption that processing is generally a negative aspect of food production, and that all ready meals are nutritionally inadequate (which is not the case). It is also unclear how plant-based meat replacement products relate to UPFs, since some are highly processed, and others are less processed. In some cases, such as in Finland, meat replacements made from local grains and pulses, such as fava beans, peas, and oats, have high-quality nutrition profiles in terms of low contents of salt and saturated fat and also have a high-quality amino acid profile. In some cases, processing can also make plant-based ingredients more digestible and thus make their nutrients more bioavailable. Therefore, nutritional adequacy (or inadequacy) may sometimes be problematic to associate with food processing.

Increasing consumption of discretionary/ultra processed foods is mostly considered a health issue in the region, rather than an environmental issue, due to lack of data on environmental impact of these products. Some of the increase in consumption is attributed to a lack of time and knowledge needed for home cooking, while intense marketing and high palatability might also play a role, along with price and availability. Hence, interventions to reduce discretionary/ultra processed food consumption could usefully consider such aspects.^36^

#### Considerations for reducing environmental impacts of ultra processed foods in Nordic and Baltic countries

The biggest challenge in relation to reducing the environmental impacts of UPFs is reducing their consumption across populations that are increasingly accustomed to their lower price, taste, and convenience. However, there are several important considerations for reducing environmental impacts:

- Replacing UPFs, that is, foods that are discretionary in terms of nutritional requirements, as needed with a diversity of nutritious foods (such as nuts, seeds, pulses/legumes, grains, and fruits/vegetables) would likely reduce environmental impacts of diets overall, and a reduced agricultural production of ingredients used in UFPs could allow more cropland to be used for the production of such nutritious foods. Potential barriers to implementation, such as lack of knowledge of ingredients and cooking/preparation methods, should be considered.
- Any consumption of UPFs should not exceed an amount that would result in higher energy, salt, sucrose, or saturated fatty acid contents than recommended, should be plant-based and should diversify ingredients to help promote a diversity of crop production from farming techniques that favour biodiversity and minimise adverse environmental impacts (222).
- More understanding of complex food products and supporting supply chains relative to the Nordic and Baltic countries would help to shape recommendations on UPFs from an environmental sustainability perspective.

## Section 4: Key environmental sustainability considerations for food consumption in the Nordic and Baltic region

This paper’s principal conclusion is that, even though environmental impacts differ between different production systems and region-specific circumstances, the primary determinant of the environmental impact of food consumption is not the production system or place, but what is consumed, and its quantity. In short, substantially reducing the consumption of animal-sourced foods (particularly meat and dairy) is the key approach to reducing adverse environmental impacts at the system, aggregate or absolute level. Radical changes to current consumption patterns are urgently required to provide the best chance of avoiding the worst impacts of climate change and ecosystem destabilisation. We therefore focus our advice for reducing the environmental impacts of food consumption in the Nordic and Baltic countries on measures that could be implemented in the immediate to short term. We recognise that other options might become relevant in the medium to long term, such as effective methods to reduce methane emissions from farmed animals, and novel food production technologies, such as precision fermentation, cell-cultured foods, controlled-environment farming and algae production that could improve the environmental performance of food systems. However, the required change is large and immediate enough to necessitate change in both food production and consumption. Delaying remediation of today’s burgeoning environmental impacts of the food system in the hope they will be resolved by technologies in the future is a very high-risk strategy that should be avoided.

The overarching advice for all countries, in line with the current body of scientific literature, is to shift to more plant-based dietary patterns – and avoid food waste. From this, we can deduce that there is high potential and necessity to shift food consumption across the Nordic and Baltic countries to minimise its environmental impacts. The extent to which this is necessary depends on current consumption patterns. More specifically, we suggest the following as priority interventions for increasing the environmental sustainability of food consumption:

- Reduce meat and dairy consumption substantially and increase the consumption of legumes/pulses, whole grains, vegetables, fruits, nuts, and seeds – and explore the potential to increase consumption of wild berries (while potential impacts on wildlife should also be considered) and cultivation of legumes/pulses, vegetables, and grains within the region.
- Explore potential seafood shifts from species with higher impacts to species with lower impacts (e.g. seaweed, bivalves), and explore options for direct human consumption of omega-3 fatty acids, for example, microalgae as a replacement for salmon. Due to the potentially large-scale impacts on ecosystems and the largely unknown nature of fish stocks globally, a precautionary approach to the fish and seafood food group is essential – particularly in relation to pursuing an increase in consumption. More information is required before any targeted increase in fish consumption in the Nordic and Baltic countries could be justified from an environmental sustainability perspective, and also from a food security/risk perspective.
- Explore options that support a reduction in consumption of animal products and have potential to increase provision of plant-based foods through feed-to-food shifts. This is relevant to cereals and pulses, as well as nuts, vegetables, and fruits.

In addition to the specific suggestions above, we identify a number of overarching, broader points for consideration:

- Identifying suitable options for reducing the environmental impacts of dietary consumption should be in conjunction with a nutritional assessment to ensure that food shifts align with nutritional adequacy and positive health impacts at the dietary level. This is explored in a subsequent paper in this series (60).
- Changes to demand *and* supply are needed to align food systems with environmental thresholds. In this context, where consumption of fruits and vegetables must increase, shifting production methods could help to further reduce environmental impacts (particularly water and fertiliser use). In addition, fruits and vegetables that require less resources to produce could be prioritised if in alignment with the requirements of a healthy diet.
- The literature suggests that cultivation methods such as those used in organic production (or similar) result in greater biodiversity benefits. At the global level it is only possible to convert agricultural production to such methods in conjunction with substantial shifts in demand to mostly plant-based diets (3, 70, 227).
- The total impacts of food consumption must be considered, including imports, and the impacts must include biodiversity. Impacts must be considered in terms of environmental thresholds at the local, regional, and global levels.
- National strategies that facilitate changes to food consumption and production (including exports) should consider the complexity of trade-offs and location-specific impacts and contexts, and implications for trade. An important part of such strategies would be a comprehensive review to identify and explore the range of issues including human health and social impacts, animal welfare, and current and emerging threats, for example, antimicrobial resistance and zoonotic-driven pandemics.
- A national land use assessment could inform optimal land uses for meeting a range of environmental goals, also accounting for the environmental impacts of food imports in producer countries. One important inclusion would be an assessment of pasture land in terms of its current value and necessity for food production and the potential for alternative food production such as agroforestry of tree nuts, or other uses to help meet social and environmental goals (i.e. mitigating and adapting to climate change and restoring biodiversity), such as restoring portions of native ecosystems (e.g., Boreal forest in countries with some territory in the Boreal forest zone including Norway, Sweden, Finland, Estonia, Lithuania, and Latvia).
- The national strategies and land use assessments could usefully apply the five key considerations identified in the first paper in this series as consistent framings: 1) Consider the thresholds, 2) Consider the system, 3) Consider the variables, 4) Consider the context, and 5) Consider the spill over (13).
- The overarching approach to reducing the environmental impacts of food consumption must not be limited to the realities of today’s market and other socio-political determinants. The potential impacts and risks of environmental destabilisation on food production and supply (including those related to climate change and biodiversity loss, such as temperature extremes, droughts, and floods) should also be considered.
- While urgent and fundamental changes to food production and consumption are required to help meet climate change and biodiversity goals (81), tackling such issues does not remove the need for urgent reform in other sectors, including energy. Instead, transformation of food systems must be incorporated as one part of a comprehensive ‘green transition’ plan that includes all systems.

## Footnotes

1 Based on estimates of food demand from the Food and Agriculture Organization of the United Nations and a database of country and food group-specific environmental footprints. The target values for sustainable food production that would be in line with Sustainable Development Goals were specified by and adapted from the EAT-Lancet Commission. Note: In this test, regional diets in 2010 and 2018 are universally adopted and compared to global environmental targets. Source: Ref. (15)

2 Notes: Planetary boundaries define the threshold related to global environmental processes beyond which humanity should not go. Planetary boundaries align with the targets for sustainable food production as set out by the Sustainable Development Goals. If globalised impacts exceed 100% of the planetary boundary, the dietary pattern can be considered unsustainable in light of global environmental targets, and disproportionate in the context of an equitable distribution of environmental resources and mitigation efforts. The methods and data are described in Box 1, and presented in Appendices 1–2.

3 Calculated by dividing agricultural land area by total land area for each country, multiplied by 100 (2019 data on land use: https://www.fao.org/faostat/en/#data/RL). Land area is defined in the FAOSTAT as: ‘Country area excluding area under inland waters and coastal waters.’

4 Land Use 2019. FAOSTAT. https://www.fao.org/faostat/en/#data/RL. For an overview of agricultural land use types in the FAOSTAT, see Figure 1: Land use statistics and indicators (fao.org)

5 Land used for cultivation of crops. The total of areas under ‘Arable land’ and ‘Permanent crops’. Arable land is the total of areas under temporary crops, temporary meadows and pastures, and land with temporary fallow. Arable land does not include land that is potentially cultivable but is not normally cultivated.

6 Land used permanently (five years or more) to grow herbaceous forage crops through cultivation or naturally (wild prairie or grazing land). Permanent meadows and pastures on which trees and shrubs are grown should be recorded under this heading only if the growing of forage crops is the most important use of the area. Measures may be taken to keep or increase productivity of the land (i.e. use of fertilizers, mowing or systematic grazing by domestic animals.) This class includes: • Grazing in wooded areas (agroforestry areas, for example) • Grazing in shrubby zones (heath, maquis, garigue) • Grassland in the plain or low mountain areas used for grazing: land crossed during transhumance where the animals spend a part of the year (approximately 100 days) without returning to the holding in the evening: mountain and subalpine meadows and similar; and steppes and dry meadows used for pasture.

7 Land cultivated with long-term crops which do not have to be replanted for several years (such as cocoa and coffee), land under trees and shrubs producing flowers (such as roses and jasmine), and nurseries (except those for forest trees, which should be classified under ‘Forestry’). Permanent meadows and pastures are excluded from land under permanent crops.

8 Land used for crops with a less-than-one-year growing cycle, which must be newly sown or planted for further production after the harvest. Some crops that remain in the field for more than one year may also be considered as temporary crops for example asparagus, strawberries, pineapples, bananas and sugar cane. Multiple-cropped areas are counted only once.

9 Land temporarily cultivated with herbaceous forage crops for mowing or pasture. A period of less than five years is used to differentiate between temporary and permanent meadows and pastures.

10 Of which 626,000 ha are cultivated and 102,000 are natural growing.

11 Of which 20,000 ha are cultivated and 159,100 are natural growing.

12 Where consumption data is unavailable, the space in the table has been left empty.

13 The target values for sustainable food production are in line with the Sustainable Development Goals specified by and adapted from the EAT-Lancet Commission (25, 42).

14 National Dietary Guideline year and country used in Figure 3: Denmark, 2015; Estonia, 2015; Latvia, 2008; Lithuania, 2018; Finland, 2014; Sweden, 2015; Iceland, 2014; Norway, 2014. The methods and data are described in Box 1, and presented in Appendices 1–3.

15 Grains are an average of wheat, maize, oats, and flooded rice. From figure caption for S13 in (2).

16 Percentage of total national supply as shown in Table 4 under ‘balance’.

17 Calculated from total feed amount in 2019 divided by total food amount in 2019, using FBS data.

18 Note – tomatoes are fruits but are grouped here with vegetables as they are most commonly consumed as a vegetable.

19 This figure does not include starchy root vegetables.

20 Calculated from total feed amount divided by total food amount. NA accounts for cases where consumption equalled zero.

21 Please note: This information was provided by regional experts during a series of workshops and has not been verified.

22 Includes beans, peas, pulses (other), and soybeans. Pulses (other) is a group which includes broad beans, chickpeas, and lentils.

23 Based on data from 1,690 fish farms.

24 Based on data from 1,690 fish farms.

25 Based on data from 1,690 fish farms.

26 Based on data from 1,690 fish farms.

27 Includes all fish species and major seafood commodities, including crustaceans, cephalopods and other mollusc species. Data is based on per capita food supply at the consumer level, but does not account for food waste at the consumer level.

28 Pelagic fish include: herring, mackerel, capelin, blue whiting, bluefin tuna. Demersal fish include: cod, haddock, whiting, hake, pollock and plaice. Freshwater fish include: salmon (technically an anadromous species), trout, bass, carp, bream, eel, and sturgeon. Crustaceans include: crabs, lobsters, crayfish, shrimp, krill, and prawns. Molluscs include: mussels, scallops, oysters, clam and whelk. Cephalopods include: octopuses, squid, cuttlefish and nautiluses.

29 Please note: This information was provided by regional experts during a series of workshops and has not been verified.

30 Data based on a meta-analysis of life cycle assessment studies across the supply chain.

31 Land use per crop assumes that all vegetable oil is produced from that crop alone.

32 Includes: Rape and mustard (majority), palm, soya bean, sunflower, olive, coconut, groundnut, sesame, and ricebran.

33 Includes: cream (majority), butter, fish body oil and fish liver oil.

34 Food supply quantity (per person per year) of sugar (raw equivalent) in 2019.

35 Analysis includes all Nordic and Baltic countries except Iceland.

36 Please note: This information was provided by regional experts during a series of workshops and has not been verified.

## Appendix 1 Overview of global health and environmental targets and their derivation

**Table:**

Global targets	Comment	Implementation
Paris Climate Agreement	The Paris Agreement’s long-term goal is to keep the increase in global average temperature to well below 2°C above pre-industrial levels; and to limit the increase to 1.5°C, since this would substantially reduce the risks and effects of climate change. The goal is reflected in Sustainable Development Goals (SDG) 13 and in the planetary boundary for climate change.	The target for agricultural emissions in line with the 2°C target was derived as 4.7 (4.3–5.3) GtCO2-eq (Wollenberg et al, 2016; Sprirgmann et al, 2018). We adjusted this value for the proportion of emissions related specifically to food consumption (92% of emissions of the whole food system, according to Springmann et al, 2018).
Aichi Biodiversity Targets	Target: 5: By 2020, the rate of loss of all natural habitats, including forests, is at least halved and where feasible brought close to zero, and degradation and fragmentation is. significantly reduced. The target is related to SDG 15 and the planetary boundary for land - system change.	Conrtribute to target by not increasing pressure to convert natural land into cropland (or pastures), in line with the food-related planetary boundary for land-systems change (Steffen et al, 2015; Springmann et al, 2018). The planetary boundary value was set to the extent of current croplard (+/-16%). We internally recalculated the value for consistency with the baseline parameters and our focus or food available for consumption (9.9Mkm2, 8.3–115)
SDG target on water withdrawals	SDG 6.4: By 2030_,_ substantially increase water-use efficiency across all sectors and en sure sustainable withdrawals and supply of freshwater to address water scarcity and substantially reduce the number of people suffering from water scarcity. The goal is in line with the planetary boundary for fresh-water use.	Adapt the food-related planetary-boundary target of maintaining environmental flow requirements by limiting agricultural fresh-water use to below 2,000km3, with a range of 800-3,350 km3 (Springmann et al. 2018). We adjusted the value for the proportion of the food system attributed to diets (1.600km3, 640–2,600).
SDG target on nutrient pollution	SDG 14.1: By 2025. prevent and significantly reduce marine pollution of all kinds, in particular from land-based activities, including marine debris and nutrient pollution.The goal is in line with the planetary boundary for biogeochemioal flows of nitrogen and phosphorus.	Adopt the food-related planetary-boundary target for nitrogen and phosphorus application in line with limiting eutrophication risk (de Vries et al, 2013; Springmann et al, 2018). We recalculated the value for our focus on consumption-related impacts by applying the original risk fractions to estimates of baseline use, which yielded target values of 51TgN (38–83) and 11TgP (5.6–12.9).

Source: SI Table 4. https://globalnutritionreport.org/reports/2021-global-nutrition-report/appendix-chapter-2-methodology-and-data-sources/

## Appendix 2 Environmental footprints of food commodities (per kg of product), 2010 and 2050

**Table:**

Food groups	Greenhouse gas emissions (kgCO2eq/kg)	Greenhouse gas emissions (kgCO2eq/kg)	Cropland use (m2/kg)	Cropland use (m2/kg)	Fresh-water use (m3/kg)	Fresh-water use (m3/kg)	Nitrogen use (kgN/t)	Nitrogen use (kgN/t)	Phosphorus use (kgP/t)	Phosphorus use (kgP/t)
	2010	2050	2010	2050	2010	2050	2010	2050	2010	2050
Wheat	0.23	0.21	3.36	2.46	0.49	0.37	28.73	19.78	4.39	2.01
Rice	1.18	0.90	3.51	2.78	1.07	0.89	36.64	25.07	5.20	2.28
Maize	0.19	0.17	1.98	1.40	0.15	0.12	22.77	14.36	3.57	1.55
Other grains	0.29	0.22	6.17	4.43	0.17	0.14	16.39	9.82	2.72	0.97
Roots	0.07	0.06	0.69	0.52	0.04	0.04	3.60	2.07	0.71	0.30
Legumes	0.23	0.19	11.11	6.39	0.94	0.61	0.00	0.00	0.00	0.00
Soybeans	0.12	0.09	3.95	3.14	0.14	0.15	2.75	1.75	5.88	3.17
Nut6 & seeds	0.69	0.65	6.39	5.13	0.43	0.33	14.16	10.84	2.10	1.17
Vegetables	0.06	0.07	0.49	0.34	0.09	0.06	9.55	6.32	1.67	0.81
Oil crops	0.70	0.64	3.12	2.37	0.22	0.19	13.33	8.50	2.86	1.32
Fruits (temperate)	0.08	0.08	1.18	0.97	0.33	0.28	12.73	8.57	1.91	0.92
Fruits (tropical)	0.09	0.10	0.94	0.62	0.32	0.23	10.27	6.10	1.58	0.70
Fruits (starchy)	0.11	0.10	0.88	0.59	0.11	0.08	6.15	3.76	1.05	0.48
Sugar	0.19	0.19	1.67	1.35	1.22	0.88	22.34	15.26	3.84	1.86
Palm oil	1.85	2.03	3.10	2.39	0.00	0.00	22.34	16.29	3.57	1.85
Vegetable oil	0.67	0.63	10.31	8.46	0.47	0.45	42.73	28.19	11.47	5.66
Beef	36.78	40.36	4.21	2.78	0.22	0.17	27.29	17.16	5.36	2.29
Lamb	36.73	37.21	6.24	4.48	0.49	0.42	27.52	21.82	4.94	2.47
Pork	3.14	3.25	6.08	4.90	0.35	0.29	51.52	34.19	3.87	4.05
Poultry	1.45	1.39	6.59	5.18	0.40	0.36	50.20	36.00	9.02	4.35
Eggs	1.61	1.48	6.86	5.19	0.44	0.39	51.22	35.09	3.81	4.18
Milk	1.28	1.39	1.34	1.01	0.08	0.03	6.32	4.63	1.58	0.78
Shellfish	0.03	0.04	0.36	0.46	0.03	0.04	2.19	2.39	0.50	0.40
Fish (freshwoter)	0.12	0.12	1.51	1.37	0.10	0.10	11.26	8.39	2.37	1.29
Fish (pelagic)	0.00	0.00	0.00	0.00	0.00	0.00	0.00	0.00	0.00	0.00
Fish (demersal)	0.01	0.01	0.13	0.20	0.01	0.01	0.75	0.99	0.19	0.18

Notes: Values shown are global averages per kilogram of product. Footprints for animal products represent feed- related impacts, except for greenhouse gas emissions of livestock which also have a direct component. Footprints for fish and seafood represent feed-related impacts of aquaculture production weighted by total production volumes. The global overages account for expected efficiency improvements, such as improved feed for livestock end changes in production by 2050. such as increases in extensive beef production in middle-income countries. The analysis is based on country-specific values.

Source: SI Table 3. https://globalnutritionreport.org/reports/2021-global-nutrition-report/appendix-chapter-2-methodology-and-data-sources/. Note, future years factor in improvements in technologies and management practices, including reductions in food loss and waste, along a middle-of-the-road socioeconomic development pathway. The environmental footprint data was also used in Springmann, M et al (2020) The healthiness and sustainability of national and global food based dietary guidelines: modelling study. BMJ https://doi.org/10.1136/bmj.m2322.

## Appendix 3 Food consumption data (g/day) for Nordic and Baltic countries used in environmental analyses

**Table:**

	Beef	Lamb	Poultry	Pork	Eggs	Fish	Milk	Legumes	Fruit	Vegetables	Nuts/seeds	Whole grains	Sugar	Oils
**Denmark**	55.7	3.6	39.6	36.1	40.8	28.5	702.7	2.9	192.1	210.7	10.5	84.2	85.1	20.0
**Estonia**	26.0	1.5	35.7	66.4	29.3	18.2	624.8	8.8	134.8	198.5	6.7	27.7	72.9	19.5
**Finland**	33.7	4.2	32.1	59.5	21.9	43.9	978.8	3.3	161.7	148.2	6.2	80.4	51.3	26.7
**Latvia**	12.1	1.2	37.5	74.1	35.6	33.2	567.5	0.2	86.0	190.1	7.1	27.5	56.9	33.2
**Lithuania**	8.4	0.9	42.1	83.0	32.2	53.6	731.5	9.6	84.1	182.4	4.6	25.2	72.9	24.1
**Norway**	35.0	11.7	30.4	39.9	27.7	66.0	664.4	11.9	232.8	133.9	9.6	85.2	62.0	47.2
**Sweden**	42.6	5.9	27.9	62.8	30.9	38.0	892.8	5.2	210.5	160.5	14.5	89.3	63.7	44.2

Notes: Baseline food consumption was estimated by adopting estimates of food availability from the FAO’s food balance sheets, and adjusting those for the amount of food wasted at the point of consumption. A three-year average of food availability data around the year 2010 (2009, 2010 and 2011) was derived, to be consistent with the data on food waste, and the data on the environmental impacts of food production (Appendix 2).

Source: Supplementary data to Springmann M, et al (2020) The healthiness and sustainability of national and global food based dietary guidelines: modelling study. BMJ https://doi.org/10.1136/bmj.m2322. Benchmark (BMK) mean consumption data for each country and food extracted from tab ‘cons_data’ in FBDG_data_file.xlsx. Available to download: https://ora.ox.ac.uk/objects/uuid:bbf756dd-5556-41f6-b969-a9bee18cdb21/files/d9p2909776
